# Supplementary material for: Is experience of the HIV/AIDS epidemic associated with responses to COVID-19? Evidence from the Rural Malawi
Source: PLoS One. 2023 Oct 25;18(10):e0292378. doi: 10.1371/journal.pone.0292378 (PMC10599567; doi:10.1371/journal.pone.0292378)
Supplement: S1 File — (PDF) [file pone.0292378.s002.pdf]

Cv1 1a Resp Info And Call Logging

Respid

____

Respondentid

____

Region of residence

- ◯ Mchinji
- ◯ Balaka
- ◯ Rumphi
- ◯ Dedza
- ◯ Dowa
- ◯ Kasungu
- ◯ Lilongwe
- ◯ Nkhotakota
- ◯ Ntcheu
- ◯ Ntchisi
- ◯ Salima
- ◯ Chitipa
- ◯ Karonga
- ◯ Likoma
- ◯ Mzimba
- ◯ Nkhata Bay
- ◯ Blantyre
- ◯ Chikwawa
- ◯ Chiradzulu
- ◯ Machinga
- ◯ Mangochi
- ◯ Mulanje
- ◯ Mwanza
- ◯ Nsanje
- ◯ Thyolo
- ◯ Phalombe
- ◯ Zomba
- ◯ Neno

Village ID

____

Village name

____

Name of Respondent

____

Nickname of Respondent

____

Head of compound

____

Name of R's father

____

Name of Village Headman

____

2019 (M11) age expected age (based on prior MLSFH infos)	__________________________________
Respondent's gender:	◯ Male<br>◯ Female
Select Language for the Survey	◯ Chichewa<br>◯ Chitumbuka<br>◯ Chiyao
Prior survey year	__________________________________
Birth village	__________________________________
Birth village	◯ Mchinji<br>◯ Balaka<br>◯ Rumphi<br>◯ Dedza<br>◯ Dowa<br>◯ Kasungu<br>◯ Lilongwe<br>◯ Nkhotakota<br>◯ Ntcheu<br>◯ Ntchisi<br>◯ Salima<br>◯ Chitipa<br>◯ Karonga<br>◯ Likoma<br>◯ Mzimba<br>◯ Nkhata Bay<br>◯ Blantyre<br>◯ Chikwawa<br>◯ Chiradzulu<br>◯ Machinga<br>◯ Mangochi<br>◯ Mulanje<br>◯ Mwanza<br>◯ Nsanje<br>◯ Thyolo<br>◯ Phalombe<br>◯ Zomba<br>◯ Neno
Marital status (1 = marr, 2 = sep, 3 = div, 4 = wid, 5 = never m)	◯ Married/living together<br>◯ Separated<br>◯ Divorced<br>◯ Widowed<br>◯ Never married
Marital status (string)	__________________________________
Number of wives (from most recent coversheet)	__________________________________
ID of spouse 1	__________________________________

ID of spouse 2 __________________________________

ID of spouse 3 __________________________________

ID of spouse 4 __________________________________

ID of spouse 5 __________________________________

Name of spouse 1 __________________________________

Name of spouse 2 __________________________________

Name of spouse 3 __________________________________

Name of spouse 4 __________________________________

Name of spouse 5 __________________________________

Phone 1 (existing number) __________________________________

Phone 2 (existing number) __________________________________

Phone 3 (existing number) __________________________________

Phone 1 (existing number)

- ◯ Own (Respondent)
- ◯ Child
- ◯ Spouse
- ◯ Household member
- ◯ Relative outside of household
- ◯ Friend/neighbor
- ◯ Secondary Contact Person
- ◯ Other/Unknown relationship

Phone 2 (existing number)

- ◯ Own (Respondent)
- ◯ Child
- ◯ Spouse
- ◯ Household member
- ◯ Relative outside of household
- ◯ Friend/neighbor
- ◯ Secondary Contact Person
- ◯ Other/Unknown relationship

Phone 3 (existing number)	◯ Own (Respondent)<br>◯ Child<br>◯ Spouse<br>◯ Household member<br>◯ Relative outside of household<br>◯ Friend/neighbor<br>◯ Secondary Contact Person<br>◯ Other/Unknown relationship
Phone 1 (existing number)	__________________________
Phone 2 (existing number)	__________________________
Phone 3 (existing number)	__________________________
Crit for inclusion in M11 (2019) data collection	__________________________
HH memb 1: Respondent	__________________________
HH memb 2: Name of listed person	__________________________
HH memb 3: Name of listed person	__________________________
HH memb 4: Name of listed person	__________________________
HH memb 5: Name of listed person	__________________________
HH memb 6: Name of listed person	__________________________
HH memb 7: Name of listed person	__________________________
HH memb 8: Name of listed person	__________________________
HH memb 9: Name of listed person	__________________________
HH memb 10: Name of listed person	__________________________
HH memb 11: Name of listed person	__________________________
HH memb 12: Name of listed person	__________________________

HH memb 13: Name of listed person __________________________________

HH memb 14: Name of listed person __________________________________

HH memb 15: Name of listed person __________________________________

HH memb 16: Name of listed person __________________________________

HH memb 17: Name of listed person __________________________________

HH memb 18: Name of listed person __________________________________

HH memb 19: Name of listed person __________________________________

HH memb 20: Name of listed person __________________________________

HH memb 21: Name of listed person __________________________________

HH memb 22: Name of listed person __________________________________

Source of HH roster information (MLSFH wave) __________________________________

HH memb 1: line number in source __________________________________

HH memb 2: line number in source __________________________________

HH memb 3: line number in source __________________________________

HH memb 4: line number in source __________________________________

HH memb 5: line number in source __________________________________

HH memb 6: line number in source __________________________________

HH memb 7: line number in source __________________________________

HH memb 8: line number in source

____

HH memb 9: line number in source

____

HH memb 10: line number in source

____

HH memb 11: line number in source

____

HH memb 12: line number in source

____

HH memb 13: line number in source

____

HH memb 14: line number in source

____

HH memb 15: line number in source

____

HH memb 16: line number in source

____

HH memb 17: line number in source

____

HH memb 18: line number in source

____

HH memb 19: line number in source

____

HH memb 20: line number in source

____

HH memb 21: line number in source

____

HH memb 22: line number in source

____

HH memb 1: Respondent

- ◯ Respondent
- ◯ Wife/husband
- ◯ Son/daughter
- ◯ Father/mother
- ◯ Grandchild
- ◯ Grandparent
- ◯ Mother/father-in-law
- ◯ Son/daughter-in-law
- ◯ Brother/sister-in-law
- ◯ Paternal aunt/uncle
- ◯ Maternal aunt/uncle
- ◯ Sister/brother
- ◯ Cousin
- ◯ Nephew/niece
- ◯ Step-child/half-brother/sister
- ◯ Co-wife
- ◯ Boyfriend/Girlfriend, including PTM
- ◯ Other not related through blood or marriage (friends)
- ◯ Step-mother/step-father
- ◯ Former wife/husband
- ◯ Former boyfriend/girlfriend, including former PTM
- ◯ NOT part of this family/household
- ◯ Don't know person

HH memb 2: Name of listed person

- ◯ Respondent
- ◯ Wife/husband
- ◯ Son/daughter
- ◯ Father/mother
- ◯ Grandchild
- ◯ Grandparent
- ◯ Mother/father-in-law
- ◯ Son/daughter-in-law
- ◯ Brother/sister-in-law
- ◯ Paternal aunt/uncle
- ◯ Maternal aunt/uncle
- ◯ Sister/brother
- ◯ Cousin
- ◯ Nephew/niece
- ◯ Step-child/half-brother/sister
- ◯ Co-wife
- ◯ Boyfriend/Girlfriend, including PTM
- ◯ Other not related through blood or marriage (friends)
- ◯ Step-mother/step-father
- ◯ Former wife/husband
- ◯ Former boyfriend/girlfriend, including former PTM
- ◯ NOT part of this family/household
- ◯ Don't know person

HH memb 3: Name of listed person

- ◯ Respondent
- ◯ Wife/husband
- ◯ Son/daughter
- ◯ Father/mother
- ◯ Grandchild
- ◯ Grandparent
- ◯ Mother/father-in-law
- ◯ Son/daughter-in-law
- ◯ Brother/sister-in-law
- ◯ Paternal aunt/uncle
- ◯ Maternal aunt/uncle
- ◯ Sister/brother
- ◯ Cousin
- ◯ Nephew/niece
- ◯ Step-child/half-brother/sister
- ◯ Co-wife
- ◯ Boyfriend/Girlfriend, including PTM
- ◯ Other not related through blood or marriage (friends)
- ◯ Step-mother/step-father
- ◯ Former wife/husband
- ◯ Former boyfriend/girlfriend, including former PTM
- ◯ NOT part of this family/household
- ◯ Don't know person

HH memb 4: Name of listed person

- ◯ Respondent
- ◯ Wife/husband
- ◯ Son/daughter
- ◯ Father/mother
- ◯ Grandchild
- ◯ Grandparent
- ◯ Mother/father-in-law
- ◯ Son/daughter-in-law
- ◯ Brother/sister-in-law
- ◯ Paternal aunt/uncle
- ◯ Maternal aunt/uncle
- ◯ Sister/brother
- ◯ Cousin
- ◯ Nephew/niece
- ◯ Step-child/half-brother/sister
- ◯ Co-wife
- ◯ Boyfriend/Girlfriend, including PTM
- ◯ Other not related through blood or marriage (friends)
- ◯ Step-mother/step-father
- ◯ Former wife/husband
- ◯ Former boyfriend/girlfriend, including former PTM
- ◯ NOT part of this family/household
- ◯ Don't know person

HH memb 5: Name of listed person	◯ Respondent<br>◯ Wife/husband<br>◯ Son/daughter<br>◯ Father/mother<br>◯ Grandchild<br>◯ Grandparent<br>◯ Mother/father-in-law<br>◯ Son/daughter-in-law<br>◯ Brother/sister-in-law<br>◯ Paternal aunt/uncle<br>◯ Maternal aunt/uncle<br>◯ Sister/brother<br>◯ Cousin<br>◯ Nephew/niece<br>◯ Step-child/half-brother/sister<br>◯ Co-wife<br>◯ Boyfriend/Girlfriend, including PTM<br>◯ Other not related through blood or marriage (friends)<br>◯ Step-mother/step-father<br>◯ Former wife/husband<br>◯ Former boyfriend/girlfriend, including former PTM<br>◯ NOT part of this family/household<br>◯ Don't know person
HH memb 6: Name of listed person	◯ Respondent<br>◯ Wife/husband<br>◯ Son/daughter<br>◯ Father/mother<br>◯ Grandchild<br>◯ Grandparent<br>◯ Mother/father-in-law<br>◯ Son/daughter-in-law<br>◯ Brother/sister-in-law<br>◯ Paternal aunt/uncle<br>◯ Maternal aunt/uncle<br>◯ Sister/brother<br>◯ Cousin<br>◯ Nephew/niece<br>◯ Step-child/half-brother/sister<br>◯ Co-wife<br>◯ Boyfriend/Girlfriend, including PTM<br>◯ Other not related through blood or marriage (friends)<br>◯ Step-mother/step-father<br>◯ Former wife/husband<br>◯ Former boyfriend/girlfriend, including former PTM<br>◯ NOT part of this family/household<br>◯ Don't know person

HH memb 7: Name of listed person	◯ Respondent<br>◯ Wife/husband<br>◯ Son/daughter<br>◯ Father/mother<br>◯ Grandchild<br>◯ Grandparent<br>◯ Mother/father-in-law<br>◯ Son/daughter-in-law<br>◯ Brother/sister-in-law<br>◯ Paternal aunt/uncle<br>◯ Maternal aunt/uncle<br>◯ Sister/brother<br>◯ Cousin<br>◯ Nephew/niece<br>◯ Step-child/half-brother/sister<br>◯ Co-wife<br>◯ Boyfriend/Girlfriend, including PTM<br>◯ Other not related through blood or marriage (friends)<br>◯ Step-mother/step-father<br>◯ Former wife/husband<br>◯ Former boyfriend/girlfriend, including former PTM<br>◯ NOT part of this family/household<br>◯ Don't know person
HH memb 8: Name of listed person	◯ Respondent<br>◯ Wife/husband<br>◯ Son/daughter<br>◯ Father/mother<br>◯ Grandchild<br>◯ Grandparent<br>◯ Mother/father-in-law<br>◯ Son/daughter-in-law<br>◯ Brother/sister-in-law<br>◯ Paternal aunt/uncle<br>◯ Maternal aunt/uncle<br>◯ Sister/brother<br>◯ Cousin<br>◯ Nephew/niece<br>◯ Step-child/half-brother/sister<br>◯ Co-wife<br>◯ Boyfriend/Girlfriend, including PTM<br>◯ Other not related through blood or marriage (friends)<br>◯ Step-mother/step-father<br>◯ Former wife/husband<br>◯ Former boyfriend/girlfriend, including former PTM<br>◯ NOT part of this family/household<br>◯ Don't know person

HH memb 9: Name of listed person

- ◯ Respondent
- ◯ Wife/husband
- ◯ Son/daughter
- ◯ Father/mother
- ◯ Grandchild
- ◯ Grandparent
- ◯ Mother/father-in-law
- ◯ Son/daughter-in-law
- ◯ Brother/sister-in-law
- ◯ Paternal aunt/uncle
- ◯ Maternal aunt/uncle
- ◯ Sister/brother
- ◯ Cousin
- ◯ Nephew/niece
- ◯ Step-child/half-brother/sister
- ◯ Co-wife
- ◯ Boyfriend/Girlfriend, including PTM
- ◯ Other not related through blood or marriage (friends)
- ◯ Step-mother/step-father
- ◯ Former wife/husband
- ◯ Former boyfriend/girlfriend, including former PTM
- ◯ NOT part of this family/household
- ◯ Don't know person

HH memb 10: Name of listed person

- ◯ Respondent
- ◯ Wife/husband
- ◯ Son/daughter
- ◯ Father/mother
- ◯ Grandchild
- ◯ Grandparent
- ◯ Mother/father-in-law
- ◯ Son/daughter-in-law
- ◯ Brother/sister-in-law
- ◯ Paternal aunt/uncle
- ◯ Maternal aunt/uncle
- ◯ Sister/brother
- ◯ Cousin
- ◯ Nephew/niece
- ◯ Step-child/half-brother/sister
- ◯ Co-wife
- ◯ Boyfriend/Girlfriend, including PTM
- ◯ Other not related through blood or marriage (friends)
- ◯ Step-mother/step-father
- ◯ Former wife/husband
- ◯ Former boyfriend/girlfriend, including former PTM
- ◯ NOT part of this family/household
- ◯ Don't know person

HH memb 11: Name of listed person

- ◯ Respondent
- ◯ Wife/husband
- ◯ Son/daughter
- ◯ Father/mother
- ◯ Grandchild
- ◯ Grandparent
- ◯ Mother/father-in-law
- ◯ Son/daughter-in-law
- ◯ Brother/sister-in-law
- ◯ Paternal aunt/uncle
- ◯ Maternal aunt/uncle
- ◯ Sister/brother
- ◯ Cousin
- ◯ Nephew/niece
- ◯ Step-child/half-brother/sister
- ◯ Co-wife
- ◯ Boyfriend/Girlfriend, including PTM
- ◯ Other not related through blood or marriage (friends)
- ◯ Step-mother/step-father
- ◯ Former wife/husband
- ◯ Former boyfriend/girlfriend, including former PTM
- ◯ NOT part of this family/household
- ◯ Don't know person

HH memb 12: Name of listed person

- ◯ Respondent
- ◯ Wife/husband
- ◯ Son/daughter
- ◯ Father/mother
- ◯ Grandchild
- ◯ Grandparent
- ◯ Mother/father-in-law
- ◯ Son/daughter-in-law
- ◯ Brother/sister-in-law
- ◯ Paternal aunt/uncle
- ◯ Maternal aunt/uncle
- ◯ Sister/brother
- ◯ Cousin
- ◯ Nephew/niece
- ◯ Step-child/half-brother/sister
- ◯ Co-wife
- ◯ Boyfriend/Girlfriend, including PTM
- ◯ Other not related through blood or marriage (friends)
- ◯ Step-mother/step-father
- ◯ Former wife/husband
- ◯ Former boyfriend/girlfriend, including former PTM
- ◯ NOT part of this family/household
- ◯ Don't know person

HH memb 13: Name of listed person

- ◯ Respondent
- ◯ Wife/husband
- ◯ Son/daughter
- ◯ Father/mother
- ◯ Grandchild
- ◯ Grandparent
- ◯ Mother/father-in-law
- ◯ Son/daughter-in-law
- ◯ Brother/sister-in-law
- ◯ Paternal aunt/uncle
- ◯ Maternal aunt/uncle
- ◯ Sister/brother
- ◯ Cousin
- ◯ Nephew/niece
- ◯ Step-child/half-brother/sister
- ◯ Co-wife
- ◯ Boyfriend/Girlfriend, including PTM
- ◯ Other not related through blood or marriage (friends)
- ◯ Step-mother/step-father
- ◯ Former wife/husband
- ◯ Former boyfriend/girlfriend, including former PTM
- ◯ NOT part of this family/household
- ◯ Don't know person

HH memb 14: Name of listed person

- ◯ Respondent
- ◯ Wife/husband
- ◯ Son/daughter
- ◯ Father/mother
- ◯ Grandchild
- ◯ Grandparent
- ◯ Mother/father-in-law
- ◯ Son/daughter-in-law
- ◯ Brother/sister-in-law
- ◯ Paternal aunt/uncle
- ◯ Maternal aunt/uncle
- ◯ Sister/brother
- ◯ Cousin
- ◯ Nephew/niece
- ◯ Step-child/half-brother/sister
- ◯ Co-wife
- ◯ Boyfriend/Girlfriend, including PTM
- ◯ Other not related through blood or marriage (friends)
- ◯ Step-mother/step-father
- ◯ Former wife/husband
- ◯ Former boyfriend/girlfriend, including former PTM
- ◯ NOT part of this family/household
- ◯ Don't know person

HH memb 15: Name of listed person

- ◯ Respondent
- ◯ Wife/husband
- ◯ Son/daughter
- ◯ Father/mother
- ◯ Grandchild
- ◯ Grandparent
- ◯ Mother/father-in-law
- ◯ Son/daughter-in-law
- ◯ Brother/sister-in-law
- ◯ Paternal aunt/uncle
- ◯ Maternal aunt/uncle
- ◯ Sister/brother
- ◯ Cousin
- ◯ Nephew/niece
- ◯ Step-child/half-brother/sister
- ◯ Co-wife
- ◯ Boyfriend/Girlfriend, including PTM
- ◯ Other not related through blood or marriage (friends)
- ◯ Step-mother/step-father
- ◯ Former wife/husband
- ◯ Former boyfriend/girlfriend, including former PTM
- ◯ NOT part of this family/household
- ◯ Don't know person

HH memb 16: Name of listed person

- ◯ Respondent
- ◯ Wife/husband
- ◯ Son/daughter
- ◯ Father/mother
- ◯ Grandchild
- ◯ Grandparent
- ◯ Mother/father-in-law
- ◯ Son/daughter-in-law
- ◯ Brother/sister-in-law
- ◯ Paternal aunt/uncle
- ◯ Maternal aunt/uncle
- ◯ Sister/brother
- ◯ Cousin
- ◯ Nephew/niece
- ◯ Step-child/half-brother/sister
- ◯ Co-wife
- ◯ Boyfriend/Girlfriend, including PTM
- ◯ Other not related through blood or marriage (friends)
- ◯ Step-mother/step-father
- ◯ Former wife/husband
- ◯ Former boyfriend/girlfriend, including former PTM
- ◯ NOT part of this family/household
- ◯ Don't know person

HH memb 17: Name of listed person

- ◯ Respondent
- ◯ Wife/husband
- ◯ Son/daughter
- ◯ Father/mother
- ◯ Grandchild
- ◯ Grandparent
- ◯ Mother/father-in-law
- ◯ Son/daughter-in-law
- ◯ Brother/sister-in-law
- ◯ Paternal aunt/uncle
- ◯ Maternal aunt/uncle
- ◯ Sister/brother
- ◯ Cousin
- ◯ Nephew/niece
- ◯ Step-child/half-brother/sister
- ◯ Co-wife
- ◯ Boyfriend/Girlfriend, including PTM
- ◯ Other not related through blood or marriage (friends)
- ◯ Step-mother/step-father
- ◯ Former wife/husband
- ◯ Former boyfriend/girlfriend, including former PTM
- ◯ NOT part of this family/household
- ◯ Don't know person

HH memb 18: Name of listed person

- ◯ Respondent
- ◯ Wife/husband
- ◯ Son/daughter
- ◯ Father/mother
- ◯ Grandchild
- ◯ Grandparent
- ◯ Mother/father-in-law
- ◯ Son/daughter-in-law
- ◯ Brother/sister-in-law
- ◯ Paternal aunt/uncle
- ◯ Maternal aunt/uncle
- ◯ Sister/brother
- ◯ Cousin
- ◯ Nephew/niece
- ◯ Step-child/half-brother/sister
- ◯ Co-wife
- ◯ Boyfriend/Girlfriend, including PTM
- ◯ Other not related through blood or marriage (friends)
- ◯ Step-mother/step-father
- ◯ Former wife/husband
- ◯ Former boyfriend/girlfriend, including former PTM
- ◯ NOT part of this family/household
- ◯ Don't know person

HH memb 19: Name of listed person

- ◯ Respondent
- ◯ Wife/husband
- ◯ Son/daughter
- ◯ Father/mother
- ◯ Grandchild
- ◯ Grandparent
- ◯ Mother/father-in-law
- ◯ Son/daughter-in-law
- ◯ Brother/sister-in-law
- ◯ Paternal aunt/uncle
- ◯ Maternal aunt/uncle
- ◯ Sister/brother
- ◯ Cousin
- ◯ Nephew/niece
- ◯ Step-child/half-brother/sister
- ◯ Co-wife
- ◯ Boyfriend/Girlfriend, including PTM
- ◯ Other not related through blood or marriage (friends)
- ◯ Step-mother/step-father
- ◯ Former wife/husband
- ◯ Former boyfriend/girlfriend, including former PTM
- ◯ NOT part of this family/household
- ◯ Don't know person

HH memb 20: Name of listed person

- ◯ Respondent
- ◯ Wife/husband
- ◯ Son/daughter
- ◯ Father/mother
- ◯ Grandchild
- ◯ Grandparent
- ◯ Mother/father-in-law
- ◯ Son/daughter-in-law
- ◯ Brother/sister-in-law
- ◯ Paternal aunt/uncle
- ◯ Maternal aunt/uncle
- ◯ Sister/brother
- ◯ Cousin
- ◯ Nephew/niece
- ◯ Step-child/half-brother/sister
- ◯ Co-wife
- ◯ Boyfriend/Girlfriend, including PTM
- ◯ Other not related through blood or marriage (friends)
- ◯ Step-mother/step-father
- ◯ Former wife/husband
- ◯ Former boyfriend/girlfriend, including former PTM
- ◯ NOT part of this family/household
- ◯ Don't know person

HH memb 21: Name of listed person

- ◯ Respondent
- ◯ Wife/husband
- ◯ Son/daughter
- ◯ Father/mother
- ◯ Grandchild
- ◯ Grandparent
- ◯ Mother/father-in-law
- ◯ Son/daughter-in-law
- ◯ Brother/sister-in-law
- ◯ Paternal aunt/uncle
- ◯ Maternal aunt/uncle
- ◯ Sister/brother
- ◯ Cousin
- ◯ Nephew/niece
- ◯ Step-child/half-brother/sister
- ◯ Co-wife
- ◯ Boyfriend/Girlfriend, including PTM
- ◯ Other not related through blood or marriage (friends)
- ◯ Step-mother/step-father
- ◯ Former wife/husband
- ◯ Former boyfriend/girlfriend, including former PTM
- ◯ NOT part of this family/household
- ◯ Don't know person

HH memb 22: Name of listed person

- ◯ Respondent
- ◯ Wife/husband
- ◯ Son/daughter
- ◯ Father/mother
- ◯ Grandchild
- ◯ Grandparent
- ◯ Mother/father-in-law
- ◯ Son/daughter-in-law
- ◯ Brother/sister-in-law
- ◯ Paternal aunt/uncle
- ◯ Maternal aunt/uncle
- ◯ Sister/brother
- ◯ Cousin
- ◯ Nephew/niece
- ◯ Step-child/half-brother/sister
- ◯ Co-wife
- ◯ Boyfriend/Girlfriend, including PTM
- ◯ Other not related through blood or marriage (friends)
- ◯ Step-mother/step-father
- ◯ Former wife/husband
- ◯ Former boyfriend/girlfriend, including former PTM
- ◯ NOT part of this family/household
- ◯ Don't know person

HH memb 1: Age of listed person

- ◯ < 1 years old
- ◯ now about 1 year old
- ◯ now about 2 year old
- ◯ now about 3 year old
- ◯ now about 4 year old
- ◯ now about 5 year old
- ◯ now about 6 year old
- ◯ now about 7 year old
- ◯ now about 8 year old
- ◯ now about 9 year old
- ◯ now about 10 year old
- ◯ now about 11 year old
- ◯ now about 12 year old
- ◯ now about 13 year old
- ◯ now about 14 year old
- ◯ now about 15 year old
- ◯ now about 16 year old
- ◯ now about 17 year old
- ◯ now about 18 year old
- ◯ now about 19 year old
- ◯ now about 20 year old
- ◯ now about 21 year old
- ◯ now about 22 year old
- ◯ now about 23 year old
- ◯ now about 24 year old
- ◯ now about 25 year old
- ◯ now about 26 year old
- ◯ now about 27 year old
- ◯ now about 28 year old
- ◯ now about 29 year old
- ◯ now about 30 year old
- ◯ now about 31 year old
- ◯ now about 32 year old
- ◯ now about 33 year old
- ◯ now about 34 year old
- ◯ now about 35 year old
- ◯ now about 36 year old
- ◯ now about 37 year old
- ◯ now about 38 year old
- ◯ now about 39 year old
- ◯ now about 40 year old
- ◯ now about 41 year old
- ◯ now about 42 year old
- ◯ now about 43 year old
- ◯ now about 44 year old
- ◯ now about 45 year old
- ◯ now about 46 year old
- ◯ now about 47 year old
- ◯ now about 48 year old
- ◯ now about 49 year old
- ◯ now about 50 year old
- ◯ now about 51 year old
- ◯ now about 52 year old
- ◯ now about 53 year old
- ◯ now about 54 year old
- ◯ now about 55 year old
- ◯ now about 56 year old
- ◯ now about 57 year old
- ◯ now about 58 year old
- ◯ now about 59 year old
- ◯ now about 60 year old
- ◯ now about 61 year old
- ◯ now about 62 year old
- ◯ now about 63 year old
- ◯ now about 64 year old
- ◯ now about 65 year old
- ◯ now about 66 year old
- ◯ now about 67 year old
- ◯ now about 68 year old

◯ now about 69 year old
◯ now about 70 year old
◯ now about 71 year old
◯ now about 72 year old
◯ now about 73 year old
◯ now about 74 year old
◯ now about 75 year old
◯ now about 76 year old
◯ now about 77 year old
◯ now about 78 year old
◯ now about 79 year old
◯ now about 80 year old
◯ now about 81 year old
◯ now about 82 year old
◯ now about 83 year old
◯ now about 84 year old
◯ now about 85 year old
◯ now about 86 year old
◯ now about 87 year old
◯ now about 88 year old
◯ now about 89 year old
◯ now about 90 years or older

HH memb 2: Age of listed person

◯ < 1 years old
◯ now about 1 year old
◯ now about 2 year old
◯ now about 3 year old
◯ now about 4 year old
◯ now about 5 year old
◯ now about 6 year old
◯ now about 7 year old
◯ now about 8 year old
◯ now about 9 year old
◯ now about 10 year old
◯ now about 11 year old
◯ now about 12 year old
◯ now about 13 year old
◯ now about 14 year old
◯ now about 15 year old
◯ now about 16 year old
◯ now about 17 year old
◯ now about 18 year old
◯ now about 19 year old
◯ now about 20 year old
◯ now about 21 year old
◯ now about 22 year old
◯ now about 23 year old
◯ now about 24 year old
◯ now about 25 year old
◯ now about 26 year old
◯ now about 27 year old
◯ now about 28 year old
◯ now about 29 year old
◯ now about 30 year old
◯ now about 31 year old
◯ now about 32 year old
◯ now about 33 year old
◯ now about 34 year old
◯ now about 35 year old
◯ now about 36 year old
◯ now about 37 year old
◯ now about 38 year old
◯ now about 39 year old
◯ now about 40 year old
◯ now about 41 year old
◯ now about 42 year old
◯ now about 43 year old
◯ now about 44 year old
◯ now about 45 year old
◯ now about 46 year old
◯ now about 47 year old
◯ now about 48 year old
◯ now about 49 year old
◯ now about 50 year old
◯ now about 51 year old
◯ now about 52 year old
◯ now about 53 year old
◯ now about 54 year old
◯ now about 55 year old
◯ now about 56 year old
◯ now about 57 year old
◯ now about 58 year old
◯ now about 59 year old
◯ now about 60 year old
◯ now about 61 year old
◯ now about 62 year old
◯ now about 63 year old
◯ now about 64 year old
◯ now about 65 year old
◯ now about 66 year old
◯ now about 67 year old
◯ now about 68 year old

◯ now about 69 year old
◯ now about 70 year old
◯ now about 71 year old
◯ now about 72 year old
◯ now about 73 year old
◯ now about 74 year old
◯ now about 75 year old
◯ now about 76 year old
◯ now about 77 year old
◯ now about 78 year old
◯ now about 79 year old
◯ now about 80 year old
◯ now about 81 year old
◯ now about 82 year old
◯ now about 83 year old
◯ now about 84 year old
◯ now about 85 year old
◯ now about 86 year old
◯ now about 87 year old
◯ now about 88 year old
◯ now about 89 year old
◯ now about 90 years or older

HH memb 3: Age of listed person

- ◯ < 1 years old
- ◯ now about 1 year old
- ◯ now about 2 year old
- ◯ now about 3 year old
- ◯ now about 4 year old
- ◯ now about 5 year old
- ◯ now about 6 year old
- ◯ now about 7 year old
- ◯ now about 8 year old
- ◯ now about 9 year old
- ◯ now about 10 year old
- ◯ now about 11 year old
- ◯ now about 12 year old
- ◯ now about 13 year old
- ◯ now about 14 year old
- ◯ now about 15 year old
- ◯ now about 16 year old
- ◯ now about 17 year old
- ◯ now about 18 year old
- ◯ now about 19 year old
- ◯ now about 20 year old
- ◯ now about 21 year old
- ◯ now about 22 year old
- ◯ now about 23 year old
- ◯ now about 24 year old
- ◯ now about 25 year old
- ◯ now about 26 year old
- ◯ now about 27 year old
- ◯ now about 28 year old
- ◯ now about 29 year old
- ◯ now about 30 year old
- ◯ now about 31 year old
- ◯ now about 32 year old
- ◯ now about 33 year old
- ◯ now about 34 year old
- ◯ now about 35 year old
- ◯ now about 36 year old
- ◯ now about 37 year old
- ◯ now about 38 year old
- ◯ now about 39 year old
- ◯ now about 40 year old
- ◯ now about 41 year old
- ◯ now about 42 year old
- ◯ now about 43 year old
- ◯ now about 44 year old
- ◯ now about 45 year old
- ◯ now about 46 year old
- ◯ now about 47 year old
- ◯ now about 48 year old
- ◯ now about 49 year old
- ◯ now about 50 year old
- ◯ now about 51 year old
- ◯ now about 52 year old
- ◯ now about 53 year old
- ◯ now about 54 year old
- ◯ now about 55 year old
- ◯ now about 56 year old
- ◯ now about 57 year old
- ◯ now about 58 year old
- ◯ now about 59 year old
- ◯ now about 60 year old
- ◯ now about 61 year old
- ◯ now about 62 year old
- ◯ now about 63 year old
- ◯ now about 64 year old
- ◯ now about 65 year old
- ◯ now about 66 year old
- ◯ now about 67 year old
- ◯ now about 68 year old

◯ now about 69 year old
◯ now about 70 year old
◯ now about 71 year old
◯ now about 72 year old
◯ now about 73 year old
◯ now about 74 year old
◯ now about 75 year old
◯ now about 76 year old
◯ now about 77 year old
◯ now about 78 year old
◯ now about 79 year old
◯ now about 80 year old
◯ now about 81 year old
◯ now about 82 year old
◯ now about 83 year old
◯ now about 84 year old
◯ now about 85 year old
◯ now about 86 year old
◯ now about 87 year old
◯ now about 88 year old
◯ now about 89 year old
◯ now about 90 years or older

HH memb 4: Age of listed person

- ◯ < 1 years old
- ◯ now about 1 year old
- ◯ now about 2 year old
- ◯ now about 3 year old
- ◯ now about 4 year old
- ◯ now about 5 year old
- ◯ now about 6 year old
- ◯ now about 7 year old
- ◯ now about 8 year old
- ◯ now about 9 year old
- ◯ now about 10 year old
- ◯ now about 11 year old
- ◯ now about 12 year old
- ◯ now about 13 year old
- ◯ now about 14 year old
- ◯ now about 15 year old
- ◯ now about 16 year old
- ◯ now about 17 year old
- ◯ now about 18 year old
- ◯ now about 19 year old
- ◯ now about 20 year old
- ◯ now about 21 year old
- ◯ now about 22 year old
- ◯ now about 23 year old
- ◯ now about 24 year old
- ◯ now about 25 year old
- ◯ now about 26 year old
- ◯ now about 27 year old
- ◯ now about 28 year old
- ◯ now about 29 year old
- ◯ now about 30 year old
- ◯ now about 31 year old
- ◯ now about 32 year old
- ◯ now about 33 year old
- ◯ now about 34 year old
- ◯ now about 35 year old
- ◯ now about 36 year old
- ◯ now about 37 year old
- ◯ now about 38 year old
- ◯ now about 39 year old
- ◯ now about 40 year old
- ◯ now about 41 year old
- ◯ now about 42 year old
- ◯ now about 43 year old
- ◯ now about 44 year old
- ◯ now about 45 year old
- ◯ now about 46 year old
- ◯ now about 47 year old
- ◯ now about 48 year old
- ◯ now about 49 year old
- ◯ now about 50 year old
- ◯ now about 51 year old
- ◯ now about 52 year old
- ◯ now about 53 year old
- ◯ now about 54 year old
- ◯ now about 55 year old
- ◯ now about 56 year old
- ◯ now about 57 year old
- ◯ now about 58 year old
- ◯ now about 59 year old
- ◯ now about 60 year old
- ◯ now about 61 year old
- ◯ now about 62 year old
- ◯ now about 63 year old
- ◯ now about 64 year old
- ◯ now about 65 year old
- ◯ now about 66 year old
- ◯ now about 67 year old
- ◯ now about 68 year old

◯ now about 69 year old
◯ now about 70 year old
◯ now about 71 year old
◯ now about 72 year old
◯ now about 73 year old
◯ now about 74 year old
◯ now about 75 year old
◯ now about 76 year old
◯ now about 77 year old
◯ now about 78 year old
◯ now about 79 year old
◯ now about 80 year old
◯ now about 81 year old
◯ now about 82 year old
◯ now about 83 year old
◯ now about 84 year old
◯ now about 85 year old
◯ now about 86 year old
◯ now about 87 year old
◯ now about 88 year old
◯ now about 89 year old
◯ now about 90 years or older

HH memb 5: Age of listed person

- ◯ < 1 years old
- ◯ now about 1 year old
- ◯ now about 2 year old
- ◯ now about 3 year old
- ◯ now about 4 year old
- ◯ now about 5 year old
- ◯ now about 6 year old
- ◯ now about 7 year old
- ◯ now about 8 year old
- ◯ now about 9 year old
- ◯ now about 10 year old
- ◯ now about 11 year old
- ◯ now about 12 year old
- ◯ now about 13 year old
- ◯ now about 14 year old
- ◯ now about 15 year old
- ◯ now about 16 year old
- ◯ now about 17 year old
- ◯ now about 18 year old
- ◯ now about 19 year old
- ◯ now about 20 year old
- ◯ now about 21 year old
- ◯ now about 22 year old
- ◯ now about 23 year old
- ◯ now about 24 year old
- ◯ now about 25 year old
- ◯ now about 26 year old
- ◯ now about 27 year old
- ◯ now about 28 year old
- ◯ now about 29 year old
- ◯ now about 30 year old
- ◯ now about 31 year old
- ◯ now about 32 year old
- ◯ now about 33 year old
- ◯ now about 34 year old
- ◯ now about 35 year old
- ◯ now about 36 year old
- ◯ now about 37 year old
- ◯ now about 38 year old
- ◯ now about 39 year old
- ◯ now about 40 year old
- ◯ now about 41 year old
- ◯ now about 42 year old
- ◯ now about 43 year old
- ◯ now about 44 year old
- ◯ now about 45 year old
- ◯ now about 46 year old
- ◯ now about 47 year old
- ◯ now about 48 year old
- ◯ now about 49 year old
- ◯ now about 50 year old
- ◯ now about 51 year old
- ◯ now about 52 year old
- ◯ now about 53 year old
- ◯ now about 54 year old
- ◯ now about 55 year old
- ◯ now about 56 year old
- ◯ now about 57 year old
- ◯ now about 58 year old
- ◯ now about 59 year old
- ◯ now about 60 year old
- ◯ now about 61 year old
- ◯ now about 62 year old
- ◯ now about 63 year old
- ◯ now about 64 year old
- ◯ now about 65 year old
- ◯ now about 66 year old
- ◯ now about 67 year old
- ◯ now about 68 year old

◯ now about 69 year old
◯ now about 70 year old
◯ now about 71 year old
◯ now about 72 year old
◯ now about 73 year old
◯ now about 74 year old
◯ now about 75 year old
◯ now about 76 year old
◯ now about 77 year old
◯ now about 78 year old
◯ now about 79 year old
◯ now about 80 year old
◯ now about 81 year old
◯ now about 82 year old
◯ now about 83 year old
◯ now about 84 year old
◯ now about 85 year old
◯ now about 86 year old
◯ now about 87 year old
◯ now about 88 year old
◯ now about 89 year old
◯ now about 90 years or older

HH memb 6: Age of listed person

- < 1 years old
- now about 1 year old
- now about 2 year old
- now about 3 year old
- now about 4 year old
- now about 5 year old
- now about 6 year old
- now about 7 year old
- now about 8 year old
- now about 9 year old
- now about 10 year old
- now about 11 year old
- now about 12 year old
- now about 13 year old
- now about 14 year old
- now about 15 year old
- now about 16 year old
- now about 17 year old
- now about 18 year old
- now about 19 year old
- now about 20 year old
- now about 21 year old
- now about 22 year old
- now about 23 year old
- now about 24 year old
- now about 25 year old
- now about 26 year old
- now about 27 year old
- now about 28 year old
- now about 29 year old
- now about 30 year old
- now about 31 year old
- now about 32 year old
- now about 33 year old
- now about 34 year old
- now about 35 year old
- now about 36 year old
- now about 37 year old
- now about 38 year old
- now about 39 year old
- now about 40 year old
- now about 41 year old
- now about 42 year old
- now about 43 year old
- now about 44 year old
- now about 45 year old
- now about 46 year old
- now about 47 year old
- now about 48 year old
- now about 49 year old
- now about 50 year old
- now about 51 year old
- now about 52 year old
- now about 53 year old
- now about 54 year old
- now about 55 year old
- now about 56 year old
- now about 57 year old
- now about 58 year old
- now about 59 year old
- now about 60 year old
- now about 61 year old
- now about 62 year old
- now about 63 year old
- now about 64 year old
- now about 65 year old
- now about 66 year old
- now about 67 year old
- now about 68 year old

◯ now about 69 year old
◯ now about 70 year old
◯ now about 71 year old
◯ now about 72 year old
◯ now about 73 year old
◯ now about 74 year old
◯ now about 75 year old
◯ now about 76 year old
◯ now about 77 year old
◯ now about 78 year old
◯ now about 79 year old
◯ now about 80 year old
◯ now about 81 year old
◯ now about 82 year old
◯ now about 83 year old
◯ now about 84 year old
◯ now about 85 year old
◯ now about 86 year old
◯ now about 87 year old
◯ now about 88 year old
◯ now about 89 year old
◯ now about 90 years or older

HH memb 7: Age of listed person

- ◯ < 1 years old
- ◯ now about 1 year old
- ◯ now about 2 year old
- ◯ now about 3 year old
- ◯ now about 4 year old
- ◯ now about 5 year old
- ◯ now about 6 year old
- ◯ now about 7 year old
- ◯ now about 8 year old
- ◯ now about 9 year old
- ◯ now about 10 year old
- ◯ now about 11 year old
- ◯ now about 12 year old
- ◯ now about 13 year old
- ◯ now about 14 year old
- ◯ now about 15 year old
- ◯ now about 16 year old
- ◯ now about 17 year old
- ◯ now about 18 year old
- ◯ now about 19 year old
- ◯ now about 20 year old
- ◯ now about 21 year old
- ◯ now about 22 year old
- ◯ now about 23 year old
- ◯ now about 24 year old
- ◯ now about 25 year old
- ◯ now about 26 year old
- ◯ now about 27 year old
- ◯ now about 28 year old
- ◯ now about 29 year old
- ◯ now about 30 year old
- ◯ now about 31 year old
- ◯ now about 32 year old
- ◯ now about 33 year old
- ◯ now about 34 year old
- ◯ now about 35 year old
- ◯ now about 36 year old
- ◯ now about 37 year old
- ◯ now about 38 year old
- ◯ now about 39 year old
- ◯ now about 40 year old
- ◯ now about 41 year old
- ◯ now about 42 year old
- ◯ now about 43 year old
- ◯ now about 44 year old
- ◯ now about 45 year old
- ◯ now about 46 year old
- ◯ now about 47 year old
- ◯ now about 48 year old
- ◯ now about 49 year old
- ◯ now about 50 year old
- ◯ now about 51 year old
- ◯ now about 52 year old
- ◯ now about 53 year old
- ◯ now about 54 year old
- ◯ now about 55 year old
- ◯ now about 56 year old
- ◯ now about 57 year old
- ◯ now about 58 year old
- ◯ now about 59 year old
- ◯ now about 60 year old
- ◯ now about 61 year old
- ◯ now about 62 year old
- ◯ now about 63 year old
- ◯ now about 64 year old
- ◯ now about 65 year old
- ◯ now about 66 year old
- ◯ now about 67 year old
- ◯ now about 68 year old

◯ now about 69 year old
◯ now about 70 year old
◯ now about 71 year old
◯ now about 72 year old
◯ now about 73 year old
◯ now about 74 year old
◯ now about 75 year old
◯ now about 76 year old
◯ now about 77 year old
◯ now about 78 year old
◯ now about 79 year old
◯ now about 80 year old
◯ now about 81 year old
◯ now about 82 year old
◯ now about 83 year old
◯ now about 84 year old
◯ now about 85 year old
◯ now about 86 year old
◯ now about 87 year old
◯ now about 88 year old
◯ now about 89 year old
◯ now about 90 years or older

HH memb 8: Age of listed person

- ◯ < 1 years old
- ◯ now about 1 year old
- ◯ now about 2 year old
- ◯ now about 3 year old
- ◯ now about 4 year old
- ◯ now about 5 year old
- ◯ now about 6 year old
- ◯ now about 7 year old
- ◯ now about 8 year old
- ◯ now about 9 year old
- ◯ now about 10 year old
- ◯ now about 11 year old
- ◯ now about 12 year old
- ◯ now about 13 year old
- ◯ now about 14 year old
- ◯ now about 15 year old
- ◯ now about 16 year old
- ◯ now about 17 year old
- ◯ now about 18 year old
- ◯ now about 19 year old
- ◯ now about 20 year old
- ◯ now about 21 year old
- ◯ now about 22 year old
- ◯ now about 23 year old
- ◯ now about 24 year old
- ◯ now about 25 year old
- ◯ now about 26 year old
- ◯ now about 27 year old
- ◯ now about 28 year old
- ◯ now about 29 year old
- ◯ now about 30 year old
- ◯ now about 31 year old
- ◯ now about 32 year old
- ◯ now about 33 year old
- ◯ now about 34 year old
- ◯ now about 35 year old
- ◯ now about 36 year old
- ◯ now about 37 year old
- ◯ now about 38 year old
- ◯ now about 39 year old
- ◯ now about 40 year old
- ◯ now about 41 year old
- ◯ now about 42 year old
- ◯ now about 43 year old
- ◯ now about 44 year old
- ◯ now about 45 year old
- ◯ now about 46 year old
- ◯ now about 47 year old
- ◯ now about 48 year old
- ◯ now about 49 year old
- ◯ now about 50 year old
- ◯ now about 51 year old
- ◯ now about 52 year old
- ◯ now about 53 year old
- ◯ now about 54 year old
- ◯ now about 55 year old
- ◯ now about 56 year old
- ◯ now about 57 year old
- ◯ now about 58 year old
- ◯ now about 59 year old
- ◯ now about 60 year old
- ◯ now about 61 year old
- ◯ now about 62 year old
- ◯ now about 63 year old
- ◯ now about 64 year old
- ◯ now about 65 year old
- ◯ now about 66 year old
- ◯ now about 67 year old
- ◯ now about 68 year old

◯ now about 69 year old
◯ now about 70 year old
◯ now about 71 year old
◯ now about 72 year old
◯ now about 73 year old
◯ now about 74 year old
◯ now about 75 year old
◯ now about 76 year old
◯ now about 77 year old
◯ now about 78 year old
◯ now about 79 year old
◯ now about 80 year old
◯ now about 81 year old
◯ now about 82 year old
◯ now about 83 year old
◯ now about 84 year old
◯ now about 85 year old
◯ now about 86 year old
◯ now about 87 year old
◯ now about 88 year old
◯ now about 89 year old
◯ now about 90 years or older

HH memb 9: Age of listed person

- < 1 years old
- now about 1 year old
- now about 2 year old
- now about 3 year old
- now about 4 year old
- now about 5 year old
- now about 6 year old
- now about 7 year old
- now about 8 year old
- now about 9 year old
- now about 10 year old
- now about 11 year old
- now about 12 year old
- now about 13 year old
- now about 14 year old
- now about 15 year old
- now about 16 year old
- now about 17 year old
- now about 18 year old
- now about 19 year old
- now about 20 year old
- now about 21 year old
- now about 22 year old
- now about 23 year old
- now about 24 year old
- now about 25 year old
- now about 26 year old
- now about 27 year old
- now about 28 year old
- now about 29 year old
- now about 30 year old
- now about 31 year old
- now about 32 year old
- now about 33 year old
- now about 34 year old
- now about 35 year old
- now about 36 year old
- now about 37 year old
- now about 38 year old
- now about 39 year old
- now about 40 year old
- now about 41 year old
- now about 42 year old
- now about 43 year old
- now about 44 year old
- now about 45 year old
- now about 46 year old
- now about 47 year old
- now about 48 year old
- now about 49 year old
- now about 50 year old
- now about 51 year old
- now about 52 year old
- now about 53 year old
- now about 54 year old
- now about 55 year old
- now about 56 year old
- now about 57 year old
- now about 58 year old
- now about 59 year old
- now about 60 year old
- now about 61 year old
- now about 62 year old
- now about 63 year old
- now about 64 year old
- now about 65 year old
- now about 66 year old
- now about 67 year old
- now about 68 year old

◯ now about 69 year old
◯ now about 70 year old
◯ now about 71 year old
◯ now about 72 year old
◯ now about 73 year old
◯ now about 74 year old
◯ now about 75 year old
◯ now about 76 year old
◯ now about 77 year old
◯ now about 78 year old
◯ now about 79 year old
◯ now about 80 year old
◯ now about 81 year old
◯ now about 82 year old
◯ now about 83 year old
◯ now about 84 year old
◯ now about 85 year old
◯ now about 86 year old
◯ now about 87 year old
◯ now about 88 year old
◯ now about 89 year old
◯ now about 90 years or older

HH memb 10: Age of listed person

- ◯ < 1 years old
- ◯ now about 1 year old
- ◯ now about 2 year old
- ◯ now about 3 year old
- ◯ now about 4 year old
- ◯ now about 5 year old
- ◯ now about 6 year old
- ◯ now about 7 year old
- ◯ now about 8 year old
- ◯ now about 9 year old
- ◯ now about 10 year old
- ◯ now about 11 year old
- ◯ now about 12 year old
- ◯ now about 13 year old
- ◯ now about 14 year old
- ◯ now about 15 year old
- ◯ now about 16 year old
- ◯ now about 17 year old
- ◯ now about 18 year old
- ◯ now about 19 year old
- ◯ now about 20 year old
- ◯ now about 21 year old
- ◯ now about 22 year old
- ◯ now about 23 year old
- ◯ now about 24 year old
- ◯ now about 25 year old
- ◯ now about 26 year old
- ◯ now about 27 year old
- ◯ now about 28 year old
- ◯ now about 29 year old
- ◯ now about 30 year old
- ◯ now about 31 year old
- ◯ now about 32 year old
- ◯ now about 33 year old
- ◯ now about 34 year old
- ◯ now about 35 year old
- ◯ now about 36 year old
- ◯ now about 37 year old
- ◯ now about 38 year old
- ◯ now about 39 year old
- ◯ now about 40 year old
- ◯ now about 41 year old
- ◯ now about 42 year old
- ◯ now about 43 year old
- ◯ now about 44 year old
- ◯ now about 45 year old
- ◯ now about 46 year old
- ◯ now about 47 year old
- ◯ now about 48 year old
- ◯ now about 49 year old
- ◯ now about 50 year old
- ◯ now about 51 year old
- ◯ now about 52 year old
- ◯ now about 53 year old
- ◯ now about 54 year old
- ◯ now about 55 year old
- ◯ now about 56 year old
- ◯ now about 57 year old
- ◯ now about 58 year old
- ◯ now about 59 year old
- ◯ now about 60 year old
- ◯ now about 61 year old
- ◯ now about 62 year old
- ◯ now about 63 year old
- ◯ now about 64 year old
- ◯ now about 65 year old
- ◯ now about 66 year old
- ◯ now about 67 year old
- ◯ now about 68 year old

◯ now about 69 year old
◯ now about 70 year old
◯ now about 71 year old
◯ now about 72 year old
◯ now about 73 year old
◯ now about 74 year old
◯ now about 75 year old
◯ now about 76 year old
◯ now about 77 year old
◯ now about 78 year old
◯ now about 79 year old
◯ now about 80 year old
◯ now about 81 year old
◯ now about 82 year old
◯ now about 83 year old
◯ now about 84 year old
◯ now about 85 year old
◯ now about 86 year old
◯ now about 87 year old
◯ now about 88 year old
◯ now about 89 year old
◯ now about 90 years or older

HH memb 11: Age of listed person

- ◯ < 1 years old
- ◯ now about 1 year old
- ◯ now about 2 year old
- ◯ now about 3 year old
- ◯ now about 4 year old
- ◯ now about 5 year old
- ◯ now about 6 year old
- ◯ now about 7 year old
- ◯ now about 8 year old
- ◯ now about 9 year old
- ◯ now about 10 year old
- ◯ now about 11 year old
- ◯ now about 12 year old
- ◯ now about 13 year old
- ◯ now about 14 year old
- ◯ now about 15 year old
- ◯ now about 16 year old
- ◯ now about 17 year old
- ◯ now about 18 year old
- ◯ now about 19 year old
- ◯ now about 20 year old
- ◯ now about 21 year old
- ◯ now about 22 year old
- ◯ now about 23 year old
- ◯ now about 24 year old
- ◯ now about 25 year old
- ◯ now about 26 year old
- ◯ now about 27 year old
- ◯ now about 28 year old
- ◯ now about 29 year old
- ◯ now about 30 year old
- ◯ now about 31 year old
- ◯ now about 32 year old
- ◯ now about 33 year old
- ◯ now about 34 year old
- ◯ now about 35 year old
- ◯ now about 36 year old
- ◯ now about 37 year old
- ◯ now about 38 year old
- ◯ now about 39 year old
- ◯ now about 40 year old
- ◯ now about 41 year old
- ◯ now about 42 year old
- ◯ now about 43 year old
- ◯ now about 44 year old
- ◯ now about 45 year old
- ◯ now about 46 year old
- ◯ now about 47 year old
- ◯ now about 48 year old
- ◯ now about 49 year old
- ◯ now about 50 year old
- ◯ now about 51 year old
- ◯ now about 52 year old
- ◯ now about 53 year old
- ◯ now about 54 year old
- ◯ now about 55 year old
- ◯ now about 56 year old
- ◯ now about 57 year old
- ◯ now about 58 year old
- ◯ now about 59 year old
- ◯ now about 60 year old
- ◯ now about 61 year old
- ◯ now about 62 year old
- ◯ now about 63 year old
- ◯ now about 64 year old
- ◯ now about 65 year old
- ◯ now about 66 year old
- ◯ now about 67 year old
- ◯ now about 68 year old

◯ now about 69 year old
◯ now about 70 year old
◯ now about 71 year old
◯ now about 72 year old
◯ now about 73 year old
◯ now about 74 year old
◯ now about 75 year old
◯ now about 76 year old
◯ now about 77 year old
◯ now about 78 year old
◯ now about 79 year old
◯ now about 80 year old
◯ now about 81 year old
◯ now about 82 year old
◯ now about 83 year old
◯ now about 84 year old
◯ now about 85 year old
◯ now about 86 year old
◯ now about 87 year old
◯ now about 88 year old
◯ now about 89 year old
◯ now about 90 years or older

HH memb 12: Age of listed person

- ◯ < 1 years old
- ◯ now about 1 year old
- ◯ now about 2 year old
- ◯ now about 3 year old
- ◯ now about 4 year old
- ◯ now about 5 year old
- ◯ now about 6 year old
- ◯ now about 7 year old
- ◯ now about 8 year old
- ◯ now about 9 year old
- ◯ now about 10 year old
- ◯ now about 11 year old
- ◯ now about 12 year old
- ◯ now about 13 year old
- ◯ now about 14 year old
- ◯ now about 15 year old
- ◯ now about 16 year old
- ◯ now about 17 year old
- ◯ now about 18 year old
- ◯ now about 19 year old
- ◯ now about 20 year old
- ◯ now about 21 year old
- ◯ now about 22 year old
- ◯ now about 23 year old
- ◯ now about 24 year old
- ◯ now about 25 year old
- ◯ now about 26 year old
- ◯ now about 27 year old
- ◯ now about 28 year old
- ◯ now about 29 year old
- ◯ now about 30 year old
- ◯ now about 31 year old
- ◯ now about 32 year old
- ◯ now about 33 year old
- ◯ now about 34 year old
- ◯ now about 35 year old
- ◯ now about 36 year old
- ◯ now about 37 year old
- ◯ now about 38 year old
- ◯ now about 39 year old
- ◯ now about 40 year old
- ◯ now about 41 year old
- ◯ now about 42 year old
- ◯ now about 43 year old
- ◯ now about 44 year old
- ◯ now about 45 year old
- ◯ now about 46 year old
- ◯ now about 47 year old
- ◯ now about 48 year old
- ◯ now about 49 year old
- ◯ now about 50 year old
- ◯ now about 51 year old
- ◯ now about 52 year old
- ◯ now about 53 year old
- ◯ now about 54 year old
- ◯ now about 55 year old
- ◯ now about 56 year old
- ◯ now about 57 year old
- ◯ now about 58 year old
- ◯ now about 59 year old
- ◯ now about 60 year old
- ◯ now about 61 year old
- ◯ now about 62 year old
- ◯ now about 63 year old
- ◯ now about 64 year old
- ◯ now about 65 year old
- ◯ now about 66 year old
- ◯ now about 67 year old
- ◯ now about 68 year old

◯ now about 69 year old
◯ now about 70 year old
◯ now about 71 year old
◯ now about 72 year old
◯ now about 73 year old
◯ now about 74 year old
◯ now about 75 year old
◯ now about 76 year old
◯ now about 77 year old
◯ now about 78 year old
◯ now about 79 year old
◯ now about 80 year old
◯ now about 81 year old
◯ now about 82 year old
◯ now about 83 year old
◯ now about 84 year old
◯ now about 85 year old
◯ now about 86 year old
◯ now about 87 year old
◯ now about 88 year old
◯ now about 89 year old
◯ now about 90 years or older

HH memb 13: Age of listed person

- < 1 years old
- now about 1 year old
- now about 2 year old
- now about 3 year old
- now about 4 year old
- now about 5 year old
- now about 6 year old
- now about 7 year old
- now about 8 year old
- now about 9 year old
- now about 10 year old
- now about 11 year old
- now about 12 year old
- now about 13 year old
- now about 14 year old
- now about 15 year old
- now about 16 year old
- now about 17 year old
- now about 18 year old
- now about 19 year old
- now about 20 year old
- now about 21 year old
- now about 22 year old
- now about 23 year old
- now about 24 year old
- now about 25 year old
- now about 26 year old
- now about 27 year old
- now about 28 year old
- now about 29 year old
- now about 30 year old
- now about 31 year old
- now about 32 year old
- now about 33 year old
- now about 34 year old
- now about 35 year old
- now about 36 year old
- now about 37 year old
- now about 38 year old
- now about 39 year old
- now about 40 year old
- now about 41 year old
- now about 42 year old
- now about 43 year old
- now about 44 year old
- now about 45 year old
- now about 46 year old
- now about 47 year old
- now about 48 year old
- now about 49 year old
- now about 50 year old
- now about 51 year old
- now about 52 year old
- now about 53 year old
- now about 54 year old
- now about 55 year old
- now about 56 year old
- now about 57 year old
- now about 58 year old
- now about 59 year old
- now about 60 year old
- now about 61 year old
- now about 62 year old
- now about 63 year old
- now about 64 year old
- now about 65 year old
- now about 66 year old
- now about 67 year old
- now about 68 year old

◯ now about 69 year old
◯ now about 70 year old
◯ now about 71 year old
◯ now about 72 year old
◯ now about 73 year old
◯ now about 74 year old
◯ now about 75 year old
◯ now about 76 year old
◯ now about 77 year old
◯ now about 78 year old
◯ now about 79 year old
◯ now about 80 year old
◯ now about 81 year old
◯ now about 82 year old
◯ now about 83 year old
◯ now about 84 year old
◯ now about 85 year old
◯ now about 86 year old
◯ now about 87 year old
◯ now about 88 year old
◯ now about 89 year old
◯ now about 90 years or older

HH memb 14: Age of listed person

- ◯ < 1 years old
- ◯ now about 1 year old
- ◯ now about 2 year old
- ◯ now about 3 year old
- ◯ now about 4 year old
- ◯ now about 5 year old
- ◯ now about 6 year old
- ◯ now about 7 year old
- ◯ now about 8 year old
- ◯ now about 9 year old
- ◯ now about 10 year old
- ◯ now about 11 year old
- ◯ now about 12 year old
- ◯ now about 13 year old
- ◯ now about 14 year old
- ◯ now about 15 year old
- ◯ now about 16 year old
- ◯ now about 17 year old
- ◯ now about 18 year old
- ◯ now about 19 year old
- ◯ now about 20 year old
- ◯ now about 21 year old
- ◯ now about 22 year old
- ◯ now about 23 year old
- ◯ now about 24 year old
- ◯ now about 25 year old
- ◯ now about 26 year old
- ◯ now about 27 year old
- ◯ now about 28 year old
- ◯ now about 29 year old
- ◯ now about 30 year old
- ◯ now about 31 year old
- ◯ now about 32 year old
- ◯ now about 33 year old
- ◯ now about 34 year old
- ◯ now about 35 year old
- ◯ now about 36 year old
- ◯ now about 37 year old
- ◯ now about 38 year old
- ◯ now about 39 year old
- ◯ now about 40 year old
- ◯ now about 41 year old
- ◯ now about 42 year old
- ◯ now about 43 year old
- ◯ now about 44 year old
- ◯ now about 45 year old
- ◯ now about 46 year old
- ◯ now about 47 year old
- ◯ now about 48 year old
- ◯ now about 49 year old
- ◯ now about 50 year old
- ◯ now about 51 year old
- ◯ now about 52 year old
- ◯ now about 53 year old
- ◯ now about 54 year old
- ◯ now about 55 year old
- ◯ now about 56 year old
- ◯ now about 57 year old
- ◯ now about 58 year old
- ◯ now about 59 year old
- ◯ now about 60 year old
- ◯ now about 61 year old
- ◯ now about 62 year old
- ◯ now about 63 year old
- ◯ now about 64 year old
- ◯ now about 65 year old
- ◯ now about 66 year old
- ◯ now about 67 year old
- ◯ now about 68 year old

◯ now about 69 year old
◯ now about 70 year old
◯ now about 71 year old
◯ now about 72 year old
◯ now about 73 year old
◯ now about 74 year old
◯ now about 75 year old
◯ now about 76 year old
◯ now about 77 year old
◯ now about 78 year old
◯ now about 79 year old
◯ now about 80 year old
◯ now about 81 year old
◯ now about 82 year old
◯ now about 83 year old
◯ now about 84 year old
◯ now about 85 year old
◯ now about 86 year old
◯ now about 87 year old
◯ now about 88 year old
◯ now about 89 year old
◯ now about 90 years or older

HH memb 15: Age of listed person

- < 1 years old
- now about 1 year old
- now about 2 year old
- now about 3 year old
- now about 4 year old
- now about 5 year old
- now about 6 year old
- now about 7 year old
- now about 8 year old
- now about 9 year old
- now about 10 year old
- now about 11 year old
- now about 12 year old
- now about 13 year old
- now about 14 year old
- now about 15 year old
- now about 16 year old
- now about 17 year old
- now about 18 year old
- now about 19 year old
- now about 20 year old
- now about 21 year old
- now about 22 year old
- now about 23 year old
- now about 24 year old
- now about 25 year old
- now about 26 year old
- now about 27 year old
- now about 28 year old
- now about 29 year old
- now about 30 year old
- now about 31 year old
- now about 32 year old
- now about 33 year old
- now about 34 year old
- now about 35 year old
- now about 36 year old
- now about 37 year old
- now about 38 year old
- now about 39 year old
- now about 40 year old
- now about 41 year old
- now about 42 year old
- now about 43 year old
- now about 44 year old
- now about 45 year old
- now about 46 year old
- now about 47 year old
- now about 48 year old
- now about 49 year old
- now about 50 year old
- now about 51 year old
- now about 52 year old
- now about 53 year old
- now about 54 year old
- now about 55 year old
- now about 56 year old
- now about 57 year old
- now about 58 year old
- now about 59 year old
- now about 60 year old
- now about 61 year old
- now about 62 year old
- now about 63 year old
- now about 64 year old
- now about 65 year old
- now about 66 year old
- now about 67 year old
- now about 68 year old

◯ now about 69 year old
◯ now about 70 year old
◯ now about 71 year old
◯ now about 72 year old
◯ now about 73 year old
◯ now about 74 year old
◯ now about 75 year old
◯ now about 76 year old
◯ now about 77 year old
◯ now about 78 year old
◯ now about 79 year old
◯ now about 80 year old
◯ now about 81 year old
◯ now about 82 year old
◯ now about 83 year old
◯ now about 84 year old
◯ now about 85 year old
◯ now about 86 year old
◯ now about 87 year old
◯ now about 88 year old
◯ now about 89 year old
◯ now about 90 years or older

HH memb 16: Age of listed person

◯ < 1 years old
◯ now about 1 year old
◯ now about 2 year old
◯ now about 3 year old
◯ now about 4 year old
◯ now about 5 year old
◯ now about 6 year old
◯ now about 7 year old
◯ now about 8 year old
◯ now about 9 year old
◯ now about 10 year old
◯ now about 11 year old
◯ now about 12 year old
◯ now about 13 year old
◯ now about 14 year old
◯ now about 15 year old
◯ now about 16 year old
◯ now about 17 year old
◯ now about 18 year old
◯ now about 19 year old
◯ now about 20 year old
◯ now about 21 year old
◯ now about 22 year old
◯ now about 23 year old
◯ now about 24 year old
◯ now about 25 year old
◯ now about 26 year old
◯ now about 27 year old
◯ now about 28 year old
◯ now about 29 year old
◯ now about 30 year old
◯ now about 31 year old
◯ now about 32 year old
◯ now about 33 year old
◯ now about 34 year old
◯ now about 35 year old
◯ now about 36 year old
◯ now about 37 year old
◯ now about 38 year old
◯ now about 39 year old
◯ now about 40 year old
◯ now about 41 year old
◯ now about 42 year old
◯ now about 43 year old
◯ now about 44 year old
◯ now about 45 year old
◯ now about 46 year old
◯ now about 47 year old
◯ now about 48 year old
◯ now about 49 year old
◯ now about 50 year old
◯ now about 51 year old
◯ now about 52 year old
◯ now about 53 year old
◯ now about 54 year old
◯ now about 55 year old
◯ now about 56 year old
◯ now about 57 year old
◯ now about 58 year old
◯ now about 59 year old
◯ now about 60 year old
◯ now about 61 year old
◯ now about 62 year old
◯ now about 63 year old
◯ now about 64 year old
◯ now about 65 year old
◯ now about 66 year old
◯ now about 67 year old
◯ now about 68 year old

◯ now about 69 year old
◯ now about 70 year old
◯ now about 71 year old
◯ now about 72 year old
◯ now about 73 year old
◯ now about 74 year old
◯ now about 75 year old
◯ now about 76 year old
◯ now about 77 year old
◯ now about 78 year old
◯ now about 79 year old
◯ now about 80 year old
◯ now about 81 year old
◯ now about 82 year old
◯ now about 83 year old
◯ now about 84 year old
◯ now about 85 year old
◯ now about 86 year old
◯ now about 87 year old
◯ now about 88 year old
◯ now about 89 year old
◯ now about 90 years or older

HH memb 17: Age of listed person

◯ < 1 years old
◯ now about 1 year old
◯ now about 2 year old
◯ now about 3 year old
◯ now about 4 year old
◯ now about 5 year old
◯ now about 6 year old
◯ now about 7 year old
◯ now about 8 year old
◯ now about 9 year old
◯ now about 10 year old
◯ now about 11 year old
◯ now about 12 year old
◯ now about 13 year old
◯ now about 14 year old
◯ now about 15 year old
◯ now about 16 year old
◯ now about 17 year old
◯ now about 18 year old
◯ now about 19 year old
◯ now about 20 year old
◯ now about 21 year old
◯ now about 22 year old
◯ now about 23 year old
◯ now about 24 year old
◯ now about 25 year old
◯ now about 26 year old
◯ now about 27 year old
◯ now about 28 year old
◯ now about 29 year old
◯ now about 30 year old
◯ now about 31 year old
◯ now about 32 year old
◯ now about 33 year old
◯ now about 34 year old
◯ now about 35 year old
◯ now about 36 year old
◯ now about 37 year old
◯ now about 38 year old
◯ now about 39 year old
◯ now about 40 year old
◯ now about 41 year old
◯ now about 42 year old
◯ now about 43 year old
◯ now about 44 year old
◯ now about 45 year old
◯ now about 46 year old
◯ now about 47 year old
◯ now about 48 year old
◯ now about 49 year old
◯ now about 50 year old
◯ now about 51 year old
◯ now about 52 year old
◯ now about 53 year old
◯ now about 54 year old
◯ now about 55 year old
◯ now about 56 year old
◯ now about 57 year old
◯ now about 58 year old
◯ now about 59 year old
◯ now about 60 year old
◯ now about 61 year old
◯ now about 62 year old
◯ now about 63 year old
◯ now about 64 year old
◯ now about 65 year old
◯ now about 66 year old
◯ now about 67 year old
◯ now about 68 year old

◯ now about 69 year old
◯ now about 70 year old
◯ now about 71 year old
◯ now about 72 year old
◯ now about 73 year old
◯ now about 74 year old
◯ now about 75 year old
◯ now about 76 year old
◯ now about 77 year old
◯ now about 78 year old
◯ now about 79 year old
◯ now about 80 year old
◯ now about 81 year old
◯ now about 82 year old
◯ now about 83 year old
◯ now about 84 year old
◯ now about 85 year old
◯ now about 86 year old
◯ now about 87 year old
◯ now about 88 year old
◯ now about 89 year old
◯ now about 90 years or older

HH memb 18: Age of listed person

- ◯ < 1 years old
- ◯ now about 1 year old
- ◯ now about 2 year old
- ◯ now about 3 year old
- ◯ now about 4 year old
- ◯ now about 5 year old
- ◯ now about 6 year old
- ◯ now about 7 year old
- ◯ now about 8 year old
- ◯ now about 9 year old
- ◯ now about 10 year old
- ◯ now about 11 year old
- ◯ now about 12 year old
- ◯ now about 13 year old
- ◯ now about 14 year old
- ◯ now about 15 year old
- ◯ now about 16 year old
- ◯ now about 17 year old
- ◯ now about 18 year old
- ◯ now about 19 year old
- ◯ now about 20 year old
- ◯ now about 21 year old
- ◯ now about 22 year old
- ◯ now about 23 year old
- ◯ now about 24 year old
- ◯ now about 25 year old
- ◯ now about 26 year old
- ◯ now about 27 year old
- ◯ now about 28 year old
- ◯ now about 29 year old
- ◯ now about 30 year old
- ◯ now about 31 year old
- ◯ now about 32 year old
- ◯ now about 33 year old
- ◯ now about 34 year old
- ◯ now about 35 year old
- ◯ now about 36 year old
- ◯ now about 37 year old
- ◯ now about 38 year old
- ◯ now about 39 year old
- ◯ now about 40 year old
- ◯ now about 41 year old
- ◯ now about 42 year old
- ◯ now about 43 year old
- ◯ now about 44 year old
- ◯ now about 45 year old
- ◯ now about 46 year old
- ◯ now about 47 year old
- ◯ now about 48 year old
- ◯ now about 49 year old
- ◯ now about 50 year old
- ◯ now about 51 year old
- ◯ now about 52 year old
- ◯ now about 53 year old
- ◯ now about 54 year old
- ◯ now about 55 year old
- ◯ now about 56 year old
- ◯ now about 57 year old
- ◯ now about 58 year old
- ◯ now about 59 year old
- ◯ now about 60 year old
- ◯ now about 61 year old
- ◯ now about 62 year old
- ◯ now about 63 year old
- ◯ now about 64 year old
- ◯ now about 65 year old
- ◯ now about 66 year old
- ◯ now about 67 year old
- ◯ now about 68 year old

◯ now about 69 year old
◯ now about 70 year old
◯ now about 71 year old
◯ now about 72 year old
◯ now about 73 year old
◯ now about 74 year old
◯ now about 75 year old
◯ now about 76 year old
◯ now about 77 year old
◯ now about 78 year old
◯ now about 79 year old
◯ now about 80 year old
◯ now about 81 year old
◯ now about 82 year old
◯ now about 83 year old
◯ now about 84 year old
◯ now about 85 year old
◯ now about 86 year old
◯ now about 87 year old
◯ now about 88 year old
◯ now about 89 year old
◯ now about 90 years or older

HH memb 19: Age of listed person

◯ < 1 years old
◯ now about 1 year old
◯ now about 2 year old
◯ now about 3 year old
◯ now about 4 year old
◯ now about 5 year old
◯ now about 6 year old
◯ now about 7 year old
◯ now about 8 year old
◯ now about 9 year old
◯ now about 10 year old
◯ now about 11 year old
◯ now about 12 year old
◯ now about 13 year old
◯ now about 14 year old
◯ now about 15 year old
◯ now about 16 year old
◯ now about 17 year old
◯ now about 18 year old
◯ now about 19 year old
◯ now about 20 year old
◯ now about 21 year old
◯ now about 22 year old
◯ now about 23 year old
◯ now about 24 year old
◯ now about 25 year old
◯ now about 26 year old
◯ now about 27 year old
◯ now about 28 year old
◯ now about 29 year old
◯ now about 30 year old
◯ now about 31 year old
◯ now about 32 year old
◯ now about 33 year old
◯ now about 34 year old
◯ now about 35 year old
◯ now about 36 year old
◯ now about 37 year old
◯ now about 38 year old
◯ now about 39 year old
◯ now about 40 year old
◯ now about 41 year old
◯ now about 42 year old
◯ now about 43 year old
◯ now about 44 year old
◯ now about 45 year old
◯ now about 46 year old
◯ now about 47 year old
◯ now about 48 year old
◯ now about 49 year old
◯ now about 50 year old
◯ now about 51 year old
◯ now about 52 year old
◯ now about 53 year old
◯ now about 54 year old
◯ now about 55 year old
◯ now about 56 year old
◯ now about 57 year old
◯ now about 58 year old
◯ now about 59 year old
◯ now about 60 year old
◯ now about 61 year old
◯ now about 62 year old
◯ now about 63 year old
◯ now about 64 year old
◯ now about 65 year old
◯ now about 66 year old
◯ now about 67 year old
◯ now about 68 year old

◯ now about 69 year old
◯ now about 70 year old
◯ now about 71 year old
◯ now about 72 year old
◯ now about 73 year old
◯ now about 74 year old
◯ now about 75 year old
◯ now about 76 year old
◯ now about 77 year old
◯ now about 78 year old
◯ now about 79 year old
◯ now about 80 year old
◯ now about 81 year old
◯ now about 82 year old
◯ now about 83 year old
◯ now about 84 year old
◯ now about 85 year old
◯ now about 86 year old
◯ now about 87 year old
◯ now about 88 year old
◯ now about 89 year old
◯ now about 90 years or older

HH memb 20: Age of listed person

- ◯ < 1 years old
- ◯ now about 1 year old
- ◯ now about 2 year old
- ◯ now about 3 year old
- ◯ now about 4 year old
- ◯ now about 5 year old
- ◯ now about 6 year old
- ◯ now about 7 year old
- ◯ now about 8 year old
- ◯ now about 9 year old
- ◯ now about 10 year old
- ◯ now about 11 year old
- ◯ now about 12 year old
- ◯ now about 13 year old
- ◯ now about 14 year old
- ◯ now about 15 year old
- ◯ now about 16 year old
- ◯ now about 17 year old
- ◯ now about 18 year old
- ◯ now about 19 year old
- ◯ now about 20 year old
- ◯ now about 21 year old
- ◯ now about 22 year old
- ◯ now about 23 year old
- ◯ now about 24 year old
- ◯ now about 25 year old
- ◯ now about 26 year old
- ◯ now about 27 year old
- ◯ now about 28 year old
- ◯ now about 29 year old
- ◯ now about 30 year old
- ◯ now about 31 year old
- ◯ now about 32 year old
- ◯ now about 33 year old
- ◯ now about 34 year old
- ◯ now about 35 year old
- ◯ now about 36 year old
- ◯ now about 37 year old
- ◯ now about 38 year old
- ◯ now about 39 year old
- ◯ now about 40 year old
- ◯ now about 41 year old
- ◯ now about 42 year old
- ◯ now about 43 year old
- ◯ now about 44 year old
- ◯ now about 45 year old
- ◯ now about 46 year old
- ◯ now about 47 year old
- ◯ now about 48 year old
- ◯ now about 49 year old
- ◯ now about 50 year old
- ◯ now about 51 year old
- ◯ now about 52 year old
- ◯ now about 53 year old
- ◯ now about 54 year old
- ◯ now about 55 year old
- ◯ now about 56 year old
- ◯ now about 57 year old
- ◯ now about 58 year old
- ◯ now about 59 year old
- ◯ now about 60 year old
- ◯ now about 61 year old
- ◯ now about 62 year old
- ◯ now about 63 year old
- ◯ now about 64 year old
- ◯ now about 65 year old
- ◯ now about 66 year old
- ◯ now about 67 year old
- ◯ now about 68 year old

◯ now about 69 year old
◯ now about 70 year old
◯ now about 71 year old
◯ now about 72 year old
◯ now about 73 year old
◯ now about 74 year old
◯ now about 75 year old
◯ now about 76 year old
◯ now about 77 year old
◯ now about 78 year old
◯ now about 79 year old
◯ now about 80 year old
◯ now about 81 year old
◯ now about 82 year old
◯ now about 83 year old
◯ now about 84 year old
◯ now about 85 year old
◯ now about 86 year old
◯ now about 87 year old
◯ now about 88 year old
◯ now about 89 year old
◯ now about 90 years or older

HH memb 21: Age of listed person

- ◯ < 1 years old
- ◯ now about 1 year old
- ◯ now about 2 year old
- ◯ now about 3 year old
- ◯ now about 4 year old
- ◯ now about 5 year old
- ◯ now about 6 year old
- ◯ now about 7 year old
- ◯ now about 8 year old
- ◯ now about 9 year old
- ◯ now about 10 year old
- ◯ now about 11 year old
- ◯ now about 12 year old
- ◯ now about 13 year old
- ◯ now about 14 year old
- ◯ now about 15 year old
- ◯ now about 16 year old
- ◯ now about 17 year old
- ◯ now about 18 year old
- ◯ now about 19 year old
- ◯ now about 20 year old
- ◯ now about 21 year old
- ◯ now about 22 year old
- ◯ now about 23 year old
- ◯ now about 24 year old
- ◯ now about 25 year old
- ◯ now about 26 year old
- ◯ now about 27 year old
- ◯ now about 28 year old
- ◯ now about 29 year old
- ◯ now about 30 year old
- ◯ now about 31 year old
- ◯ now about 32 year old
- ◯ now about 33 year old
- ◯ now about 34 year old
- ◯ now about 35 year old
- ◯ now about 36 year old
- ◯ now about 37 year old
- ◯ now about 38 year old
- ◯ now about 39 year old
- ◯ now about 40 year old
- ◯ now about 41 year old
- ◯ now about 42 year old
- ◯ now about 43 year old
- ◯ now about 44 year old
- ◯ now about 45 year old
- ◯ now about 46 year old
- ◯ now about 47 year old
- ◯ now about 48 year old
- ◯ now about 49 year old
- ◯ now about 50 year old
- ◯ now about 51 year old
- ◯ now about 52 year old
- ◯ now about 53 year old
- ◯ now about 54 year old
- ◯ now about 55 year old
- ◯ now about 56 year old
- ◯ now about 57 year old
- ◯ now about 58 year old
- ◯ now about 59 year old
- ◯ now about 60 year old
- ◯ now about 61 year old
- ◯ now about 62 year old
- ◯ now about 63 year old
- ◯ now about 64 year old
- ◯ now about 65 year old
- ◯ now about 66 year old
- ◯ now about 67 year old
- ◯ now about 68 year old

◯ now about 69 year old
◯ now about 70 year old
◯ now about 71 year old
◯ now about 72 year old
◯ now about 73 year old
◯ now about 74 year old
◯ now about 75 year old
◯ now about 76 year old
◯ now about 77 year old
◯ now about 78 year old
◯ now about 79 year old
◯ now about 80 year old
◯ now about 81 year old
◯ now about 82 year old
◯ now about 83 year old
◯ now about 84 year old
◯ now about 85 year old
◯ now about 86 year old
◯ now about 87 year old
◯ now about 88 year old
◯ now about 89 year old
◯ now about 90 years or older

HH memb 22: Age of listed person

◯ < 1 years old
◯ now about 1 year old
◯ now about 2 year old
◯ now about 3 year old
◯ now about 4 year old
◯ now about 5 year old
◯ now about 6 year old
◯ now about 7 year old
◯ now about 8 year old
◯ now about 9 year old
◯ now about 10 year old
◯ now about 11 year old
◯ now about 12 year old
◯ now about 13 year old
◯ now about 14 year old
◯ now about 15 year old
◯ now about 16 year old
◯ now about 17 year old
◯ now about 18 year old
◯ now about 19 year old
◯ now about 20 year old
◯ now about 21 year old
◯ now about 22 year old
◯ now about 23 year old
◯ now about 24 year old
◯ now about 25 year old
◯ now about 26 year old
◯ now about 27 year old
◯ now about 28 year old
◯ now about 29 year old
◯ now about 30 year old
◯ now about 31 year old
◯ now about 32 year old
◯ now about 33 year old
◯ now about 34 year old
◯ now about 35 year old
◯ now about 36 year old
◯ now about 37 year old
◯ now about 38 year old
◯ now about 39 year old
◯ now about 40 year old
◯ now about 41 year old
◯ now about 42 year old
◯ now about 43 year old
◯ now about 44 year old
◯ now about 45 year old
◯ now about 46 year old
◯ now about 47 year old
◯ now about 48 year old
◯ now about 49 year old
◯ now about 50 year old
◯ now about 51 year old
◯ now about 52 year old
◯ now about 53 year old
◯ now about 54 year old
◯ now about 55 year old
◯ now about 56 year old
◯ now about 57 year old
◯ now about 58 year old
◯ now about 59 year old
◯ now about 60 year old
◯ now about 61 year old
◯ now about 62 year old
◯ now about 63 year old
◯ now about 64 year old
◯ now about 65 year old
◯ now about 66 year old
◯ now about 67 year old
◯ now about 68 year old

	◯ now about 69 year old<br>◯ now about 70 year old<br>◯ now about 71 year old<br>◯ now about 72 year old<br>◯ now about 73 year old<br>◯ now about 74 year old<br>◯ now about 75 year old<br>◯ now about 76 year old<br>◯ now about 77 year old<br>◯ now about 78 year old<br>◯ now about 79 year old<br>◯ now about 80 year old<br>◯ now about 81 year old<br>◯ now about 82 year old<br>◯ now about 83 year old<br>◯ now about 84 year old<br>◯ now about 85 year old<br>◯ now about 86 year old<br>◯ now about 87 year old<br>◯ now about 88 year old<br>◯ now about 89 year old<br>◯ now about 90 years or older
HH memb 1: Vital Status	◯ Alive (at last survey)<br>◯ Died during the last year<br>◯ Died during the last 2 years<br>◯ Died during the last 5 years<br>◯ Died more than 5 years ago<br>◯ Died, but timing not known<br>◯ Unknown vital status
HH memb 2: Vital Status	◯ Alive (at last survey)<br>◯ Died during the last year<br>◯ Died during the last 2 years<br>◯ Died during the last 5 years<br>◯ Died more than 5 years ago<br>◯ Died, but timing not known<br>◯ Unknown vital status
HH memb 3: Vital Status	◯ Alive (at last survey)<br>◯ Died during the last year<br>◯ Died during the last 2 years<br>◯ Died during the last 5 years<br>◯ Died more than 5 years ago<br>◯ Died, but timing not known<br>◯ Unknown vital status
HH memb 4: Vital Status	◯ Alive (at last survey)<br>◯ Died during the last year<br>◯ Died during the last 2 years<br>◯ Died during the last 5 years<br>◯ Died more than 5 years ago<br>◯ Died, but timing not known<br>◯ Unknown vital status
HH memb 5: Vital Status	◯ Alive (at last survey)<br>◯ Died during the last year<br>◯ Died during the last 2 years<br>◯ Died during the last 5 years<br>◯ Died more than 5 years ago<br>◯ Died, but timing not known<br>◯ Unknown vital status

HH memb 6: Vital Status	◯ Alive (at last survey)<br>◯ Died during the last year<br>◯ Died during the last 2 years<br>◯ Died during the last 5 years<br>◯ Died more than 5 years ago<br>◯ Died, but timing not known<br>◯ Unknown vital status
HH memb 7: Vital Status	◯ Alive (at last survey)<br>◯ Died during the last year<br>◯ Died during the last 2 years<br>◯ Died during the last 5 years<br>◯ Died more than 5 years ago<br>◯ Died, but timing not known<br>◯ Unknown vital status
HH memb 8: Vital Status	◯ Alive (at last survey)<br>◯ Died during the last year<br>◯ Died during the last 2 years<br>◯ Died during the last 5 years<br>◯ Died more than 5 years ago<br>◯ Died, but timing not known<br>◯ Unknown vital status
HH memb 9: Vital Status	◯ Alive (at last survey)<br>◯ Died during the last year<br>◯ Died during the last 2 years<br>◯ Died during the last 5 years<br>◯ Died more than 5 years ago<br>◯ Died, but timing not known<br>◯ Unknown vital status
HH memb 10: Vital Status	◯ Alive (at last survey)<br>◯ Died during the last year<br>◯ Died during the last 2 years<br>◯ Died during the last 5 years<br>◯ Died more than 5 years ago<br>◯ Died, but timing not known<br>◯ Unknown vital status
HH memb 11: Vital Status	◯ Alive (at last survey)<br>◯ Died during the last year<br>◯ Died during the last 2 years<br>◯ Died during the last 5 years<br>◯ Died more than 5 years ago<br>◯ Died, but timing not known<br>◯ Unknown vital status
HH memb 12: Vital Status	◯ Alive (at last survey)<br>◯ Died during the last year<br>◯ Died during the last 2 years<br>◯ Died during the last 5 years<br>◯ Died more than 5 years ago<br>◯ Died, but timing not known<br>◯ Unknown vital status

HH memb 13: Vital Status	◯ Alive (at last survey)<br>◯ Died during the last year<br>◯ Died during the last 2 years<br>◯ Died during the last 5 years<br>◯ Died more than 5 years ago<br>◯ Died, but timing not known<br>◯ Unknown vital status
HH memb 14: Vital Status	◯ Alive (at last survey)<br>◯ Died during the last year<br>◯ Died during the last 2 years<br>◯ Died during the last 5 years<br>◯ Died more than 5 years ago<br>◯ Died, but timing not known<br>◯ Unknown vital status
HH memb 15: Vital Status	◯ Alive (at last survey)<br>◯ Died during the last year<br>◯ Died during the last 2 years<br>◯ Died during the last 5 years<br>◯ Died more than 5 years ago<br>◯ Died, but timing not known<br>◯ Unknown vital status
HH memb 16: Vital Status	◯ Alive (at last survey)<br>◯ Died during the last year<br>◯ Died during the last 2 years<br>◯ Died during the last 5 years<br>◯ Died more than 5 years ago<br>◯ Died, but timing not known<br>◯ Unknown vital status
HH memb 17: Vital Status	◯ Alive (at last survey)<br>◯ Died during the last year<br>◯ Died during the last 2 years<br>◯ Died during the last 5 years<br>◯ Died more than 5 years ago<br>◯ Died, but timing not known<br>◯ Unknown vital status
HH memb 18: Vital Status	◯ Alive (at last survey)<br>◯ Died during the last year<br>◯ Died during the last 2 years<br>◯ Died during the last 5 years<br>◯ Died more than 5 years ago<br>◯ Died, but timing not known<br>◯ Unknown vital status
HH memb 19: Vital Status	◯ Alive (at last survey)<br>◯ Died during the last year<br>◯ Died during the last 2 years<br>◯ Died during the last 5 years<br>◯ Died more than 5 years ago<br>◯ Died, but timing not known<br>◯ Unknown vital status

HH memb 20: Vital Status

◯ Alive (at last survey)
◯ Died during the last year
◯ Died during the last 2 years
◯ Died during the last 5 years
◯ Died more than 5 years ago
◯ Died, but timing not known
◯ Unknown vital status

HH memb 21: Vital Status

◯ Alive (at last survey)
◯ Died during the last year
◯ Died during the last 2 years
◯ Died during the last 5 years
◯ Died more than 5 years ago
◯ Died, but timing not known
◯ Unknown vital status

HH memb 22: Vital Status

◯ Alive (at last survey)
◯ Died during the last year
◯ Died during the last 2 years
◯ Died during the last 5 years
◯ Died more than 5 years ago
◯ Died, but timing not known
◯ Unknown vital status

Time

____

Respondent

[respname] (RespID: [respid])
([gender], about [age] years old, [maritalstatus] at last survey; village: [villagename], region: [region], previous survey: [prior_surv_year], conducted in [prior_surv_language])

Contact Info / Phone Numbers

INTERVIEWER: Below are up to 3 existing phone numbers, and options to enter two additional numbers

[cv1_phone1] (belongs to [cv1_phone1_name], [cv1_phone1_belong])

◯ Check to delete phone from call log

[cv1_phone2] (belongs to [cv1_phone2_name], [cv1_phone2_belong])

◯ Check to delete phone from call log

[cv1_phone3] (belongs to [cv1_phone3_name], [cv1_phone3_belong])

◯ Check to delete phone from call log

Additional (new) Phone:

____

Whose Phone is this:

◯ Own (Respondent)
◯ Child
◯ Spouse
◯ Household member
◯ Relative outside of household
◯ Friend/neighbor
◯ Secondary Contact Person
◯ Other/Unknown relationship

Full Name of owner of phone: ______________________

Additional (new) Phone: ______________________

Whose Phone is this:
- ◯ Own (Respondent)
- ◯ Child
- ◯ Spouse
- ◯ Household member
- ◯ Relative outside of household
- ◯ Friend/neighbor
- ◯ Secondary Contact Person
- ◯ Other/Unknown relationship

Full Name of owner of phone: ______________________

Additional (new) Phone: ______________________

Whose Phone is this:
- ◯ Own (Respondent)
- ◯ Child
- ◯ Spouse
- ◯ Household member
- ◯ Relative outside of household
- ◯ Friend/neighbor
- ◯ Secondary Contact Person
- ◯ Other/Unknown relationship

Full Name of owner of phone: ______________________

INTERVIEWER: Add here comments that help you contact the respondents (do NOT enter phone numbers here!) ______________________

Interviewer Info

INTERVIEWER: Select your (= Interviewer) name:
- ◯ List of Interviewers

Call Log

INTERVIEWER: Log below below all call attempts to reach respondent

CALL_1: Call Attempt 1 ______________________
(Day Month Year Hours:Min, entered as 15-04-2018 17:45)

Phones called during Call Attempt 1:

a): [cv1_phone1] (belongs to [cv1_phone1_name], [cv1_phone1_belong])	◯ Success (proceed to respondent identification)<br>◯ Respondent not available (rescheduled)<br>◯ Reached informant / proxy interview<br>◯ Reached informant (no proxy interview)<br>◯ Informant not available (rescheduled)<br>◯ No answer / No connection<br>◯ Phone disconnected / Phone not on network<br>◯ Wrong person / respondent not known at number<br>◯ Number not called this time
b): [cv1_phone2] (belongs to [cv1_phone2_name], [cv1_phone2_belong])	◯ Success (proceed to respondent identification)<br>◯ Respondent not available (rescheduled)<br>◯ Reached informant / proxy interview<br>◯ Reached informant (no proxy interview)<br>◯ Informant not available (rescheduled)<br>◯ No answer / No connection<br>◯ Phone disconnected / Phone not on network<br>◯ Wrong person / respondent not known at number<br>◯ Number not called this time
c): [cv1_phone3] (belongs to [cv1_phone3_name], [cv1_phone3_belong])	◯ Success (proceed to respondent identification)<br>◯ Respondent not available (rescheduled)<br>◯ Reached informant / proxy interview<br>◯ Reached informant (no proxy interview)<br>◯ Informant not available (rescheduled)<br>◯ No answer / No connection<br>◯ Phone disconnected / Phone not on network<br>◯ Wrong person / respondent not known at number<br>◯ Number not called this time
d): [cv1_phone4] (belongs to [cv1_phone4_name], [cv1_phone4_belong])	◯ Success (proceed to respondent identification)<br>◯ Respondent not available (rescheduled)<br>◯ Reached informant / proxy interview<br>◯ Reached informant (no proxy interview)<br>◯ Informant not available (rescheduled)<br>◯ No answer / No connection<br>◯ Phone disconnected / Phone not on network<br>◯ Wrong person / respondent not known at number<br>◯ Number not called this time
e): [cv1_phone5] (belongs to [cv1_phone5_name], [cv1_phone5_belong])	◯ Success (proceed to respondent identification)<br>◯ Respondent not available (rescheduled)<br>◯ Reached informant / proxy interview<br>◯ Reached informant (no proxy interview)<br>◯ Informant not available (rescheduled)<br>◯ No answer / No connection<br>◯ Phone disconnected / Phone not on network<br>◯ Wrong person / respondent not known at number<br>◯ Number not called this time
f): [cv1_phone6] (belongs to [cv1_phone6_name], [cv1_phone6_belong])	◯ Success (proceed to respondent identification)<br>◯ Respondent not available (rescheduled)<br>◯ Reached informant / proxy interview<br>◯ Reached informant (no proxy interview)<br>◯ Informant not available (rescheduled)<br>◯ No answer / No connection<br>◯ Phone disconnected / Phone not on network<br>◯ Wrong person / respondent not known at number<br>◯ Number not called this time

CALL_2: Call Attempt 2

____
(Day Month Year Hours:Min, entered as 15-04-2018 17:45)

Phones called during Call Attempt 2:

a): [cv1_phone1] (belongs to [cv1_phone1_name], [cv1_phone1_belong])

◯ Success (proceed to respondent identification)
◯ Respondent not available (rescheduled)
◯ Reached informant / proxy interview
◯ Reached informant (no proxy interview)
◯ Informant not available (rescheduled)
◯ No answer / No connection
◯ Phone disconnected / Phone not on network
◯ Wrong person / respondent not known at number
◯ Number not called this time

b): [cv1_phone2] (belongs to [cv1_phone2_name], [cv1_phone2_belong])

◯ Success (proceed to respondent identification)
◯ Respondent not available (rescheduled)
◯ Reached informant / proxy interview
◯ Reached informant (no proxy interview)
◯ Informant not available (rescheduled)
◯ No answer / No connection
◯ Phone disconnected / Phone not on network
◯ Wrong person / respondent not known at number
◯ Number not called this time

c): [cv1_phone3] (belongs to [cv1_phone3_name], [cv1_phone3_belong])

◯ Success (proceed to respondent identification)
◯ Respondent not available (rescheduled)
◯ Reached informant / proxy interview
◯ Reached informant (no proxy interview)
◯ Informant not available (rescheduled)
◯ No answer / No connection
◯ Phone disconnected / Phone not on network
◯ Wrong person / respondent not known at number
◯ Number not called this time

d): [cv1_phone4] (belongs to [cv1_phone4_name], [cv1_phone4_belong])

◯ Success (proceed to respondent identification)
◯ Respondent not available (rescheduled)
◯ Reached informant / proxy interview
◯ Reached informant (no proxy interview)
◯ Informant not available (rescheduled)
◯ No answer / No connection
◯ Phone disconnected / Phone not on network
◯ Wrong person / respondent not known at number
◯ Number not called this time

e): [cv1_phone5] (belongs to [cv1_phone5_name], [cv1_phone5_belong])

◯ Success (proceed to respondent identification)
◯ Respondent not available (rescheduled)
◯ Reached informant / proxy interview
◯ Reached informant (no proxy interview)
◯ Informant not available (rescheduled)
◯ No answer / No connection
◯ Phone disconnected / Phone not on network
◯ Wrong person / respondent not known at number
◯ Number not called this time

f): [cv1_phone6] (belongs to [cv1_phone6_name], [cv1_phone6_belong])

◯ Success (proceed to respondent identification)
◯ Respondent not available (rescheduled)
◯ Reached informant / proxy interview
◯ Reached informant (no proxy interview)
◯ Informant not available (rescheduled)
◯ No answer / No connection
◯ Phone disconnected / Phone not on network
◯ Wrong person / respondent not known at number
◯ Number not called this time

CALL_3: Call Attempt 3

____
(Day Month Year Hours:Min, entered as 15-04-2018 17:45)

Phones called during Call Attempt 3:

a): [cv1_phone1] (belongs to [cv1_phone1_name], [cv1_phone1_belong])

◯ Success (proceed to respondent identification)
◯ Respondent not available (rescheduled)
◯ Reached informant / proxy interview
◯ Reached informant (no proxy interview)
◯ Informant not available (rescheduled)
◯ No answer / No connection
◯ Phone disconnected / Phone not on network
◯ Wrong person / respondent not known at number
◯ Number not called this time

b): [cv1_phone2] (belongs to [cv1_phone2_name], [cv1_phone2_belong])

◯ Success (proceed to respondent identification)
◯ Respondent not available (rescheduled)
◯ Reached informant / proxy interview
◯ Reached informant (no proxy interview)
◯ Informant not available (rescheduled)
◯ No answer / No connection
◯ Phone disconnected / Phone not on network
◯ Wrong person / respondent not known at number
◯ Number not called this time

c): [cv1_phone3] (belongs to [cv1_phone3_name], [cv1_phone3_belong])

◯ Success (proceed to respondent identification)
◯ Respondent not available (rescheduled)
◯ Reached informant / proxy interview
◯ Reached informant (no proxy interview)
◯ Informant not available (rescheduled)
◯ No answer / No connection
◯ Phone disconnected / Phone not on network
◯ Wrong person / respondent not known at number
◯ Number not called this time

d): [cv1_phone4] (belongs to [cv1_phone4_name], [cv1_phone4_belong])

◯ Success (proceed to respondent identification)
◯ Respondent not available (rescheduled)
◯ Reached informant / proxy interview
◯ Reached informant (no proxy interview)
◯ Informant not available (rescheduled)
◯ No answer / No connection
◯ Phone disconnected / Phone not on network
◯ Wrong person / respondent not known at number
◯ Number not called this time

e): [cv1_phone5] (belongs to [cv1_phone5_name], [cv1_phone5_belong])	◯ Success (proceed to respondent identification)<br>◯ Respondent not available (rescheduled)<br>◯ Reached informant / proxy interview<br>◯ Reached informant (no proxy interview)<br>◯ Informant not available (rescheduled)<br>◯ No answer / No connection<br>◯ Phone disconnected / Phone not on network<br>◯ Wrong person / respondent not known at number<br>◯ Number not called this time
f): [cv1_phone6] (belongs to [cv1_phone6_name], [cv1_phone6_belong])	◯ Success (proceed to respondent identification)<br>◯ Respondent not available (rescheduled)<br>◯ Reached informant / proxy interview<br>◯ Reached informant (no proxy interview)<br>◯ Informant not available (rescheduled)<br>◯ No answer / No connection<br>◯ Phone disconnected / Phone not on network<br>◯ Wrong person / respondent not known at number<br>◯ Number not called this time
CALL_4: Call Attempt 4	____________________________<br>(Day Month Year Hours:Min, entered as 15-04-2018 17:45)
Phones called during Call Attempt 4:	
a): [cv1_phone1] (belongs to [cv1_phone1_name], [cv1_phone1_belong])	◯ Success (proceed to respondent identification)<br>◯ Respondent not available (rescheduled)<br>◯ Reached informant / proxy interview<br>◯ Reached informant (no proxy interview)<br>◯ Informant not available (rescheduled)<br>◯ No answer / No connection<br>◯ Phone disconnected / Phone not on network<br>◯ Wrong person / respondent not known at number<br>◯ Number not called this time
b): [cv1_phone2] (belongs to [cv1_phone2_name], [cv1_phone2_belong])	◯ Success (proceed to respondent identification)<br>◯ Respondent not available (rescheduled)<br>◯ Reached informant / proxy interview<br>◯ Reached informant (no proxy interview)<br>◯ Informant not available (rescheduled)<br>◯ No answer / No connection<br>◯ Phone disconnected / Phone not on network<br>◯ Wrong person / respondent not known at number<br>◯ Number not called this time
c): [cv1_phone3] (belongs to [cv1_phone3_name], [cv1_phone3_belong])	◯ Success (proceed to respondent identification)<br>◯ Respondent not available (rescheduled)<br>◯ Reached informant / proxy interview<br>◯ Reached informant (no proxy interview)<br>◯ Informant not available (rescheduled)<br>◯ No answer / No connection<br>◯ Phone disconnected / Phone not on network<br>◯ Wrong person / respondent not known at number<br>◯ Number not called this time

d): [cv1_phone4] (belongs to [cv1_phone4_name], [cv1_phone4_belong])

◯ Success (proceed to respondent identification)
◯ Respondent not available (rescheduled)
◯ Reached informant / proxy interview
◯ Reached informant (no proxy interview)
◯ Informant not available (rescheduled)
◯ No answer / No connection
◯ Phone disconnected / Phone not on network
◯ Wrong person / respondent not known at number
◯ Number not called this time

e): [cv1_phone5] (belongs to [cv1_phone5_name], [cv1_phone5_belong])

◯ Success (proceed to respondent identification)
◯ Respondent not available (rescheduled)
◯ Reached informant / proxy interview
◯ Reached informant (no proxy interview)
◯ Informant not available (rescheduled)
◯ No answer / No connection
◯ Phone disconnected / Phone not on network
◯ Wrong person / respondent not known at number
◯ Number not called this time

f): [cv1_phone6] (belongs to [cv1_phone6_name], [cv1_phone6_belong])

◯ Success (proceed to respondent identification)
◯ Respondent not available (rescheduled)
◯ Reached informant / proxy interview
◯ Reached informant (no proxy interview)
◯ Informant not available (rescheduled)
◯ No answer / No connection
◯ Phone disconnected / Phone not on network
◯ Wrong person / respondent not known at number
◯ Number not called this time

CALL_5: Call Attempt 5

____
(Day Month Year Hours:Min, entered as 15-04-2018 17:45)

Phones called during Call Attempt 5:

a): [cv1_phone1] (belongs to [cv1_phone1_name], [cv1_phone1_belong])

◯ Success (proceed to respondent identification)
◯ Respondent not available (rescheduled)
◯ Reached informant / proxy interview
◯ Reached informant (no proxy interview)
◯ Informant not available (rescheduled)
◯ No answer / No connection
◯ Phone disconnected / Phone not on network
◯ Wrong person / respondent not known at number
◯ Number not called this time

b): [cv1_phone2] (belongs to [cv1_phone2_name], [cv1_phone2_belong])

◯ Success (proceed to respondent identification)
◯ Respondent not available (rescheduled)
◯ Reached informant / proxy interview
◯ Reached informant (no proxy interview)
◯ Informant not available (rescheduled)
◯ No answer / No connection
◯ Phone disconnected / Phone not on network
◯ Wrong person / respondent not known at number
◯ Number not called this time

c): [cv1_phone3] (belongs to [cv1_phone3_name], [cv1_phone3_belong])	◯ Success (proceed to respondent identification)<br>◯ Respondent not available (rescheduled)<br>◯ Reached informant / proxy interview<br>◯ Reached informant (no proxy interview)<br>◯ Informant not available (rescheduled)<br>◯ No answer / No connection<br>◯ Phone disconnected / Phone not on network<br>◯ Wrong person / respondent not known at number<br>◯ Number not called this time
d): [cv1_phone4] (belongs to [cv1_phone4_name], [cv1_phone4_belong])	◯ Success (proceed to respondent identification)<br>◯ Respondent not available (rescheduled)<br>◯ Reached informant / proxy interview<br>◯ Reached informant (no proxy interview)<br>◯ Informant not available (rescheduled)<br>◯ No answer / No connection<br>◯ Phone disconnected / Phone not on network<br>◯ Wrong person / respondent not known at number<br>◯ Number not called this time
e): [cv1_phone5] (belongs to [cv1_phone5_name], [cv1_phone5_belong])	◯ Success (proceed to respondent identification)<br>◯ Respondent not available (rescheduled)<br>◯ Reached informant / proxy interview<br>◯ Reached informant (no proxy interview)<br>◯ Informant not available (rescheduled)<br>◯ No answer / No connection<br>◯ Phone disconnected / Phone not on network<br>◯ Wrong person / respondent not known at number<br>◯ Number not called this time
f): [cv1_phone6] (belongs to [cv1_phone6_name], [cv1_phone6_belong])	◯ Success (proceed to respondent identification)<br>◯ Respondent not available (rescheduled)<br>◯ Reached informant / proxy interview<br>◯ Reached informant (no proxy interview)<br>◯ Informant not available (rescheduled)<br>◯ No answer / No connection<br>◯ Phone disconnected / Phone not on network<br>◯ Wrong person / respondent not known at number<br>◯ Number not called this time
CALL_6: Call Attempt 6	________________________________<br>(Day Month Year Hours:Min, entered as 15-04-2018 17:45)
Phones called during Call Attempt 6:	
a): [cv1_phone1] (belongs to [cv1_phone1_name], [cv1_phone1_belong])	◯ Success (proceed to respondent identification)<br>◯ Respondent not available (rescheduled)<br>◯ Reached informant / proxy interview<br>◯ Reached informant (no proxy interview)<br>◯ Informant not available (rescheduled)<br>◯ No answer / No connection<br>◯ Phone disconnected / Phone not on network<br>◯ Wrong person / respondent not known at number<br>◯ Number not called this time

b): [cv1_phone2] (belongs to [cv1_phone2_name], [cv1_phone2_belong])	◯ Success (proceed to respondent identification)<br>◯ Respondent not available (rescheduled)<br>◯ Reached informant / proxy interview<br>◯ Reached informant (no proxy interview)<br>◯ Informant not available (rescheduled)<br>◯ No answer / No connection<br>◯ Phone disconnected / Phone not on network<br>◯ Wrong person / respondent not known at number<br>◯ Number not called this time
c): [cv1_phone3] (belongs to [cv1_phone3_name], [cv1_phone3_belong])	◯ Success (proceed to respondent identification)<br>◯ Respondent not available (rescheduled)<br>◯ Reached informant / proxy interview<br>◯ Reached informant (no proxy interview)<br>◯ Informant not available (rescheduled)<br>◯ No answer / No connection<br>◯ Phone disconnected / Phone not on network<br>◯ Wrong person / respondent not known at number<br>◯ Number not called this time
d): [cv1_phone4] (belongs to [cv1_phone4_name], [cv1_phone4_belong])	◯ Success (proceed to respondent identification)<br>◯ Respondent not available (rescheduled)<br>◯ Reached informant / proxy interview<br>◯ Reached informant (no proxy interview)<br>◯ Informant not available (rescheduled)<br>◯ No answer / No connection<br>◯ Phone disconnected / Phone not on network<br>◯ Wrong person / respondent not known at number<br>◯ Number not called this time
e): [cv1_phone5] (belongs to [cv1_phone5_name], [cv1_phone5_belong])	◯ Success (proceed to respondent identification)<br>◯ Respondent not available (rescheduled)<br>◯ Reached informant / proxy interview<br>◯ Reached informant (no proxy interview)<br>◯ Informant not available (rescheduled)<br>◯ No answer / No connection<br>◯ Phone disconnected / Phone not on network<br>◯ Wrong person / respondent not known at number<br>◯ Number not called this time
f): [cv1_phone6] (belongs to [cv1_phone6_name], [cv1_phone6_belong])	◯ Success (proceed to respondent identification)<br>◯ Respondent not available (rescheduled)<br>◯ Reached informant / proxy interview<br>◯ Reached informant (no proxy interview)<br>◯ Informant not available (rescheduled)<br>◯ No answer / No connection<br>◯ Phone disconnected / Phone not on network<br>◯ Wrong person / respondent not known at number<br>◯ Number not called this time

Cv1 1b Resp Identification

Time ______________________________

Respondent Identification

INTERVIEWER: Use the below information on the respondent and the respondent's household to verify the identity of the MLSFH respondent

Respondent: [respname] (RespID: [respid])
([gender], about [age] years old, [maritalstatus] at last survey; village: [villagename], region: [region], previous survey: [prior_surv_year], conducted in: [prior_surv_language])

Father's name: [fathers_name]

Birth village and district: [birth_village] ([birth_district])

Compound head: [compoundhead]

Village head: [headname]

Marital status (at last survey): [maritalstatus]

Spouse names (blank if no spouses in data):

[spousename1]

[spousename2]

[spousename3]

[spousename4]

[spousename5]

Household roster information (based on last MLSFH survey, if available)

[m7_rname_1] ([m7_relresp_1], [m7_alive_1], [m7_rage_1])?

[m7_rname_2] ([m7_relresp_2], [m7_alive_2], [m7_rage_2])?

[m7_rname_3] ([m7_relresp_3], [m7_alive_3], [m7_rage_3])?

[m7_rname_4] ([m7_relresp_4], [m7_alive_4], [m7_rage_4])?

[m7_rname_5] ([m7_relresp_5], [m7_alive_5], [m7_rage_5])?

[m7_rname_6] ([m7_relresp_6], [m7_alive_6], [m7_rage_6])?

[m7_rname_7] ([m7_relresp_7], [m7_alive_7], [m7_rage_7])?

[m7_rname_8] ([m7_relresp_8], [m7_alive_8], [m7_rage_8])?

[m7_rname_9] ([m7_relresp_9], [m7_alive_9], [m7_rage_9])?

[m7_rname_10] ([m7_relresp_10], [m7_alive_10], [m7_rage_10])?

[m7_rname_11] ([m7_relresp_11], [m7_alive_11], [m7_rage_11])?

[m7_rname_12] ([m7_relresp_12], [m7_alive_12], [m7_rage_12])?

[m7_rname_13] ([m7_relresp_13], [m7_alive_13], [m7_rage_13])?

[m7_rname_14] ([m7_relresp_14], [m7_alive_14], [m7_rage_14])?

[m7_rname_15] ([m7_relresp_15], [m7_alive_15], [m7_rage_15])?

Select Language for Interview

Select language for interview?

- ◯ Chichewa
- ◯ Tumbuka
- ◯ Yao

Confirm: Interview is conducted in [cv1_language], correct?

- ◯ Yes --> Proceed
- ◯ No --> Select correct language above

Confirm Respondent

INTERVIEWER: Please Confirm: Are you talking to MLSFH respondent [respname] (RespID: [respid])

- ◯ Yes, Respondent found and verified, ready to proceed to Informed Consent
- ◯ Yes, but refusal (not willing to participate in Informed Consent)
- ◯ No, Resp is hospitalized and not available
- ◯ No, Resp has died
- ◯ No, Resp not found or is not known at this number
- ◯ No, Resp temporarily absent
- ◯ No, Resp moved (not expected to return)
- ◯ No, Resp is sick and not available
- ◯ Uncertain if talking to correct respondent
- ◯ Refer respondent to other interviewer (specify reason)
- ◯ Other, specify

Specify: ______________________________

INTERVIEWER: Proceed to Informed Consent

INTERVIEWER: STOP -- Do NOT proceed to Informed Consent

INTERVIEWER: Report respondent to Management Team for further checking and follow-up

Ask questions about deceased respondents ______________________________

Ask questions about sick, hospitalized or absent respondents ______________________

Follow-up calls and proxy information

INTERVIEWER: Does it make sense to make continued call attempts to reach respondent [respname]?

- ◯ Yes
- ◯ No

INTERVIEWER: Proceed and ask informant the below questions about deceased, sick/hospitalized or absent/moved respondents

Proxy Interview with Informant

VASY1_intro: If you permit, I would like to ask you some questions about [respname]'s health prior to his/her death? If you don't know or don't have any information, simply let me know.

Questions about Deceased Respondents

VASY1a: When did [respname] die?

Year of death

- ◯ 2020
- ◯ 2019
- ◯ 2018
- ◯ 2017
- ◯ 2016
- ◯ 2015
- ◯ Prior to 2015
- ◯ Refuse to answer
- ◯ Don't know / don't recall

Month of death

- ◯ January
- ◯ February
- ◯ March
- ◯ April
- ◯ May
- ◯ June
- ◯ July
- ◯ August
- ◯ September
- ◯ October
- ◯ November
- ◯ December
- ◯ Don't know / don't recall

Day of Death	◯ 1<br>◯ 2<br>◯ 3<br>◯ 4<br>◯ 5<br>◯ 6<br>◯ 7<br>◯ 8<br>◯ 9<br>◯ 10<br>◯ 11<br>◯ 12<br>◯ 13<br>◯ 14<br>◯ 15<br>◯ 16<br>◯ 17<br>◯ 18<br>◯ 19<br>◯ 20<br>◯ 21<br>◯ 22<br>◯ 23<br>◯ 24<br>◯ 25<br>◯ 26<br>◯ 27<br>◯ 28<br>◯ 29<br>◯ 30<br>◯ 31<br>◯ Don't know / don't recall
How long ago did [respname] die?	◯ Less than 1 month ago<br>◯ 1-3 months ago<br>◯ 4-6 months ago<br>◯ 7-12 months ago<br>◯ More than one year ago<br>◯ Don't know / don't recall
VASY2a: Prior to his/her death, did [respname] have a fever?	◯ Yes<br>◯ No<br>◯ Don't know / don't remember
VASY2b: When did it start?	◯ less than 1 day before his/her death<br>◯ 1-2 days before his/her death<br>◯ 3-6 days before his/her death<br>◯ about 1 week before his/her death<br>◯ about 2 weeks before his/her death<br>◯ about 3 weeks before his/her death<br>◯ about 1 month before his/her death<br>◯ more than 1 month before his/her death<br>◯ Don't know / don't remember
VASY2c: Prior to his/her death, did [respname] have a dry cough?	◯ Yes<br>◯ No<br>◯ Don't know / don't remember

VASY2d: When did it start?

- ◯ less than 1 day before his/her death
- ◯ 1-2 days before his/her death
- ◯ 3-6 days before his/her death
- ◯ about 1 week before his/her death
- ◯ about 2 weeks before his/her death
- ◯ about 3 weeks before his/her death
- ◯ about 1 month before his/her death
- ◯ more than 1 month before his/her death
- ◯ Don't know / don't remember

VASY2e: Prior to his/her death, did [respname] have shortness of breath, or have difficulties breathing?

- ◯ Yes
- ◯ No
- ◯ Don't know / don't remember

VASY2f: When did it start?

- ◯ less than 1 day before his/her death
- ◯ 1-2 days before his/her death
- ◯ 3-6 days before his/her death
- ◯ about 1 week before his/her death
- ◯ about 2 weeks before his/her death
- ◯ about 3 weeks before his/her death
- ◯ about 1 month before his/her death
- ◯ more than 1 month before his/her death
- ◯ Don't know / don't remember

VASY2g: How likely do you think that [respname] had coronavirus (Covid-19) when he/she died?

- ◯ Very likely
- ◯ Likely
- ◯ Not likely
- ◯ Very unlikely
- ◯ Don't know /refuse to answer

(Read Responses)

VASY4a: Has [respname] sought medical care prior to his/her death?

- ◯ Yes
- ◯ No
- ◯ Don't know / don't remember

VASY4b: Did a doctor, health care provider or traditional healer tell [respname] that he/she had coronavirus (Covid-19)?

- ◯ Yes
- ◯ No
- ◯ Don't know / don't remember

VASY4c: Was this based on a test for coronavirus (Covid-19)?

- ◯ Yes
- ◯ No
- ◯ Don't know / don't remember

Questions about Sick, Hospitalized or Absent/Moved Respondents

PRXYSY1_intro: Since [respname] is not available right now for this phone survey, if you permit, I would like to ask you some questions about [respname]'s health? If you don't know or don't have any information, simply let me know.

PXYSY1a: How long has [respname] been sick?

- ◯ less than 1 day
- ◯ 1-2 days
- ◯ 3-6 days
- ◯ about 1 week
- ◯ about 2 weeks
- ◯ about 3 weeks
- ◯ about 1 month
- ◯ more than 1 month
- ◯ Don't know / don't recall

PXYSY1b: How long has [respname] been hospitalized?	◯ less than 1 day<br>◯ 1-2 days<br>◯ 3-6 days<br>◯ about 1 week<br>◯ about 2 weeks<br>◯ about 3 weeks<br>◯ about 1 month<br>◯ more than 1 month<br>◯ Don't know / don't recall
PXYSY1c: How long has [respname] been away?	◯ about 1 week<br>◯ about 2 weeks<br>◯ about 3 weeks<br>◯ about 1 month<br>◯ about 2 months<br>◯ about 3 months<br>◯ more than 3 months<br>◯ Don't remember
PXYSY2a: Does [respname] currently have a fever?	◯ Yes<br>◯ No<br>◯ Don't know / don't remember
PXYSY2b: When did it start?	◯ less than 1 day ago<br>◯ 1-2 days ago<br>◯ 3-6 days ago<br>◯ about 1 week ago<br>◯ about 2 weeks ago<br>◯ about 3 weeks ago<br>◯ about 1 month ago<br>◯ more than 1 month ago<br>◯ Don't remember
PXYSY2c: Does [respname] currently have a dry cough?	◯ Yes<br>◯ No<br>◯ Don't know / don't remember
PXYSY2d: When did it start?	◯ less than 1 day ago<br>◯ 1-2 days ago<br>◯ 3-6 days ago<br>◯ about 1 week ago<br>◯ about 2 weeks ago<br>◯ about 3 weeks ago<br>◯ about 1 month ago<br>◯ more than 1 month ago<br>◯ Don't remember
PXYSY2e: Does [respname] currently have shortness of breath, or have difficulties breathing?	◯ Yes<br>◯ No<br>◯ Don't know / don't remember
PXYSY2f: When did it start?	◯ less than 1 day ago<br>◯ 1-2 days ago<br>◯ 3-6 days ago<br>◯ about 1 week ago<br>◯ about 2 weeks ago<br>◯ about 3 weeks ago<br>◯ about 1 month ago<br>◯ more than 1 month ago<br>◯ Don't remember

PXYSY2g: How likely do you think that [respname] currently has coronavirus (Covid-19) ?	◯ Very likely<br>◯ Likely<br>◯ Not likely<br>◯ Very unlikely<br>◯ Don't know /refuse to answer
PXYSY3a: Did [respname] have any of these symptoms (fever, dry cough or shortness of breath) in the last few months?	◯ Yes<br>◯ No<br>◯ Don't know / don't remember
PXYSY3b: When?	◯ about 1 week ago<br>◯ about 2 weeks ago<br>◯ about 3 weeks ago<br>◯ about 1 month ago<br>◯ about 2 months ago<br>◯ about 3 months ago<br>◯ more than 3 months ago<br>◯ Don't remember
PXYSY3c: At that time, did [respname] have fever, dry cough and shortness of breath at the same time?	◯ Yes<br>◯ No<br>◯ Don't know / don't remember
PXYSY3d: How likely do you think it is that [respname] had coronavirus (Covid-19) at that time?	◯ Very likely<br>◯ Likely<br>◯ Not likely<br>◯ Very unlikely<br>◯ Don't know /refuse to answer<br>(Read Responses)
PXYSY4a: Has [respname] sought medical care?	◯ Yes<br>◯ No<br>◯ Don't know / don't remember
PXYSY4b: Did a doctor, health care provider or traditional healer tell [respname] that he/she had coronavirus (Covid-19)?	◯ Yes<br>◯ No<br>◯ Don't know / don't remember
PXYSY4c: Was this based on a test for coronavirus (Covid-19)?	◯ Yes<br>◯ No<br>◯ Don't know / don't remember

Informant Data

Finally, I'd like to ask some questions about yourself

What is your First Name?	______________________________
What is your Last Name?	______________________________

What is your relationship to [respname]?	◯ Wife/husband<br>◯ Son/daughter<br>◯ Father/mother<br>◯ Grandchild<br>◯ Grandparent<br>◯ Mother/father-in-law<br>◯ Son/daughter-in-law<br>◯ Brother/sister-in-law<br>◯ Paternal aunt/uncle<br>◯ Maternal aunt/uncle<br>◯ Sister/brother<br>◯ Cousin<br>◯ Nephew/niece<br>◯ Step-child/half-brother/sister<br>◯ Co-wife<br>◯ Boyfriend/Girlfriend, including PTM<br>◯ Other not related through blood or marriage (friends, neighbors, etc)<br>◯ Step-mother/step-father<br>◯ Don't know
How well did you know [respname]?	◯ Very well<br>◯ Quite well<br>◯ By name only
Are you......	◯ Living in the same HH as Respondent<br>◯ Living in the same compound/different household<br>◯ Friend/neighbor<br>◯ Other
Specify:	__________________________

End of Proxy Interview

Time Proxy Interview ended:	__________________________<br>(Day Month Year Hours:Min, entered as 15-04-2018 17:45)

INTERVIEWER: Thank the informant: Thank you for your participation in this study.

INTERVIEWER: End of Informant Interview (do not proceed to other parts of the survey; hang up and complete Supervisor Quality Check)

Cv1 2a Informed Consent

Time

____

Respondent

[respname] (RespID: [respid])
([gender], about [age] years old, [maritalstatus] at last survey; village: [villagename], region: [region], previous survey: [prior_surv_year], conducted in [prior_surv_language])

Informed Consent

Date/Time of Informed Consent

____
(Day Month Year Hours:Min, entered as 15-04-2018 17:45)

Hello, I am [cv1_ivername] from Invest in Knowledge (IKI). I am calling you because you participated in the last three years in studies conducted by the Malawi Longitudinal Study of Families and Health (MLSFH), also known as MDICP or Let's Chat in your community.

Today, we are asking you to participate in a short follow-up interview about current health problems in Malawi, and about some behaviors and perceptions related to your health. We would also like to call you again in the future, if you agree.

Today's interview will last approximately 15 to 30 minutes. There will be no penalty if you decide not to participate. There will be no penalty if you decide to drop out after today's interview, or after any of the future interviews.

If you agree, I will ask questions about yourself as well as about your health, economic conditions, social contexts, and the health of your family members. I will also ask you questions about your knowledge about a new health issue, the novel Coronavirus (or COVID19), and how this new disease is affecting you and your family.

You may at times feel uncomfortable or sadness answering some questions. You may skip any questions you want or take time thinking about your responses.

About 2,000 respondents of the Malawi Longitudinal Study of Families and Health (MLSFH) will participate in this study.

We hope that the information we gain from this research will be used to develop and improve programs to help improve the health and well-being of individuals in Malawi, especially with regard to Corona (Covid-19).

All of the information we collect in this study will be strictly confidential, and will only be used for research purposes.

The data we collect today as part of this study will be linked to earlier information that you have provided as part of your participation in the Malawi Longitudinal Study of Families and Health (MLSFH), as well as contextual information about the MLSFH study areas.

The MLSFH study team will also retain your name and other identifying information to allow you to participate again in future data collections as part of the MLSFH. The data collected as part of this study will become part of the MLSFH data archive and will be stored indefinitely.

These data will be analyzed using statistical methods in order to (a) document how economic conditions, social contexts, health conditions and family structures in Malawi have changed in recent times, and (b) understand how some individuals, families and communities have been able to be more resilient than others during these difficult times.

The main risk to you if you decide to participate in this study is the chance of a breach of confidentiality. All reasonable steps will be taken to ensure that your identity will be concealed and that you are protected from the risk of a breach of confidentiality.

First, the interview will take place entirely by phone, so that no one can overhear our conversation with you.

Second, we will record the information using tablets, which are protected by a password. Only authorized study team members can see it.

Third, your name and any other information that might be used to identify you will be removed from data that we release to other researchers. All information about the names of other persons that you mention, or their health and characteristics, will remain completely confidential.

The MLSFH data and information that is released as part of our research will not allow other persons to identify you, your family or any other individuals. If any publication or presentations result from this research, you, your family and/or your village/community will not be identified by name. The risk of a breach of confidentiality is very small given these data protection steps implemented as part of this study.

Upon completing today's interview, you will be provided with 1,000 Malawian Kwachas in airtime via your mobile phone. It may take a few days until this airtime is transfered to you. You will receive the same amount if we call you back for another survey.

If you have any questions pertaining to your participation in this research study, or if you have any questions about your rights as a research participant or concerns or complaints about this study, please contact Mr. James Mwera (Tel: 0999925707), who is our local study director working for Invest in Knowledge (IKI) in Malawi.

Additionally, you can call or contact National Health Science Research Committee (NHSRC) Director, Dr. Collins Mitambo, on 0999397913 or 01789400 if you have questions about your rights as a study participant. Contact the NHSRC if you feel you have not been treated fairly or if you have other concerns. The NHSRC contact information is: Malawi National Health Sciences Research Committeem Ministry of Health, Phone: 01 726 422/418

Do you have any questions?

Do you agree to participate in this MLFH Phone Survey: Covid-19 in Malawi?	◯ Yes<br>◯ No
Number of consent questions answered	__________________

Respondent did NOT give consent to participate in this MLSFH Phone Survey "Covid-19 in Malawi"

Interviewer: Prompt if respondent has any questions about this study and his/her participation in this study. If yes, attempt to clarify respondent's question/concerns as much as possible. Then ask again:	◯ Mark if respondent asked additional questions about this study
Do you agree to participate in this MLFH Phone Survey: Covid-19 in Malawi?	◯ Yes<br>◯ No

---

Respondent did NOT give consent to participate in this MLSFH Phone Survey "Covid-19 in Malawi"

DO NOT PROCEED WITH THIS STUDY

Consent: Study Participation (1+2 combined)	__________________

Summary of Informed Consent for [respname] ([respid]):

Consent has been granted for:

-- MLFH Phone Survey: Covid-19 in Malawi

Interviewer: Proceed with phone survey

Cv1 3a Background

Time

____

Respondent

[respname] (RespID: [respid])
([gender], about [age] years old, [maritalstatus] at last survey; village: [villagename], region: [region], previous survey: [prior_surv_year], conducted in [prior_surv_language])

R1: Are you still residing in [villagename] (Region: [region])
- ◯ Yes
- ◯ No

R2: Where do you currently reside?

R2a: Village name:

____

R2b: District:
- ◯ Mchinji
- ◯ Balaka
- ◯ Rumphi
- ◯ Dedza
- ◯ Dowa
- ◯ Kasungu
- ◯ Lilongwe
- ◯ Nkhotakota
- ◯ Ntcheu
- ◯ Ntchisi
- ◯ Salima
- ◯ Chitipa
- ◯ Karonga
- ◯ Likoma
- ◯ Mzimba
- ◯ Nkhata Bay
- ◯ Blantyre
- ◯ Chikwawa
- ◯ Chiradzulu
- ◯ Machinga
- ◯ Mangochi
- ◯ Mulanje
- ◯ Mwanza
- ◯ Nsanje
- ◯ Thyolo
- ◯ Phalombe
- ◯ Zomba
- ◯ Neno

R2c: Is this residence in an urban or rural area?
- ◯ Urban
- ◯ Peri-urban
- ◯ Rural

R2d: When did you move to this location?
- ◯ Less than 1 month ago
- ◯ 1-3 months ago
- ◯ 4-6 months ago
- ◯ 7-12 months ago
- ◯ More than one year ago
- ◯ Don't know

Background Questions

M1: What is your present marital status?

- ◯ Married/living together
- ◯ Separated
- ◯ Divorced
- ◯ Widowed
- ◯ Never married

B4_note: Now I would like to ask you some questions about your health and well being.

B4: I am interested in your general level of well-being or satisfaction with life. How satisfied are you with your life, all things considered?

- ◯ Very satisfied
- ◯ Somewhat satisfied
- ◯ Satisfied
- ◯ Somewhat unsatisfied
- ◯ Very unsatisfied

B6: In general, would you say your health now is:

- ◯ Excellent
- ◯ Very good
- ◯ Good
- ◯ Fair
- ◯ Poor
- ◯ Don't know

E0a: All things considered, in the last year (12 months), did your economic situation improve or worsen?

- ◯ Improved a lot
- ◯ Improved somewhat
- ◯ Stayed somewhat the same
- ◯ Worsened somewhat
- ◯ Worsened a lot
- ◯ Don't Know

Mental Health

CPH1_note: Over the last 2 weeks (14 days), how often have you been bothered by any of the following problems? INTERVIEWER: Read responses for each question and remind respondent of the two week time frame for each question.

CPH1: Little interest or pleasure in doing things?

- ◯ Not at all
- ◯ Several days
- ◯ More than half of the days
- ◯ Nearly everyday

(Read Responses)

CPH2: Feeling down, depressed, or hopeless?

- ◯ Not at all
- ◯ Several days
- ◯ More than half of the days
- ◯ Nearly everyday

(Read Responses)

CPH3: Trouble falling or staying asleep, or sleeping too much?

- ◯ Not at all
- ◯ Several days
- ◯ More than half of the days
- ◯ Nearly everyday

(Read Responses)

CPH4: Feeling tired or having little energy?

- ◯ Not at all
- ◯ Several days
- ◯ More than half of the days
- ◯ Nearly everyday

(Read Responses)

CPH5: Poor appetite or overeating?	◯ Not at all<br>◯ Several days<br>◯ More than half of the days<br>◯ Nearly everyday<br>(Read Responses)
CPH6: Feeling bad about yourself, being failure or have let yourself/family down	◯ Not at all<br>◯ Several days<br>◯ More than half of the days<br>◯ Nearly everyday<br>(Read Responses)
CPH7: Trouble concentrating on things such as farming, chatting with friends, etc	◯ Not at all<br>◯ Several days<br>◯ More than half of the days<br>◯ Nearly everyday<br>(Read Responses)
CPH8: Moving/speaking slowly, or being fidgety/restless?	◯ Not at all<br>◯ Several days<br>◯ More than half of the days<br>◯ Nearly everyday<br>(Read Responses)
CPH9: Thoughts that you would be better off dead or of hurting yourself.	◯ Not at all<br>◯ Several days<br>◯ More than half of the days<br>◯ Nearly everyday<br>(Read Responses)

Health Care Utilization

HU2: Over the past month, has there been a time when (you felt) you needed medical care but did not seek it or could not get it?	◯ Yes<br>◯ No

HU3: Reasons why you did not get health care when you needed it last time?
INTERVIEWER: Do NOT read responses. Check all that apply. Probe if NO response

a: Could not afford the cost of the visit	◯ Check if mentioned by resp.<br>◯ Not mentioned
b: No transport available	◯ Check if mentioned by resp.<br>◯ Not mentioned
c: Could not afford the cost of transport	◯ Check if mentioned by resp.<br>◯ Not mentioned
d: I was previously badly treated	◯ Check if mentioned by resp.<br>◯ Not mentioned
e: Could not take time off work or had other commitments	◯ Check if mentioned by resp.<br>◯ Not mentioned
f: The health care provider's drugs or equipment were inadequate	◯ Check if mentioned by resp.<br>◯ Not mentioned

g: The health care provider's skills were inadequate	◯ Check if mentioned by resp.<br>◯ Not mentioned
h: I did not know where to go	◯ Check if mentioned by resp.<br>◯ Not mentioned
i: I tried but were denied health care	◯ Check if mentioned by resp.<br>◯ Not mentioned
j: I thought I was not sick enough	◯ Check if mentioned by resp.<br>◯ Not mentioned
k: Health care facility is too far away	◯ Check if mentioned by resp.<br>◯ Not mentioned
l: Health care facility is not accessible for me	◯ Check if mentioned by resp.<br>◯ Not mentioned
m: Afraid of becoming infected with coronavirus (Covid-19)	◯ Check if mentioned by resp.<br>◯ Not mentioned
n: Other	◯ Check if mentioned by resp.<br>◯ Not mentioned
o: Don't know / don't remember	◯ Check if mentioned by resp.<br>◯ Not mentioned

Response "Don't know / don't remember" can not be combined with any other response option; check either "None of the above" or any of the other options for this question

Cv1 3b Knowledge Behaviors

Time ______________________

Respondent

[respname] (RespID: [respid])
([gender], about [age] years old; village: [villagename], region: [region], previous survey: [prior_surv_year], conducted in [prior_surv_language])

Knowledge about Covid-19 and Risk Reduction Strategies

CK1a: Have you heard of the coronavirus (COVID-19)?
- ◯ Yes
- ◯ No

CK1b: Let me try again: Have you heard about a new disease, called coronavirus or Covid-19 that is affecting individuals in Malawi and elsewhere?
- ◯ Yes
- ◯ No

CK1_info: Since you have not heard about coronavirus, also called Covid-19, let me give you some information. Coronavirus, or Covid-19, is a new disease that is spread from person to person similar to the flu. The President of Malawi has declared a State of Emergency due to the Coronavirus, and has urged Malawians to take necessary precautions to protect themselves.

Ask Covid-19 related questions? ______________________

CK2: Can you tell me some of the symptoms of having coronavirus or Covid 19?
INTERVIEWER: Do NOT read responses; mark Yes if a symptom is mentioned by Resp, No otherwise

a: Fever
- ◯ Yes
- ◯ No

b: Cough
- ◯ Yes
- ◯ No

c: Shortness of breath
- ◯ Yes
- ◯ No

d: Sore throat
- ◯ Yes
- ◯ No

e: Runny or stuffy nose
- ◯ Yes
- ◯ No

f: Muscle or body aches
- ◯ Yes
- ◯ No

g: Headaches
- ◯ Yes
- ◯ No

h: Fatigue (tiredness)
- ◯ Yes
- ◯ No

i: Diarrhea	◯ Yes<br>◯ No
j: Loss of taste and/or smell	◯ Yes<br>◯ No
Count of recorded answers	______________________

INTERVIEWER: Make sure you recorded an answer (Yes or No) for each of the symptoms above

Agreement with Statements about Covid-19

CK3: I will read some statements about Covid 19. Tell me after each statement if you agree or disagree.

b: People can be infected and show no symptoms	◯ Agree<br>◯ Disagree
c: Only people with symptoms are contagious	◯ Agree<br>◯ Disagree
d: You can become infected by shaking hands	◯ Agree<br>◯ Disagree
e: You can become infected by close contact with people even if you are not touching	◯ Agree<br>◯ Disagree
h: Once you are infected, you are infected forever	◯ Agree<br>◯ Disagree
i: Most people recover in less than a month	◯ Agree<br>◯ Disagree
j: There is currently no effective treatment for people who have COVID-19	◯ Agree<br>◯ Disagree
k: The elderly are more likely to become severely ill from COVID-19	◯ Agree<br>◯ Disagree
m: It is possible to get corona virus (COVID-19) from drinking unfiltered water	◯ Agree<br>◯ Disagree
o: Corona virus (COVID-19) spreads from one person to another through respiratory droplets	◯ Agree<br>◯ Disagree
p: It is possible to get infected by touching an object or surface (e.g., table, door handle or water pump)	◯ Agree<br>◯ Disagree

INTERVIEWER: Make sure you recorded an answer (Yes or No) for each of the symptoms above

Covid-19 Prevention Strategies

CK4: Which of the following actions can reduce the risk of being infected with corona virus (Covid-19)?
INTERVIEWER: Read all options; mark Yes if a preventive action is mentioned by Resp, No otherwise

a: Using herbs	◯ Yes<br>◯ No
b: Washing hands with soap and water frequently	◯ Yes<br>◯ No
c: Avoiding any close contact (2 meters) with people when you go out	◯ Yes<br>◯ No
g: Wearing something that covers your mouth and nose when you go out	◯ Yes<br>◯ No
h: Avoiding shaking hands with others	◯ Yes<br>◯ No
i: Coughing/sneezing into your elbow	◯ Yes<br>◯ No
j: Prayer	◯ Yes<br>◯ No
k: Other	◯ Yes<br>◯ No

INTERVIEWER: Make sure you recorded an answer (Yes or No) for each of the options above

HW1a: Do you have a place to wash your hands at your current residence?	◯ Yes<br>◯ No
HW1b: Do you have soap at this place to wash your hands?	◯ Yes<br>◯ No
HW2: How many times per day do you wash your hands with soap?	◯ Not at all (zero times)<br>◯ Once (1x)<br>◯ Twice (2x)<br>◯ 3 times<br>◯ 4 times<br>◯ 5 times,<br>◯ 6-9 times<br>◯ 10 times or more<br>◯ Don't know / don't remember

The next questions are about your ability to reduce social contacts to persons outside of your home. Tell me after each statement if you agree or disagree.

SD1: It is impossible or very difficult to avoid social contacts ...

c: ... because you frequently need to visit your extended family	◯ Agree<br>◯ Disagree
d: ... because you are obliged to go to funerals in the community	◯ Agree<br>◯ Disagree

e: ... because you regularly need to attend religious services

◯ Agree
◯ Disagree

Ability to Reduce Infection Risks

SD2a: Do you or your household have any face masks (purchased, received or self-made)?

◯ Yes
◯ No
(Refers to household; includes scarves or similar if used as mask)

SD2b: How many does your household have?

◯ Zero (none)
◯ 1
◯ 2
◯ 3
◯ 4
◯ 5
◯ 6
◯ 7
◯ 8
◯ 9
◯ 10
◯ 11
◯ 12
◯ 13
◯ 14
◯ 15-19
◯ 20-24
◯ 25-29
◯ 30-34
◯ 35-39
◯ 40-44
◯ 45-49
◯ 50 or more
("how many" refers to household)

SD3: In case you were to become sick with corona virus (Covid-19), do you have an ability to separate yourself for 1-2 weeks from other members in your household?

◯ Yes
◯ No

Social Distancing and Prevention Behaviors

SD4a: In the last day, how many hours did you spend close to persons not living in your household?

◯ No time at all (zero hours)
◯ Less than 1/2 hour
◯ Less than 1 hour
◯ about 1 hour
◯ about 1 hour 30 minutes
◯ about 2 hours
◯ about 2.5 hours
◯ about 3 hours
◯ about 3.5 hours
◯ about 4 hours
◯ about 4.5 hours
◯ about 5 hours
◯ about 5.5 hours
◯ about 6 hours
◯ More than 6 hours

SD4b: Has the time you spent close to persons not living in your household changed since beginning of the coronavirus epidemic in Malawi?	◯ Yes, time increased<br>◯ Yes, time decreased<br>◯ No change

SD5: Have you taken any other action to prevent becoming infected, and if so, what have you done?
INTERVIEWER: Read all options; mark Yes if a symptom is mentioned by Resp, No otherwise

a: Using herbs	◯ Yes<br>◯ No
b: Washing hands with soap and water frequently	◯ Yes<br>◯ No
c: Avoiding any close contact (2 meters) with people when you go out	◯ Yes<br>◯ No
d: Staying in your home	◯ Yes<br>◯ No
e: Getting vaccinated	◯ Yes<br>◯ No
f: Traditional practices	◯ Yes<br>◯ No
g: Wearing something that covers your mouth and nose when you go out	◯ Yes<br>◯ No
h: Avoiding shaking hands with others	◯ Yes<br>◯ No
i: Coughing/sneezing into your elbow	◯ Yes<br>◯ No
j: Prayer	◯ Yes<br>◯ No
k: Other	◯ Yes<br>◯ No

INTERVIEWER: Make sure you recorded an answer (Yes or No) for each of the options above

Other Relevant Behaviors

I would like to ask you some additional questions about other important aspects of life.

Ask family planning questions? (Men younger than 60, women younger than 50)	__________________________________
F2: Are you currently pregnant?	◯ Yes<br>◯ No<br>◯ Don't know

F2: Is your spouse/partner currently pregnant?	◯ Yes<br>◯ No<br>◯ Don't know
F4: Would you like to have a child (another child), or would you like to stop having children?	◯ Have a(nother) child<br>◯ Stop, no more<br>◯ Husband deceased/left<br>◯ Says can't get pregnant<br>◯ Too old<br>◯ Don't know
F4: After the child you are expecting is born, would you like to have another child or would you like to stop having children?	◯ Have a(nother) child<br>◯ Stop, no more<br>◯ Husband deceased/left<br>◯ Says can't get pregnant<br>◯ Too old<br>◯ Don't know
F4: Would you like to have a child (another child), or would you like to stop having children?	◯ Have a(nother) child<br>◯ Stop, no more<br>◯ Wife deceased/left<br>◯ (Wife) Says can't get pregnant<br>◯ Too old<br>◯ Don't know
F4: After the child you are expecting is born, would you like to have another child or would you like to stop having children?	◯ Have a(nother) child<br>◯ Stop, no more<br>◯ Wife deceased/left<br>◯ (Wife) Says can't get pregnant<br>◯ Too old<br>◯ Don't know
F5: How long would you like to wait before having a(nother) child?	◯ As soon as possible<br>◯ Less than 2 years<br>◯ 2 years or more<br>◯ No preference/whenever<br>◯ Don't know
OB2a: In the last week, did your spouse insult you or made you feel bad about yourself?	◯ Yes<br>◯ No<br>◯ Refuse to answer
OB2b: In the last week, did your spouse push, slap, kick, punch or beat you?	◯ Yes<br>◯ No<br>◯ Refuse to answer
OB2c: In the last week, did you push, slap, kick, punch or beat your spouse?	◯ Yes<br>◯ No<br>◯ Refuse to answer
OB2d: In the last week, did you physically discipline any of your children?	◯ Yes<br>◯ No<br>◯ Does not apply (no children)<br>◯ Refuse to answer
OB3a: Did you have sex in the last week?	◯ Yes<br>◯ No<br>◯ Refuse to answer

OB3b: The last time you had sex, did you use a condom?	◯ Yes<br>◯ No<br>◯ Refuse to answer
OB3c: Was this the case because you could not find/buy one?	◯ Yes<br>◯ No<br>◯ Refuse to answer
CO6_intro: In the last 4 weeks, have you or anyone in your household ...	
CO6: Worried that the household would not have enough food	◯ Never<br>◯ Rarely<br>◯ Sometimes<br>◯ Often<br>(Read Responses)
CO10: Eaten a smaller meal than you felt you needed	◯ Never<br>◯ Rarely<br>◯ Sometimes<br>◯ Often<br>(Read Responses)
CO14: Gone a whole day and night without eating	◯ Never<br>◯ Rarely<br>◯ Sometimes<br>◯ Often<br>(Read Responses)

Cv1 3c Social Networks And Activities

Time ______________________

Respondent

[respname] (RespID: [respid])
([gender], about [age] years old; village: [villagename], region: [region], previous survey: [prior_surv_year], conducted in [prior_surv_language])

Covid-19 Infections and Mortality in Social Network

SN1a: Overall, how many people known to you do you suspect had become infected with coronavirus (Covid-19) in the past 3 months?

- ◯ Nobody (zero persons)
- ◯ 1 Person
- ◯ 2 Persons
- ◯ 3 Persons
- ◯ 4 Persons
- ◯ 5 Persons
- ◯ 6 Persons
- ◯ 7 Persons
- ◯ 8 Persons
- ◯ 9 Persons
- ◯ 10 Persons
- ◯ 11 Persons
- ◯ 12 Persons
- ◯ 13 Persons
- ◯ 14 Persons
- ◯ 15-19 Persons
- ◯ 20-24 Persons
- ◯ 25-29 Persons
- ◯ 30-39 Persons
- ◯ 40-49 Persons
- ◯ 50 or more persons
- ◯ Don't know / don't remember

SN1b: Overall, how many people known to you do you suspect have died from coronavirus (Covid-19) in the past 3 months?

- ◯ Nobody (zero persons)
- ◯ 1 Person
- ◯ 2 Persons
- ◯ 3 Persons
- ◯ 4 Persons
- ◯ 5 Persons
- ◯ 6 Persons
- ◯ 7 Persons
- ◯ 8 Persons
- ◯ 9 Persons
- ◯ 10 Persons
- ◯ 11 Persons
- ◯ 12 Persons
- ◯ 13 Persons
- ◯ 14 Persons
- ◯ 15-19 Persons
- ◯ 20-24 Persons
- ◯ 25-29 Persons
- ◯ 30-39 Persons
- ◯ 40-49 Persons
- ◯ 50 or more persons
- ◯ Don't know / don't remember

INTERVIEWER: Probe -- generally, number infected with Covid-19 should be larger than number who has died from Covid-19

In-Person Social Interactions about Covid-19

SN2: How many persons outside of your household have you talked to in person about coronavirus (Covid-19) during the last week?	◯ Nobody (zero persons)<br>◯ 1 Person<br>◯ 2 Persons<br>◯ 3 Persons<br>◯ 4 Persons<br>◯ 5 Persons<br>◯ 6 Persons<br>◯ 7 Persons<br>◯ 8 Persons<br>◯ 9 Persons<br>◯ 10 Persons<br>◯ 11 Persons<br>◯ 12 Persons<br>◯ 13 Persons<br>◯ 14 Persons<br>◯ 15-19 Persons<br>◯ 20-24 Persons<br>◯ 25-29 Persons<br>◯ 30-39 Persons<br>◯ 40-49 Persons<br>◯ 50 or more persons<br>◯ Don't know / don't remember
SN3: In these conversations, did you generally give information about coronavirus, receive information, or both?	◯ Give information<br>◯ Receive information<br>◯ Both<br>(SN3 and SN4 pertain to in-person conversations with persons outside the household)
SN4: In these conversations with others, did you talk about ...<br>INTERVIEWER: Read responses	
a: ... symptoms of coronavirus (Covid-19)?	◯ Yes<br>◯ No
b: ... ways to protect yourself or your family from getting infected with coronavirus (Covid-19)?	◯ Yes<br>◯ No
c: ... the impact of coronavirus (Covid-19) on your ability to work or earn money?	◯ Yes<br>◯ No
d: ... possibilities on how to get money or help from others during the Covid-19 epidemic?	◯ Yes<br>◯ No<br>(Money or financial help includes cash, loans, informal credit, mobile money and similar means of financial help. Non-financial help includes aspects like collecting firewood, cooking, taking care of people, or helping with farming.)

Phone/Online Social Interactions about Covid-19

SN6a: How many persons did you talk to on the phone about coronavirus (Covid-19) during the last week?	◯ Nobody (zero persons)<br>◯ 1 Person<br>◯ 2 Persons<br>◯ 3 Persons<br>◯ 4 Persons<br>◯ 5 Persons<br>◯ 6 Persons<br>◯ 7 Persons<br>◯ 8 Persons<br>◯ 9 Persons<br>◯ 10 Persons<br>◯ 11 Persons<br>◯ 12 Persons<br>◯ 13 Persons<br>◯ 14 Persons<br>◯ 15-19 Persons<br>◯ 20-24 Persons<br>◯ 25-29 Persons<br>◯ 30-39 Persons<br>◯ 40-49 Persons<br>◯ 50 or more persons<br>◯ Don't know / don't remember
SN6b: Do you have access to a smart phone (own or somebody elses)?	◯ Yes<br>◯ No
SN6c: With how many persons did you interact on WhatsApp or other instant messenger application about coronavirus (Covid-19) during the last week?	◯ Nobody (zero persons)<br>◯ 1 Person<br>◯ 2 Persons<br>◯ 3 Persons<br>◯ 4 Persons<br>◯ 5 Persons<br>◯ 6 Persons<br>◯ 7 Persons<br>◯ 8 Persons<br>◯ 9 Persons<br>◯ 10 Persons<br>◯ 11 Persons<br>◯ 12 Persons<br>◯ 13 Persons<br>◯ 14 Persons<br>◯ 15-19 Persons<br>◯ 20-24 Persons<br>◯ 25-29 Persons<br>◯ 30-39 Persons<br>◯ 40-49 Persons<br>◯ 50 or more persons<br>◯ Don't know / don't remember
SN6e: In the these phone calls and messages with others did you generally give information about coronavirus, receive information, or both?	◯ Give information<br>◯ Receive information<br>◯ Both

SN7: In these phone calls and messages with others, did you talk about ...
INTERVIEWER: Read responses

a: ... symptoms of coronavirus (Covid-19)?	◯ Yes<br>◯ No

b: ... ways to protect yourself or your family from getting infected with coronavirus (Covid-19)?	◯ Yes<br>◯ No
c: ... the impact of coronavirus (Covid-19) on your ability to work or earn money?	◯ Yes<br>◯ No
d: ... possibilities on how to get money or help from others during the Covid-19 epidemic?	◯ Yes<br>◯ No<br>(Money or financial help includes cash, loans, informal credit, mobile money and similar means of financial help. Non-financial help includes aspects like collecting firewood, cooking, taking care of people, or helping with farming.)

Sources of Information

SN8a: Which is the most important source of information you use to keep updated about coronavirus (Covid-19)?	◯ Government authorities / government messages<br>◯ Local health personnel<br>◯ Traditional healers<br>◯ Community leaders (village head, TA)<br>◯ Religious leaders<br>◯ Newspaper<br>◯ Radio<br>◯ TV<br>◯ Internet or websites<br>◯ Social media<br>◯ WhatsApp or other instant messenger groups<br>◯ Conversation with friends or neighbors<br>◯ Conversation with relatives<br>◯ Conversation with acquaintances or strangers<br>◯ Phone hotline<br>◯ Billboards<br>◯ Other<br>(Interviewer: do NOT read responses)
SN8b: Which is the 2nd most important source of information?	◯ Government authorities / government messages<br>◯ Local health personnel<br>◯ Traditional healers<br>◯ Community leaders (village head, TA)<br>◯ Religious leaders<br>◯ Newspaper<br>◯ Radio<br>◯ TV<br>◯ Internet or websites<br>◯ Social media<br>◯ WhatsApp or other instant messenger groups<br>◯ Conversation with friends or neighbors<br>◯ Conversation with relatives<br>◯ Conversation with acquaintances or strangers<br>◯ Phone hotline<br>◯ Billboards<br>◯ Other<br>◯ No other source of information<br>(Interviewer: do NOT read responses)

SN8c: Which is the 3rd most important source of information?

- ◯ Government authorities / government messages
- ◯ Local health personnel
- ◯ Traditional healers
- ◯ Community leaders (village head, TA)
- ◯ Religious leaders
- ◯ Newspaper
- ◯ Radio
- ◯ TV
- ◯ Internet or websites
- ◯ Social media
- ◯ WhatsApp or other instant messenger groups
- ◯ Conversation with friends or neighbors
- ◯ Conversation with relatives
- ◯ Conversation with acquaintances or strangers
- ◯ Phone hotline
- ◯ Billboards
- ◯ Other
- ◯ No other source of information

(Interviewer: do NOT read responses)

INTERVIEWER: Check responses for SN8: Cannot list the same response more than once for Most important, 2nd most important and 3rd most important reason)

Fake News

SN10: Did you receive information about Covid-19 that was fake news or sounded suspicious to you?

- ◯ Yes
- ◯ No

Local Covid-19 Prevalence

SN11a: If we took a group of 10 people from this area - just normal people who you found working in the fields or in homes - how many of them do you think would now have coronavirus (Covid-19)? INTERVIEWER: Note: the question pertains to "now", the current time

- ◯ 0
- ◯ 1
- ◯ 2
- ◯ 3
- ◯ 4
- ◯ 5
- ◯ 6
- ◯ 7
- ◯ 8
- ◯ 9
- ◯ 10
- ◯ Don't know

SN11b: In three (3) months from now?

- ◯ 0
- ◯ 1
- ◯ 2
- ◯ 3
- ◯ 4
- ◯ 5
- ◯ 6
- ◯ 7
- ◯ 8
- ◯ 9
- ◯ 10
- ◯ Don't know

Social Participation

G1: How many times in last month have you been to ...

a: ... a funeral?

- ◯ 0 (zero) times
- ◯ 1 time
- ◯ 2 times
- ◯ 3 times
- ◯ 4 times
- ◯ 5 times
- ◯ 6 times
- ◯ 7 times
- ◯ 8 times
- ◯ 9 times
- ◯ 10 times
- ◯ 11 times
- ◯ 12 times
- ◯ 13 times
- ◯ 14 times
- ◯ 15-19 times
- ◯ 20-24 times
- ◯ 25-29 times
- ◯ 30 or more times

c: ... a beer place?

- ◯ 0 (zero) times
- ◯ 1 time
- ◯ 2 times
- ◯ 3 times
- ◯ 4 times
- ◯ 5 times
- ◯ 6 times
- ◯ 7 times
- ◯ 8 times
- ◯ 9 times
- ◯ 10 times
- ◯ 11 times
- ◯ 12 times
- ◯ 13 times
- ◯ 14 times
- ◯ 15-19 times
- ◯ 20-24 times
- ◯ 25-29 times
- ◯ 30 or more times

e: ... a market?	◯ 0 (zero) times<br>◯ 1 time<br>◯ 2 times<br>◯ 3 times<br>◯ 4 times<br>◯ 5 times<br>◯ 6 times<br>◯ 7 times<br>◯ 8 times<br>◯ 9 times<br>◯ 10 times<br>◯ 11 times<br>◯ 12 times<br>◯ 13 times<br>◯ 14 times<br>◯ 15-19 times<br>◯ 20-24 times<br>◯ 25-29 times<br>◯ 30 or more times
f: ... a political meeting?	◯ 0 (zero) times<br>◯ 1 time<br>◯ 2 times<br>◯ 3 times<br>◯ 4 times<br>◯ 5 times<br>◯ 6 times<br>◯ 7 times<br>◯ 8 times<br>◯ 9 times<br>◯ 10 times<br>◯ 11 times<br>◯ 12 times<br>◯ 13 times<br>◯ 14 times<br>◯ 15-19 times<br>◯ 20-24 times<br>◯ 25-29 times<br>◯ 30 or more times
g: ... a wedding?	◯ 0 (zero) times<br>◯ 1 time<br>◯ 2 times<br>◯ 3 times<br>◯ 4 times<br>◯ 5 times<br>◯ 6 times<br>◯ 7 times<br>◯ 8 times<br>◯ 9 times<br>◯ 10 times<br>◯ 11 times<br>◯ 12 times<br>◯ 13 times<br>◯ 14 times<br>◯ 15-19 times<br>◯ 20-24 times<br>◯ 25-29 times<br>◯ 30 or more times

h: ... a church/mosque?	◯ 0 (zero) times<br>◯ 1 time<br>◯ 2 times<br>◯ 3 times<br>◯ 4 times<br>◯ 5 times<br>◯ 6 times<br>◯ 7 times<br>◯ 8 times<br>◯ 9 times<br>◯ 10 times<br>◯ 11 times<br>◯ 12 times<br>◯ 13 times<br>◯ 14 times<br>◯ 15-19 times<br>◯ 20-24 times<br>◯ 25-29 times<br>◯ 30 or more times

G2: Have you received any instruction from your village head in response to the Covid-19 epidemic about:

a: Canceling village meetings	◯ Yes<br>◯ No
b: Keeping distance from other people while fetching water	◯ Yes<br>◯ No
c: Stopping public works	◯ Yes<br>◯ No
d: Stopping recreational activities, such as soccer on the playground	◯ Yes<br>◯ No
e: Creating a village fund for emergency purposes	◯ Yes<br>◯ No
f: Redistributing resources (food, money, medical supplies) to the most vulnerable members of the village community	◯ Yes<br>◯ No

Cv1 3d Consequences Epidemic

Time ______________________

Respondent

[respname] (RespID: [respid])
([gender], about [age] years old; village: [villagename], region: [region], previous survey: [prior_surv_year], conducted in [prior_surv_language])

Health and Economic Consequences of Covid-19 Epidemic

EC1: To reduce the economic impact of the Covid-19 epidemic on you or your family, have you already done any of the following:

a: Reduce expenditure on schooling	◯ Yes<br>◯ No<br>(Expenditures for schooling includes school fees, books and other learning materials, school uniform, etc)
b: Reduce expenditure in agriculture	◯ Yes<br>◯ No
c: Reduce non-food consumption (transportation, entertainment)	◯ Yes<br>◯ No
d: Reduce food consumption	◯ Yes<br>◯ No
e: Reduce health expenditures	◯ Yes<br>◯ No
f: Sell animals (livestock), such as chicken or goats	◯ Yes<br>◯ No
g: Buy animals (livestock), such as chicken or goats	◯ Yes<br>◯ No
h: Work more hours or more intensively	◯ Yes<br>◯ No
i: Get credit (loan) from a money lender or bank	◯ Yes<br>◯ No
j: Borrow money from relatives or friends	◯ Yes<br>◯ No
k: Have members of your family move away from your household	◯ Yes<br>◯ No
l: Have members of your family move to your household	◯ Yes<br>◯ No

m: Migrate to another location / change residence	◯ Yes<br>◯ No
SP2. In the past 12 months, did you or anyone else in your household participate in ANY Social Cash Transfer Programme?	
1) Yes, myself	◯ Check if mentioned by resp.
2) Yes, someone else in household	◯ Check if mentioned by resp.<br>(Interviewer: If respondent and someone else in the household partcipated, check both.)
3) No	◯ Check if mentioned by resp.
4) Not applicable	◯ Check if mentioned by resp.
888) Don't know	◯ Check if mentioned by resp.
Response "No" or "Not applicable" can not be combined with any other response option; correct response	
SP2c. What was the reason for you/your household to be included in the program?	
1) Ultra-poor and labour constrained	◯ Check if mentioned by resp.
2) Poor and food insecure	◯ Check if mentioned by resp.
3) Female or child (18 yrs and below) headed household	◯ Check if mentioned by resp.
4) Household with dependency ratio of 1 to 4	◯ Check if mentioned by resp.
5) Older person headed household (64+)	◯ Check if mentioned by resp.
6) Household headed by disabled person or chronically ill person	◯ Check if mentioned by resp.
7) Covid-19	◯ Check if mentioned by resp.
777) Other, specify	◯ Check if mentioned by resp.
888) DK	◯ Check if mentioned by resp.
Response "DK" can not be combined with any other response options; correct response	
EC2a: Do you or your household receive money through the Government's Covid-19 emergency cash transfer programme?	◯ Yes<br>◯ No<br>◯ Don't know<br>◯ Refuse<br>(This question is about the program, announced in April 2020, that transfers about 35,000 Kwacha ($40) monthly to eligible households.)

EC2b: Since when do you or your household receive these payments?

- ◯ Since 1 month ago
- ◯ Since 2 months ago
- ◯ since 3 months ago
- ◯ Since 4-6 months ago
- ◯ Since more than six ago
- ◯ Don't know
- ◯ Refuse

EC2c: How much does your household receive every month?

- ◯ 0 (zero) Kwacha
- ◯ less than 5,000 Kwacha
- ◯ about 5,000 Kwacha
- ◯ about 5,500 Kwacha
- ◯ about 6,000 Kwacha
- ◯ about 6,500 Kwacha
- ◯ about 7,000 Kwacha
- ◯ about 7,500 Kwacha
- ◯ about 8,000 Kwacha
- ◯ about 8,500 Kwacha
- ◯ about 9,000 Kwacha
- ◯ about 9,500 Kwacha
- ◯ about 10,000 Kwacha
- ◯ about 11,000 Kwacha
- ◯ about 12,000 Kwacha
- ◯ about 13,000 Kwacha
- ◯ about 14,000 Kwacha
- ◯ about 15,000 Kwacha
- ◯ about 16,000 Kwacha
- ◯ about 17,000 Kwacha
- ◯ about 18,000 Kwacha
- ◯ about 19,000 Kwacha
- ◯ about 20,000 Kwacha
- ◯ about 25,000 Kwacha
- ◯ about 30,000 Kwacha
- ◯ about 35,000 Kwacha
- ◯ about 40,000 Kwacha
- ◯ about 45,000 Kwacha
- ◯ about 50,000 Kwacha
- ◯ More than 50,000 Kwacha
- ◯ Don't know
- ◯ Refuse

EC2d: How likely do you think it is that you or your household will receive money through this program in the future?

- ◯ Very likely
- ◯ Likely
- ◯ Not likely
- ◯ Very unlikely
- ◯ Don't know /refuse to answer

(Read responses)

Concerns about health care

HC1: Because of Covid-19, are you concerned that you might not be able to access health care in the next months for:

a: Treatment for malaria

- ◯ Yes
- ◯ No
- ◯ Not applicable
- ◯ Refuse

b: HIV testing for you or members of your family	◯ Yes<br>◯ No<br>◯ Not applicable<br>◯ Refuse
c: Prenatal or postnatal care	◯ Yes<br>◯ No<br>◯ Not applicable<br>◯ Refuse
c: Prenatal or postnatal care for your spouse	◯ Yes<br>◯ No<br>◯ Not applicable<br>◯ Refuse
d: Vaccinations or healthcare for your children	◯ Yes<br>◯ No<br>◯ Not applicable<br>◯ Refuse
e: Obtaining a contraception (such as an injectable or pill)	◯ Yes<br>◯ No<br>◯ Not applicable<br>◯ Refuse
l: Obtaining a contraception (such as an injectable or pill) for your spouse	◯ Yes<br>◯ No<br>◯ Not applicable<br>◯ Refuse
f: Treatment for noncommunicable diseases such diabetes or hypertension	◯ Yes<br>◯ No<br>◯ Not applicable<br>◯ Refuse
g: Antiretroviral treatment (ART) for you or members of your family	◯ Yes<br>◯ No<br>◯ Not applicable<br>◯ Refuse

Cv1 4a Expectations

Time ______________________

Respondent

[respname] (RespID: [respid])
([gender], about [age] years old; village: [villagename], region: [region], previous survey: [prior_surv_year], conducted in [prior_surv_language])

Covid-19 Expectations

CX0a: Do you remember how we asked you to respond to questions using peanuts the last time we visited you?

- ◯ Yes
- ◯ No

CX0: I will ask you similar questions about the chance or likelihood that certain events are going to happen. Because we are talking on the phone, I cannot give you actual peanuts. Instead, you need to imagine that you have ten peanuts.

As before, I will ask you to tell me the number of peanuts to express what you think the likelihood or chance is of a specific event happening. One peanut represents one chance out of 10. Zero peanuts means you are sure that the event will NOT happen. As you tell me more peanuts, it means that you think the likelihood that the event happens increases. For example, if you tell me one or two peanuts, it means you think the event is not likely to happen but it is still possible. If you tell me 5 peanuts, it means that it is just as likely it happens as it does not happen (fifty-fifty). If you tell me 6 peanuts, it means the event is slightly more likely to happen than not to happen. If you tell me 10 peanuts, it means you are sure the event will happen. There is no right or wrong answer, I just want to know what you think.

Let me give you an example. Imagine that we are playing Bawo. Say, when asked about the chance that you will win, you tell me 7 peanuts. This means that you believe you would win 7 out of 10 games on average if we play for a long time.

I will now ask you some questions about coronavirus (Covid-19), including how likely you will get infected with coronavirus or might die because of corona virus.

We believe that there is nothing bad that will happen to you. But something bad might happen in the near future years to come, even though you prevent it to happen. If you don't want, you can refuse to answer these questions.

CX1: Out of 10, tell me the number of peanuts that reflects how likely you think it is that ...

CX1a: ... you are infected with coronavirus (Covid-19) now?	◯ 0 Gnuts<br>◯ 1/2 Gnut<br>◯ 1 Gnut<br>◯ 1 Gnut and 1/2 Gnut<br>◯ 2 Gnuts<br>◯ 2 Gnuts and 1/2 Gnut<br>◯ 3 Gnuts<br>◯ 3 Gnuts and 1/2 Gnut<br>◯ 4 Gnuts<br>◯ 4 Gnuts and 1/2 Gnut<br>◯ 5 Gnuts<br>◯ 5 Gnuts and 1/2 Gnut<br>◯ 6 Gnuts<br>◯ 6 Gnuts and 1/2 Gnut<br>◯ 7 Gnuts<br>◯ 7 Gnuts and 1/2 Gnut<br>◯ 8 Gnuts<br>◯ 8 Gnuts and 1/2 Gnut<br>◯ 9 Gnuts<br>◯ 9 Gnuts and 1/2 Gnut<br>◯ 10 Gnuts<br>◯ Don't know / Refuse to answer
CX1b: ... you will be infected with coronavirus (Covid-19) three (3) months from now?	◯ 0 Gnuts<br>◯ 1/2 Gnut<br>◯ 1 Gnut<br>◯ 1 Gnut and 1/2 Gnut<br>◯ 2 Gnuts<br>◯ 2 Gnuts and 1/2 Gnut<br>◯ 3 Gnuts<br>◯ 3 Gnuts and 1/2 Gnut<br>◯ 4 Gnuts<br>◯ 4 Gnuts and 1/2 Gnut<br>◯ 5 Gnuts<br>◯ 5 Gnuts and 1/2 Gnut<br>◯ 6 Gnuts<br>◯ 6 Gnuts and 1/2 Gnut<br>◯ 7 Gnuts<br>◯ 7 Gnuts and 1/2 Gnut<br>◯ 8 Gnuts<br>◯ 8 Gnuts and 1/2 Gnut<br>◯ 9 Gnuts<br>◯ 9 Gnuts and 1/2 Gnut<br>◯ 10 Gnuts<br>◯ Don't know / Refuse to answer<br>((Any number of peanuts is possible, irrespective of prior answers))

CX2: Out of 10, tell me the number of peanuts that reflects how likely you think it is that ...

CX2a: ... you will become severely ill (unable to work) in case you were to become infected with coronavirus (Covid-19)?	◯ 0 Gnuts<br>◯ 1/2 Gnut<br>◯ 1 Gnut<br>◯ 1 Gnut and 1/2 Gnut<br>◯ 2 Gnuts<br>◯ 2 Gnuts and 1/2 Gnut<br>◯ 3 Gnuts<br>◯ 3 Gnuts and 1/2 Gnut<br>◯ 4 Gnuts<br>◯ 4 Gnuts and 1/2 Gnut<br>◯ 5 Gnuts<br>◯ 5 Gnuts and 1/2 Gnut<br>◯ 6 Gnuts<br>◯ 6 Gnuts and 1/2 Gnut<br>◯ 7 Gnuts<br>◯ 7 Gnuts and 1/2 Gnut<br>◯ 8 Gnuts<br>◯ 8 Gnuts and 1/2 Gnut<br>◯ 9 Gnuts<br>◯ 9 Gnuts and 1/2 Gnut<br>◯ 10 Gnuts<br>◯ Don't know / Refuse to answer<br>((Any number of peanuts is possible, irrespective of prior answers))
CX2b: ... you would die of coronavirus in case you were to become infected?	◯ 0 Gnuts<br>◯ 1/2 Gnut<br>◯ 1 Gnut<br>◯ 1 Gnut and 1/2 Gnut<br>◯ 2 Gnuts<br>◯ 2 Gnuts and 1/2 Gnut<br>◯ 3 Gnuts<br>◯ 3 Gnuts and 1/2 Gnut<br>◯ 4 Gnuts<br>◯ 4 Gnuts and 1/2 Gnut<br>◯ 5 Gnuts<br>◯ 5 Gnuts and 1/2 Gnut<br>◯ 6 Gnuts<br>◯ 6 Gnuts and 1/2 Gnut<br>◯ 7 Gnuts<br>◯ 7 Gnuts and 1/2 Gnut<br>◯ 8 Gnuts<br>◯ 8 Gnuts and 1/2 Gnut<br>◯ 9 Gnuts<br>◯ 9 Gnuts and 1/2 Gnut<br>◯ 10 Gnuts<br>◯ Don't know / Refuse to answer<br>((Any number of peanuts is possible, irrespective of prior answers))

CX2c: ... you or your family will experience a shortage of food in the next six months?

◯ 0 Gnuts
◯ 1/2 Gnut
◯ 1 Gnut
◯ 1 Gnut and 1/2 Gnut
◯ 2 Gnuts
◯ 2 Gnuts and 1/2 Gnut
◯ 3 Gnuts
◯ 3 Gnuts and 1/2 Gnut
◯ 4 Gnuts
◯ 4 Gnuts and 1/2 Gnut
◯ 5 Gnuts
◯ 5 Gnuts and 1/2 Gnut
◯ 6 Gnuts
◯ 6 Gnuts and 1/2 Gnut
◯ 7 Gnuts
◯ 7 Gnuts and 1/2 Gnut
◯ 8 Gnuts
◯ 8 Gnuts and 1/2 Gnut
◯ 9 Gnuts
◯ 9 Gnuts and 1/2 Gnut
◯ 10 Gnuts
◯ Don't know / Refuse to answer
((Any number of peanuts is possible, irrespective of prior answers))

CX3: Next, out of 10, tell me the number of peanuts that reflects how likely you think it is that ...

CX3a: ... you will become infected with coronavirus within one week if someone living with you becomes infected with coronavirus?

◯ 0 Gnuts
◯ 1/2 Gnut
◯ 1 Gnut
◯ 1 Gnut and 1/2 Gnut
◯ 2 Gnuts
◯ 2 Gnuts and 1/2 Gnut
◯ 3 Gnuts
◯ 3 Gnuts and 1/2 Gnut
◯ 4 Gnuts
◯ 4 Gnuts and 1/2 Gnut
◯ 5 Gnuts
◯ 5 Gnuts and 1/2 Gnut
◯ 6 Gnuts
◯ 6 Gnuts and 1/2 Gnut
◯ 7 Gnuts
◯ 7 Gnuts and 1/2 Gnut
◯ 8 Gnuts
◯ 8 Gnuts and 1/2 Gnut
◯ 9 Gnuts
◯ 9 Gnuts and 1/2 Gnut
◯ 10 Gnuts
◯ Don't know / Refuse to answer
((Any number of peanuts is possible, irrespective of prior answers))

CX3b: ... you will become infected with coronavirus if you have a 15 minute face-to-face conversation (without face mask) with somebody who is infected with coronavirus?

- ◯ 0 Gnuts
- ◯ 1/2 Gnut
- ◯ 1 Gnut
- ◯ 1 Gnut and 1/2 Gnut
- ◯ 2 Gnuts
- ◯ 2 Gnuts and 1/2 Gnut
- ◯ 3 Gnuts
- ◯ 3 Gnuts and 1/2 Gnut
- ◯ 4 Gnuts
- ◯ 4 Gnuts and 1/2 Gnut
- ◯ 5 Gnuts
- ◯ 5 Gnuts and 1/2 Gnut
- ◯ 6 Gnuts
- ◯ 6 Gnuts and 1/2 Gnut
- ◯ 7 Gnuts
- ◯ 7 Gnuts and 1/2 Gnut
- ◯ 8 Gnuts
- ◯ 8 Gnuts and 1/2 Gnut
- ◯ 9 Gnuts
- ◯ 9 Gnuts and 1/2 Gnut
- ◯ 10 Gnuts
- ◯ Don't know / Refuse to answer

((Face-to-face conversations refers to conversations at small distance (ie., not socially distanced) and without a face mask. Any number of peanuts is possible, irrespective of prior answers))

Own Mortality (Of any cause)

CX4: Now, out of 10, tell me the number of peanuts that reflects how likely you think it is that you will die within a five-year period beginning today. INTERVIEWER: Clarify: this question refers to mortality due to any cause, not just Covid-19.

- ◯ 0 Gnuts
- ◯ 1/2 Gnut
- ◯ 1 Gnut
- ◯ 1 Gnut and 1/2 Gnut
- ◯ 2 Gnuts
- ◯ 2 Gnuts and 1/2 Gnut
- ◯ 3 Gnuts
- ◯ 3 Gnuts and 1/2 Gnut
- ◯ 4 Gnuts
- ◯ 4 Gnuts and 1/2 Gnut
- ◯ 5 Gnuts
- ◯ 5 Gnuts and 1/2 Gnut
- ◯ 6 Gnuts
- ◯ 6 Gnuts and 1/2 Gnut
- ◯ 7 Gnuts
- ◯ 7 Gnuts and 1/2 Gnut
- ◯ 8 Gnuts
- ◯ 8 Gnuts and 1/2 Gnut
- ◯ 9 Gnuts
- ◯ 9 Gnuts and 1/2 Gnut
- ◯ 10 Gnuts
- ◯ Don't know / Refuse to answer

((Any number of peanuts is possible, irrespective of prior answers; Questions refers to mortality due to any cause, not just Covid-19. It includes dying from HIV, traffic accidents, heart attack, malaria or any other cause))

Covid-19 Expectations about Hypothetical Persons

CX5: Next, I am going to ask you some questions about an imaginary person living in the same context like you. Out of 10, tell me the number of peanuts that reflects how likely you think it is that ...

CX5a: ... a man your age who is healthy and does not have HIV will become infected with coronavirus in the next three (3) months?

- ◯ 0 Gnuts
- ◯ 1/2 Gnut
- ◯ 1 Gnut
- ◯ 1 Gnut and 1/2 Gnut
- ◯ 2 Gnuts
- ◯ 2 Gnuts and 1/2 Gnut
- ◯ 3 Gnuts
- ◯ 3 Gnuts and 1/2 Gnut
- ◯ 4 Gnuts
- ◯ 4 Gnuts and 1/2 Gnut
- ◯ 5 Gnuts
- ◯ 5 Gnuts and 1/2 Gnut
- ◯ 6 Gnuts
- ◯ 6 Gnuts and 1/2 Gnut
- ◯ 7 Gnuts
- ◯ 7 Gnuts and 1/2 Gnut
- ◯ 8 Gnuts
- ◯ 8 Gnuts and 1/2 Gnut
- ◯ 9 Gnuts
- ◯ 9 Gnuts and 1/2 Gnut
- ◯ 10 Gnuts
- ◯ Don't know / Refuse to answer

((Any number of peanuts is possible, irrespective of prior answers))

CX5a: ... a man your age who is healthy and does not have HIV will die within a five-year (5-year) period beginning today?
INTERVIEWER: Clarify: this question refers to mortality due to any cause, not just Covid-19.

- ◯ 0 Gnuts
- ◯ 1/2 Gnut
- ◯ 1 Gnut
- ◯ 1 Gnut and 1/2 Gnut
- ◯ 2 Gnuts
- ◯ 2 Gnuts and 1/2 Gnut
- ◯ 3 Gnuts
- ◯ 3 Gnuts and 1/2 Gnut
- ◯ 4 Gnuts
- ◯ 4 Gnuts and 1/2 Gnut
- ◯ 5 Gnuts
- ◯ 5 Gnuts and 1/2 Gnut
- ◯ 6 Gnuts
- ◯ 6 Gnuts and 1/2 Gnut
- ◯ 7 Gnuts
- ◯ 7 Gnuts and 1/2 Gnut
- ◯ 8 Gnuts
- ◯ 8 Gnuts and 1/2 Gnut
- ◯ 9 Gnuts
- ◯ 9 Gnuts and 1/2 Gnut
- ◯ 10 Gnuts
- ◯ Don't know / Refuse to answer

((Any number of peanuts is possible, irrespective of prior answers; questions again refers to mortality due to any cause, not just Covid-19. It includes dying from HIV, traffic accidents, heart attack, malaria or any other cause))

CX5a: ... a woman your age who is healthy and does not have HIV will become infected with coronavirus in the next three (3) months?	◯ 0 Gnuts<br>◯ 1/2 Gnut<br>◯ 1 Gnut<br>◯ 1 Gnut and 1/2 Gnut<br>◯ 2 Gnuts<br>◯ 2 Gnuts and 1/2 Gnut<br>◯ 3 Gnuts<br>◯ 3 Gnuts and 1/2 Gnut<br>◯ 4 Gnuts<br>◯ 4 Gnuts and 1/2 Gnut<br>◯ 5 Gnuts<br>◯ 5 Gnuts and 1/2 Gnut<br>◯ 6 Gnuts<br>◯ 6 Gnuts and 1/2 Gnut<br>◯ 7 Gnuts<br>◯ 7 Gnuts and 1/2 Gnut<br>◯ 8 Gnuts<br>◯ 8 Gnuts and 1/2 Gnut<br>◯ 9 Gnuts<br>◯ 9 Gnuts and 1/2 Gnut<br>◯ 10 Gnuts<br>◯ Don't know / Refuse to answer<br>((Any number of peanuts is possible, irrespective of prior answers))
CX5b: ... a woman your age who is healthy and does not have HIV will die within a five-year (5-year) period beginning today?<br>INTERVIEWER: Clarify: this question refers to mortality due to any cause, not just Covid-19.	◯ 0 Gnuts<br>◯ 1/2 Gnut<br>◯ 1 Gnut<br>◯ 1 Gnut and 1/2 Gnut<br>◯ 2 Gnuts<br>◯ 2 Gnuts and 1/2 Gnut<br>◯ 3 Gnuts<br>◯ 3 Gnuts and 1/2 Gnut<br>◯ 4 Gnuts<br>◯ 4 Gnuts and 1/2 Gnut<br>◯ 5 Gnuts<br>◯ 5 Gnuts and 1/2 Gnut<br>◯ 6 Gnuts<br>◯ 6 Gnuts and 1/2 Gnut<br>◯ 7 Gnuts<br>◯ 7 Gnuts and 1/2 Gnut<br>◯ 8 Gnuts<br>◯ 8 Gnuts and 1/2 Gnut<br>◯ 9 Gnuts<br>◯ 9 Gnuts and 1/2 Gnut<br>◯ 10 Gnuts<br>◯ Don't know / Refuse to answer<br>((Any number of peanuts is possible, irrespective of prior answers; questions again refers to mortality due to any cause, not just Covid-19. It includes dying from HIV, traffic accidents, heart attack, malaria or any other cause))

CX6: In the previous question, you told me [cv1_cx5a_male] to reflect the likelihood that an imaginary healthy man your age will become infected with coronavirus in the next three (3) months.

CX6a: How do you change your answer if this man also practices rigorous social distancing and washes his hands with soap regularly during the day?

◯ 0 Gnuts
◯ 1/2 Gnut
◯ 1 Gnut
◯ 1 Gnut and 1/2 Gnut
◯ 2 Gnuts
◯ 2 Gnuts and 1/2 Gnut
◯ 3 Gnuts
◯ 3 Gnuts and 1/2 Gnut
◯ 4 Gnuts
◯ 4 Gnuts and 1/2 Gnut
◯ 5 Gnuts
◯ 5 Gnuts and 1/2 Gnut
◯ 6 Gnuts
◯ 6 Gnuts and 1/2 Gnut
◯ 7 Gnuts
◯ 7 Gnuts and 1/2 Gnut
◯ 8 Gnuts
◯ 8 Gnuts and 1/2 Gnut
◯ 9 Gnuts
◯ 9 Gnuts and 1/2 Gnut
◯ 10 Gnuts
◯ Don't know / Refuse to answer
((Any number of peanuts is possible, irrespective of prior answers. Note: Social distancing refers to a broad set of behaviors aimed at maintaining a safe and appropriate distance from other people, and reducing social contacts more generally, to slow the spread of Covid-19))

CX6b: How do you change your answer if this man does none of these steps and continues to behave the way he did before the Covid-19 epidemic?

◯ 0 Gnuts
◯ 1/2 Gnut
◯ 1 Gnut
◯ 1 Gnut and 1/2 Gnut
◯ 2 Gnuts
◯ 2 Gnuts and 1/2 Gnut
◯ 3 Gnuts
◯ 3 Gnuts and 1/2 Gnut
◯ 4 Gnuts
◯ 4 Gnuts and 1/2 Gnut
◯ 5 Gnuts
◯ 5 Gnuts and 1/2 Gnut
◯ 6 Gnuts
◯ 6 Gnuts and 1/2 Gnut
◯ 7 Gnuts
◯ 7 Gnuts and 1/2 Gnut
◯ 8 Gnuts
◯ 8 Gnuts and 1/2 Gnut
◯ 9 Gnuts
◯ 9 Gnuts and 1/2 Gnut
◯ 10 Gnuts
◯ Don't know / Refuse to answer
((Any number of peanuts is possible, irrespective of prior answers))

CX6: In the previous question, you told me [cv1_cx5a_female] to reflect the likelihood that an imaginary healthy woman your age will become infected with coronavirus in the next three (3) months.

CX6a: How do you change your answer if this woman also practices rigorous social distancing and washes her hands with soap regularly during the day?	◯ 0 Gnuts<br>◯ 1/2 Gnut<br>◯ 1 Gnut<br>◯ 1 Gnut and 1/2 Gnut<br>◯ 2 Gnuts<br>◯ 2 Gnuts and 1/2 Gnut<br>◯ 3 Gnuts<br>◯ 3 Gnuts and 1/2 Gnut<br>◯ 4 Gnuts<br>◯ 4 Gnuts and 1/2 Gnut<br>◯ 5 Gnuts<br>◯ 5 Gnuts and 1/2 Gnut<br>◯ 6 Gnuts<br>◯ 6 Gnuts and 1/2 Gnut<br>◯ 7 Gnuts<br>◯ 7 Gnuts and 1/2 Gnut<br>◯ 8 Gnuts<br>◯ 8 Gnuts and 1/2 Gnut<br>◯ 9 Gnuts<br>◯ 9 Gnuts and 1/2 Gnut<br>◯ 10 Gnuts<br>◯ Don't know / Refuse to answer<br>((Any number of peanuts is possible, irrespective of prior answers. Note: Social distancing refers to a broad set of behaviors aimed at maintaining a safe and appropriate distance from other people, and reducing social contacts more generally, to slow the spread of Covid-19))
CX6b: How do you change your answer if this woman does none of these steps and continues to behave the way she did before the Covid-19 epidemic?	◯ 0 Gnuts<br>◯ 1/2 Gnut<br>◯ 1 Gnut<br>◯ 1 Gnut and 1/2 Gnut<br>◯ 2 Gnuts<br>◯ 2 Gnuts and 1/2 Gnut<br>◯ 3 Gnuts<br>◯ 3 Gnuts and 1/2 Gnut<br>◯ 4 Gnuts<br>◯ 4 Gnuts and 1/2 Gnut<br>◯ 5 Gnuts<br>◯ 5 Gnuts and 1/2 Gnut<br>◯ 6 Gnuts<br>◯ 6 Gnuts and 1/2 Gnut<br>◯ 7 Gnuts<br>◯ 7 Gnuts and 1/2 Gnut<br>◯ 8 Gnuts<br>◯ 8 Gnuts and 1/2 Gnut<br>◯ 9 Gnuts<br>◯ 9 Gnuts and 1/2 Gnut<br>◯ 10 Gnuts<br>◯ Don't know / Refuse to answer<br>((Any number of peanuts is possible, irrespective of prior answers))

Transmission and mortality risk within families

CX7: Finally, I am going to ask you two questions how coronavirus might affect your community. Who in your community do you think is most likely to ...

CX7a: ... get infected with coronavirus (Covid-19)?	◯ Young children (ages 5 years or younger)<br>◯ Older children (ages 6 to 17 years)<br>◯ Adults (ages 18-59 years)<br>◯ Older family members (ages 60 and above)<br>((Read responses))
CX7b: ... die if they become infected with coronavirus (Covid-19)?	◯ Young children (ages 5 years or younger)<br>◯ Older children (ages 6 to 17 years)<br>◯ Adults (ages 18-59 years)<br>◯ Older family members (ages 60 and above)<br>((Read responses))

Cv1 4b Covid19 Symptoms

Time ______________________

Respondent

[respname] (RespID: [respid])
([gender], about [age] years old; village: [villagename], region: [region], previous survey: [prior_surv_year], conducted in [prior_surv_language])

Symptoms of Respondent

SY1_intro: Next, I would like to ask you some questions about your health at the moment?

SY1a: Do you currently have a fever?
- ◯ Yes
- ◯ No

SY1b: When did it start?
- ◯ less than 1 day ago
- ◯ 1-2 days ago
- ◯ 3-6 days ago
- ◯ about 1 week ago
- ◯ about 2 weeks ago
- ◯ about 3 weeks ago
- ◯ about 1 month ago
- ◯ more than 1 month ago
- ◯ Don't remember

SY1c: Do you currently have a dry cough?
- ◯ Yes
- ◯ No

SY1d: When did it start?
- ◯ less than 1 day ago
- ◯ 1-2 days ago
- ◯ 3-6 days ago
- ◯ about 1 week ago
- ◯ about 2 weeks ago
- ◯ about 3 weeks ago
- ◯ about 1 month ago
- ◯ more than 1 month ago
- ◯ Don't remember

SY1e: Do you currently have shortness of breath, or have difficulties breathing?
- ◯ Yes
- ◯ No

SY1f: When did it start?
- ◯ less than 1 day ago
- ◯ 1-2 days ago
- ◯ 3-6 days ago
- ◯ about 1 week ago
- ◯ about 2 weeks ago
- ◯ about 3 weeks ago
- ◯ about 1 month ago
- ◯ more than 1 month ago
- ◯ Don't remember

SY1g: How likely do you think it is that you currently have coronavirus (Covid-19)	◯ Very likely<br>◯ Likely<br>◯ Not likely<br>◯ Very unlikely<br>◯ Don't know /refuse to answer<br>(Read Responses)
SY2a: Did you have any of these symptoms (fever, dry cough or shortness of breath) in the last few months?	◯ Yes<br>◯ No<br>(Question SY2a pertains to approximately the last six months. For respondents currently experiencing any symptoms, this prior spells will generally be some time in the past (two weeks, one month or more ago); for respondents who currently do not have any symptoms, this prior spell can be fairly recent (e.g., one week ago) or some time in the past (one to several months ago))
SY2b: When?	◯ about 1 week ago<br>◯ about 2 weeks ago<br>◯ about 3 weeks ago<br>◯ about 1 month ago<br>◯ about 2 months ago<br>◯ about 3 months ago<br>◯ more than 3 months ago<br>◯ Don't remember
SY2c: At that time, did you have fever, dry cough and shortness of breath at the same time?	◯ Yes<br>◯ No
SY2d: How likely do you think it is that you had coronavirus (Covid-19) at that time?	◯ Very likely<br>◯ Likely<br>◯ Not likely<br>◯ Very unlikely<br>◯ Don't know /refuse to answer<br>(Read Responses)
SY3a: Have you sought medical care because of these symptoms?	◯ Yes<br>◯ No
SY3b: Has a doctor, health care provider or traditional healer told you that you have or had coronavirus (Covid-19)?	◯ Yes<br>◯ No
SY3c: Was it based on a test for coronavirus (Covid-19)?	◯ Yes<br>◯ No

Symptoms of Household Members

SY4_Intro: Next, I am going to read the names of persons who lived in your household when we last surveyed you.

Please answer YES if the person currently has any of these three symptoms: fever, dry cough or difficulty breathing.

Answer NO if the persons is currently not affected by any of these symptoms.

If the person no longer lives in your household say NO LONGER LIVING HERE, and if the person has died, say HAS DIED.

SY4a: Does [m7_rname_1] have any of these symptoms: fever, dry cough or difficulty breathing? ([m7_relresp_1], [m7_rage_1])	◯ Yes -- Currently has fever, dry cough and/or difficulties breathing (any of the three)<br>◯ No -- does currently not have any of these symptoms (none of the three)<br>◯ Has died<br>◯ Not living / No longer living in this household<br>◯ Don't know / refuse to answer<br>(Select YES if (NAME) has ANY of these symptoms: fever, dry cough OR difficulty breathing)
SY5a: Does [m7_rname_1] currently have all three symptoms: fever, dry cough and difficulty breathing?	◯ Yes<br>◯ No
SY6a: How likely to you think that [m7_rname_1] currently has coronavirus?	◯ Very likely<br>◯ Likely<br>◯ Not likely<br>◯ Very unlikely<br>◯ Don't know /refuse to answer<br>(Read Responses)
SY7a: When did [m7_rname_1] die?	◯ Less than 1 month ago<br>◯ 1-3 months ago<br>◯ 4-6 months ago<br>◯ 7-12 months ago<br>◯ More than one year ago<br>◯ Don't know / don't recall
SY8a: Prior to his/her death, did [m7_rname_1] have fever, dry cough and difficulty breathing (all three symptoms)?	◯ Yes -- Had fever, dry cough and difficulties breathing (all three symptoms)<br>◯ No -- did not have all three of these symptoms<br>◯ Don't know / refuse to answer
SY9a: How likely do you think that [m7_rname_1] had coronavirus (Covid-19) when he/she died?	◯ Very likely<br>◯ Likely<br>◯ Not likely<br>◯ Very unlikely<br>◯ Don't know /refuse to answer<br>(Read Responses)
SY4b: Does [m7_rname_2] have any of these symptoms: fever, dry cough or difficulty breathing? ([m7_relresp_2], [m7_rage_2])	◯ Yes -- Currently has fever, dry cough and/or difficulties breathing (any of the three)<br>◯ No -- does currently not have any of these symptoms (none of the three)<br>◯ Has died<br>◯ Not living / No longer living in this household<br>◯ Don't know / refuse to answer<br>(Select YES if (NAME) has ANY of these symptoms: fever, dry cough OR difficulty breathing)
SY5b: Does [m7_rname_2] currently have all three symptoms: fever, dry cough and difficulty breathing?	◯ Yes<br>◯ No
SY6b: How likely to you think that [m7_rname_2] currently has coronavirus?	◯ Very likely<br>◯ Likely<br>◯ Not likely<br>◯ Very unlikely<br>◯ Don't know /refuse to answer<br>(Read Responses)

SY7b: When did [m7_rname_2] die?	◯ Less than 1 month ago<br>◯ 1-3 months ago<br>◯ 4-6 months ago<br>◯ 7-12 months ago<br>◯ More than one year ago<br>◯ Don't know / don't recall
SY8b: Prior to his/her death, did [m7_rname_2] have fever, dry cough and difficulty breathing (all three symptoms)?	◯ Yes -- Had fever, dry cough and difficulties breathing (all three symptoms)<br>◯ No -- did not have all three of these symptoms<br>◯ Don't know / refuse to answer
SY9b: How likely do you think that [m7_rname_2] had coronavirus (Covid-19) when he/she died?	◯ Very likely<br>◯ Likely<br>◯ Not likely<br>◯ Very unlikely<br>◯ Don't know /refuse to answer<br>(Read Responses)
SY4c: Does [m7_rname_3] have any of these symptoms: fever, dry cough or difficulty breathing? ([m7_relresp_3], [m7_rage_3])	◯ Yes -- Currently has fever, dry cough and/or difficulties breathing (any of the three)<br>◯ No -- does currently not have any of these symptoms (none of the three)<br>◯ Has died<br>◯ Not living / No longer living in this household<br>◯ Don't know / refuse to answer<br>(Select YES if (NAME) has ANY of these symptoms: fever, dry cough OR difficulty breathing)
SY5c: Does [m7_rname_3] currently have all three symptoms: fever, dry cough and difficulty breathing?	◯ Yes<br>◯ No
SY6c: How likely to you think that [m7_rname_3] currently has coronavirus?	◯ Very likely<br>◯ Likely<br>◯ Not likely<br>◯ Very unlikely<br>◯ Don't know /refuse to answer<br>(Read Responses)
SY7c: When did [m7_rname_3] die?	◯ Less than 1 month ago<br>◯ 1-3 months ago<br>◯ 4-6 months ago<br>◯ 7-12 months ago<br>◯ More than one year ago<br>◯ Don't know / don't recall
SY8c: Prior to his/her death, did [m7_rname_3] have fever, dry cough and difficulty breathing (all three symptoms)?	◯ Yes -- Had fever, dry cough and difficulties breathing (all three symptoms)<br>◯ No -- did not have all three of these symptoms<br>◯ Don't know / refuse to answer
SY9c: How likely do you think that [m7_rname_3] had coronavirus (Covid-19) when he/she died?	◯ Very likely<br>◯ Likely<br>◯ Not likely<br>◯ Very unlikely<br>◯ Don't know /refuse to answer<br>(Read Responses)

SY4d: Does [m7_rname_4] have any of these symptoms: fever, dry cough or difficulty breathing? ([m7_relresp_4], [m7_rage_4])	◯ Yes -- Currently has fever, dry cough and/or difficulties breathing (any of the three)<br>◯ No -- does currently not have any of these symptoms (none of the three)<br>◯ Has died<br>◯ Not living / No longer living in this household<br>◯ Don't know / refuse to answer<br>(Select YES if (NAME) has ANY of these symptoms: fever, dry cough OR difficulty breathing)
SY5d: Does [m7_rname_4] currently have all three symptoms: fever, dry cough and difficulty breathing?	◯ Yes<br>◯ No
SY6d: How likely to you think that [m7_rname_4] currently has coronavirus?	◯ Very likely<br>◯ Likely<br>◯ Not likely<br>◯ Very unlikely<br>◯ Don't know /refuse to answer<br>(Read Responses)
SY7d: When did [m7_rname_4] die?	◯ Less than 1 month ago<br>◯ 1-3 months ago<br>◯ 4-6 months ago<br>◯ 7-12 months ago<br>◯ More than one year ago<br>◯ Don't know / don't recall
SY8d: Prior to his/her death, did [m7_rname_4] have fever, dry cough and difficulty breathing (all three symptoms)?	◯ Yes -- Had fever, dry cough and difficulties breathing (all three symptoms)<br>◯ No -- did not have all three of these symptoms<br>◯ Don't know / refuse to answer
SY9d: How likely do you think that [m7_rname_4] had coronavirus (Covid-19) when he/she died?	◯ Very likely<br>◯ Likely<br>◯ Not likely<br>◯ Very unlikely<br>◯ Don't know /refuse to answer<br>(Read Responses)
SY4e: Does [m7_rname_5] have any of these symptoms: fever, dry cough or difficulty breathing? ([m7_relresp_5], [m7_rage_5])	◯ Yes -- Currently has fever, dry cough and/or difficulties breathing (any of the three)<br>◯ No -- does currently not have any of these symptoms (none of the three)<br>◯ Has died<br>◯ Not living / No longer living in this household<br>◯ Don't know / refuse to answer<br>(Select YES if (NAME) has ANY of these symptoms: fever, dry cough OR difficulty breathing)
SY5e: Does [m7_rname_5] currently have all three symptoms: fever, dry cough and difficulty breathing?	◯ Yes<br>◯ No
SY6e: How likely to you think that [m7_rname_5] currently has coronavirus?	◯ Very likely<br>◯ Likely<br>◯ Not likely<br>◯ Very unlikely<br>◯ Don't know /refuse to answer<br>(Read Responses)

SY7e: When did [m7_rname_5] die?	◯ Less than 1 month ago<br>◯ 1-3 months ago<br>◯ 4-6 months ago<br>◯ 7-12 months ago<br>◯ More than one year ago<br>◯ Don't know / don't recall
SY8e: Prior to his/her death, did [m7_rname_5] have fever, dry cough and difficulty breathing (all three symptoms)?	◯ Yes -- Had fever, dry cough and difficulties breathing (all three symptoms)<br>◯ No -- did not have all three of these symptoms<br>◯ Don't know / refuse to answer
SY9e: How likely do you think that [m7_rname_5] had coronavirus (Covid-19) when he/she died?	◯ Very likely<br>◯ Likely<br>◯ Not likely<br>◯ Very unlikely<br>◯ Don't know /refuse to answer<br>(Read Responses)
SY4f: Does [m7_rname_6] have any of these symptoms: fever, dry cough or difficulty breathing? ([m7_relresp_6], [m7_rage_6])	◯ Yes -- Currently has fever, dry cough and/or difficulties breathing (any of the three)<br>◯ No -- does currently not have any of these symptoms (none of the three)<br>◯ Has died<br>◯ Not living / No longer living in this household<br>◯ Don't know / refuse to answer<br>(Select YES if (NAME) has ANY of these symptoms: fever, dry cough OR difficulty breathing)
SY5f: Does [m7_rname_6] currently have all three symptoms: fever, dry cough and difficulty breathing?	◯ Yes<br>◯ No
SY6f: How likely to you think that [m7_rname_6] currently has coronavirus?	◯ Very likely<br>◯ Likely<br>◯ Not likely<br>◯ Very unlikely<br>◯ Don't know /refuse to answer<br>(Read Responses)
SY7f: When did [m7_rname_6] die?	◯ Less than 1 month ago<br>◯ 1-3 months ago<br>◯ 4-6 months ago<br>◯ 7-12 months ago<br>◯ More than one year ago<br>◯ Don't know / don't recall
SY8f: Prior to his/her death, did [m7_rname_6] have fever, dry cough and difficulty breathing (all three symptoms)?	◯ Yes -- Had fever, dry cough and difficulties breathing (all three symptoms)<br>◯ No -- did not have all three of these symptoms<br>◯ Don't know / refuse to answer
SY9f: How likely do you think that [m7_rname_6] had coronavirus (Covid-19) when he/she died?	◯ Very likely<br>◯ Likely<br>◯ Not likely<br>◯ Very unlikely<br>◯ Don't know /refuse to answer<br>(Read Responses)

SY4g: Does [m7_rname_7] have any of these symptoms: fever, dry cough or difficulty breathing? ([m7_relresp_7], [m7_rage_7])	◯ Yes -- Currently has fever, dry cough and/or difficulties breathing (any of the three)<br>◯ No -- does currently not have any of these symptoms (none of the three)<br>◯ Has died<br>◯ Not living / No longer living in this household<br>◯ Don't know / refuse to answer<br>(Select YES if (NAME) has ANY of these symptoms: fever, dry cough OR difficulty breathing)
SY5g: Does [m7_rname_7] currently have all three symptoms: fever, dry cough and difficulty breathing?	◯ Yes<br>◯ No
SY6g: How likely to you think that [m7_rname_7] currently has coronavirus?	◯ Very likely<br>◯ Likely<br>◯ Not likely<br>◯ Very unlikely<br>◯ Don't know /refuse to answer<br>(Read Responses)
SY7g: When did [m7_rname_7] die?	◯ Less than 1 month ago<br>◯ 1-3 months ago<br>◯ 4-6 months ago<br>◯ 7-12 months ago<br>◯ More than one year ago<br>◯ Don't know / don't recall
SY8g: Prior to his/her death, did [m7_rname_7] have fever, dry cough and difficulty breathing (all three symptoms)?	◯ Yes -- Had fever, dry cough and difficulties breathing (all three symptoms)<br>◯ No -- did not have all three of these symptoms<br>◯ Don't know / refuse to answer
SY9g: How likely do you think that [m7_rname_7] had coronavirus (Covid-19) when he/she died?	◯ Very likely<br>◯ Likely<br>◯ Not likely<br>◯ Very unlikely<br>◯ Don't know /refuse to answer<br>(Read Responses)
SY4h: Does [m7_rname_8] have any of these symptoms: fever, dry cough or difficulty breathing? ([m7_relresp_8], [m7_rage_8])	◯ Yes -- Currently has fever, dry cough and/or difficulties breathing (any of the three)<br>◯ No -- does currently not have any of these symptoms (none of the three)<br>◯ Has died<br>◯ Not living / No longer living in this household<br>◯ Don't know / refuse to answer<br>(Select YES if (NAME) has ANY of these symptoms: fever, dry cough OR difficulty breathing)
SY5h: Does [m7_rname_8] currently have all three symptoms: fever, dry cough and difficulty breathing?	◯ Yes<br>◯ No
SY6h: How likely to you think that [m7_rname_8] currently has coronavirus?	◯ Very likely<br>◯ Likely<br>◯ Not likely<br>◯ Very unlikely<br>◯ Don't know /refuse to answer<br>(Read Responses)

SY7h: When did [m7_rname_8] die?	◯ Less than 1 month ago<br>◯ 1-3 months ago<br>◯ 4-6 months ago<br>◯ 7-12 months ago<br>◯ More than one year ago<br>◯ Don't know / don't recall
SY8h: Prior to his/her death, did [m7_rname_8] have fever, dry cough and difficulty breathing (all three symptoms)?	◯ Yes -- Had fever, dry cough and difficulties breathing (all three symptoms)<br>◯ No -- did not have all three of these symptoms<br>◯ Don't know / refuse to answer
SY9h: How likely do you think that [m7_rname_8] had coronavirus (Covid-19) when he/she died?	◯ Very likely<br>◯ Likely<br>◯ Not likely<br>◯ Very unlikely<br>◯ Don't know /refuse to answer<br>(Read Responses)
SY4i: Does [m7_rname_9] have any of these symptoms: fever, dry cough or difficulty breathing? ([m7_relresp_9], [m7_rage_9])	◯ Yes -- Currently has fever, dry cough and/or difficulties breathing (any of the three)<br>◯ No -- does currently not have any of these symptoms (none of the three)<br>◯ Has died<br>◯ Not living / No longer living in this household<br>◯ Don't know / refuse to answer<br>(Select YES if (NAME) has ANY of these symptoms: fever, dry cough OR difficulty breathing)
SY5i: Does [m7_rname_9] currently have all three symptoms: fever, dry cough and difficulty breathing?	◯ Yes<br>◯ No
SY6i: How likely to you think that [m7_rname_9] currently has coronavirus?	◯ Very likely<br>◯ Likely<br>◯ Not likely<br>◯ Very unlikely<br>◯ Don't know /refuse to answer<br>(Read Responses)
SY7i: When did [m7_rname_9] die?	◯ Less than 1 month ago<br>◯ 1-3 months ago<br>◯ 4-6 months ago<br>◯ 7-12 months ago<br>◯ More than one year ago<br>◯ Don't know / don't recall
SY8i: Prior to his/her death, did [m7_rname_9] have fever, dry cough and difficulty breathing (all three symptoms)?	◯ Yes -- Had fever, dry cough and difficulties breathing (all three symptoms)<br>◯ No -- did not have all three of these symptoms<br>◯ Don't know / refuse to answer
SY9i: How likely do you think that [m7_rname_9] had coronavirus (Covid-19) when he/she died?	◯ Very likely<br>◯ Likely<br>◯ Not likely<br>◯ Very unlikely<br>◯ Don't know /refuse to answer<br>(Read Responses)

SY4j: Does [m7_rname_10] have any of these symptoms: fever, dry cough or difficulty breathing? ([m7_relresp_10], [m7_rage_10])	◯ Yes -- Currently has fever, dry cough and/or difficulties breathing (any of the three)<br>◯ No -- does currently not have any of these symptoms (none of the three)<br>◯ Has died<br>◯ Not living / No longer living in this household<br>◯ Don't know / refuse to answer<br>(Select YES if (NAME) has ANY of these symptoms: fever, dry cough OR difficulty breathing)
SY5j: Does [m7_rname_10] currently have all three symptoms: fever, dry cough and difficulty breathing?	◯ Yes<br>◯ No
SY6j: How likely to you think that [m7_rname_10] currently has coronavirus?	◯ Very likely<br>◯ Likely<br>◯ Not likely<br>◯ Very unlikely<br>◯ Don't know /refuse to answer<br>(Read Responses)
SY7j: When did [m7_rname_10] die?	◯ Less than 1 month ago<br>◯ 1-3 months ago<br>◯ 4-6 months ago<br>◯ 7-12 months ago<br>◯ More than one year ago<br>◯ Don't know / don't recall
SY8j: Prior to his/her death, did [m7_rname_10] have fever, dry cough and difficulty breathing (all three symptoms)?	◯ Yes -- Had fever, dry cough and difficulties breathing (all three symptoms)<br>◯ No -- did not have all three of these symptoms<br>◯ Don't know / refuse to answer
SY9j: How likely do you think that [m7_rname_10] had coronavirus (Covid-19) when he/she died?	◯ Very likely<br>◯ Likely<br>◯ Not likely<br>◯ Very unlikely<br>◯ Don't know /refuse to answer<br>(Read Responses)
SY4k: Does [m7_rname_11] have any of these symptoms: fever, dry cough or difficulty breathing? ([m7_relresp_11], [m7_rage_11])	◯ Yes -- Currently has fever, dry cough and/or difficulties breathing (any of the three)<br>◯ No -- does currently not have any of these symptoms (none of the three)<br>◯ Has died<br>◯ Not living / No longer living in this household<br>◯ Don't know / refuse to answer<br>(Select YES if (NAME) has ANY of these symptoms: fever, dry cough OR difficulty breathing)
SY5k: Does [m7_rname_11] currently have all three symptoms: fever, dry cough and difficulty breathing?	◯ Yes<br>◯ No
SY6k: How likely to you think that [m7_rname_11] currently has coronavirus?	◯ Very likely<br>◯ Likely<br>◯ Not likely<br>◯ Very unlikely<br>◯ Don't know /refuse to answer<br>(Read Responses)

SY7k: When did [m7_rname_11] die?	◯ Less than 1 month ago<br>◯ 1-3 months ago<br>◯ 4-6 months ago<br>◯ 7-12 months ago<br>◯ More than one year ago<br>◯ Don't know / don't recall
SY8k: Prior to his/her death, did [m7_rname_11] have fever, dry cough and difficulty breathing (all three symptoms)?	◯ Yes -- Had fever, dry cough and difficulties breathing (all three symptoms)<br>◯ No -- did not have all three of these symptoms<br>◯ Don't know / refuse to answer
SY9k: How likely do you think that [m7_rname_11] had coronavirus (Covid-19) when he/she died?	◯ Very likely<br>◯ Likely<br>◯ Not likely<br>◯ Very unlikely<br>◯ Don't know /refuse to answer<br>(Read Responses)
SY4l: Does [m7_rname_12] have any of these symptoms: fever, dry cough or difficulty breathing? ([m7_relresp_12], [m7_rage_12])	◯ Yes -- Currently has fever, dry cough and/or difficulties breathing (any of the three)<br>◯ No -- does currently not have any of these symptoms (none of the three)<br>◯ Has died<br>◯ Not living / No longer living in this household<br>◯ Don't know / refuse to answer<br>(Select YES if (NAME) has ANY of these symptoms: fever, dry cough OR difficulty breathing)
SY5l: Does [m7_rname_12] currently have all three symptoms: fever, dry cough and difficulty breathing?	◯ Yes<br>◯ No
SY6l: How likely to you think that [m7_rname_12] currently has coronavirus?	◯ Very likely<br>◯ Likely<br>◯ Not likely<br>◯ Very unlikely<br>◯ Don't know /refuse to answer<br>(Read Responses)
SY7l: When did [m7_rname_12] die?	◯ Less than 1 month ago<br>◯ 1-3 months ago<br>◯ 4-6 months ago<br>◯ 7-12 months ago<br>◯ More than one year ago<br>◯ Don't know / don't recall
SY8l: Prior to his/her death, did [m7_rname_12] have fever, dry cough and difficulty breathing (all three symptoms)?	◯ Yes -- Had fever, dry cough and difficulties breathing (all three symptoms)<br>◯ No -- did not have all three of these symptoms<br>◯ Don't know / refuse to answer
SY9l: How likely do you think that [m7_rname_12] had coronavirus (Covid-19) when he/she died?	◯ Very likely<br>◯ Likely<br>◯ Not likely<br>◯ Very unlikely<br>◯ Don't know /refuse to answer<br>(Read Responses)

SY4m: Does [m7_rname_13] have any of these symptoms: fever, dry cough or difficulty breathing? ([m7_relresp_13], [m7_rage_13])	◯ Yes -- Currently has fever, dry cough and/or difficulties breathing (any of the three)<br>◯ No -- does currently not have any of these symptoms (none of the three)<br>◯ Has died<br>◯ Not living / No longer living in this household<br>◯ Don't know / refuse to answer<br>(Select YES if (NAME) has ANY of these symptoms: fever, dry cough OR difficulty breathing)
SY5m: Does [m7_rname_13] currently have all three symptoms: fever, dry cough and difficulty breathing?	◯ Yes<br>◯ No
SY6m: How likely to you think that [m7_rname_13] currently has coronavirus?	◯ Very likely<br>◯ Likely<br>◯ Not likely<br>◯ Very unlikely<br>◯ Don't know /refuse to answer<br>(Read Responses)
SY7m: When did [m7_rname_13] die?	◯ Less than 1 month ago<br>◯ 1-3 months ago<br>◯ 4-6 months ago<br>◯ 7-12 months ago<br>◯ More than one year ago<br>◯ Don't know / don't recall
SY8m: Prior to his/her death, did [m7_rname_13] have fever, dry cough and difficulty breathing (all three symptoms)?	◯ Yes -- Had fever, dry cough and difficulties breathing (all three symptoms)<br>◯ No -- did not have all three of these symptoms<br>◯ Don't know / refuse to answer
SY9m: How likely do you think that [m7_rname_13] had coronavirus (Covid-19) when he/she died?	◯ Very likely<br>◯ Likely<br>◯ Not likely<br>◯ Very unlikely<br>◯ Don't know /refuse to answer<br>(Read Responses)
SY4n: Does [m7_rname_14] have any of these symptoms: fever, dry cough or difficulty breathing? ([m7_relresp_14], [m7_rage_14])	◯ Yes -- Currently has fever, dry cough and/or difficulties breathing (any of the three)<br>◯ No -- does currently not have any of these symptoms (none of the three)<br>◯ Has died<br>◯ Not living / No longer living in this household<br>◯ Don't know / refuse to answer<br>(Select YES if (NAME) has ANY of these symptoms: fever, dry cough OR difficulty breathing)
SY5n: Does [m7_rname_14] currently have all three symptoms: fever, dry cough and difficulty breathing?	◯ Yes<br>◯ No
SY6n: How likely to you think that [m7_rname_14] currently has coronavirus?	◯ Very likely<br>◯ Likely<br>◯ Not likely<br>◯ Very unlikely<br>◯ Don't know /refuse to answer<br>(Read Responses)

SY7n: When did [m7_rname_14] die?	◯ Less than 1 month ago<br>◯ 1-3 months ago<br>◯ 4-6 months ago<br>◯ 7-12 months ago<br>◯ More than one year ago<br>◯ Don't know / don't recall
SY8n: Prior to his/her death, did [m7_rname_14] have fever, dry cough and difficulty breathing (all three symptoms)?	◯ Yes -- Had fever, dry cough and difficulties breathing (all three symptoms)<br>◯ No -- did not have all three of these symptoms<br>◯ Don't know / refuse to answer
SY9n: How likely do you think that [m7_rname_14] had coronavirus (Covid-19) when he/she died?	◯ Very likely<br>◯ Likely<br>◯ Not likely<br>◯ Very unlikely<br>◯ Don't know /refuse to answer<br>(Read Responses)
SY4o: Does [m7_rname_15] have any of these symptoms: fever, dry cough or difficulty breathing? ([m7_relresp_15], [m7_rage_15])	◯ Yes -- Currently has fever, dry cough and/or difficulties breathing (any of the three)<br>◯ No -- does currently not have any of these symptoms (none of the three)<br>◯ Has died<br>◯ Not living / No longer living in this household<br>◯ Don't know / refuse to answer<br>(Select YES if (NAME) has ANY of these symptoms: fever, dry cough OR difficulty breathing)
SY5o: Does [m7_rname_15] currently have all three symptoms: fever, dry cough and difficulty breathing?	◯ Yes<br>◯ No
SY6o: How likely to you think that [m7_rname_15] currently has coronavirus?	◯ Very likely<br>◯ Likely<br>◯ Not likely<br>◯ Very unlikely<br>◯ Don't know /refuse to answer<br>(Read Responses)
SY7o: When did [m7_rname_15] die?	◯ Less than 1 month ago<br>◯ 1-3 months ago<br>◯ 4-6 months ago<br>◯ 7-12 months ago<br>◯ More than one year ago<br>◯ Don't know / don't recall
SY8o: Prior to his/her death, did [m7_rname_15] have fever, dry cough and difficulty breathing (all three symptoms)?	◯ Yes -- Had fever, dry cough and difficulties breathing (all three symptoms)<br>◯ No -- did not have all three of these symptoms<br>◯ Don't know / refuse to answer
SY9o: How likely do you think that [m7_rname_15] had coronavirus (Covid-19) when he/she died?	◯ Very likely<br>◯ Likely<br>◯ Not likely<br>◯ Very unlikely<br>◯ Don't know /refuse to answer<br>(Read Responses)
SY10: Sum of HH members potentially sick with Covid-19	______________________________

Cv1 5a Final Questions

Time ______________________

Respondent

[respname] (RespID: [respid])
([gender], about [age] years old; village: [villagename], region: [region], previous survey: [prior_surv_year], conducted in [prior_surv_language])

Trust in Institutions

TR1a: When you have concerns about your own health, how much do you trust the health workers (doctors, nurses, HSAs) that service your village?

- ◯ Strongly distrust
- ◯ Somewhat distrust
- ◯ Neither trust nor distrust
- ◯ Somewhat trust
- ◯ Strongly trust

(Read Responses)

TR1b: How much do you trust these health workers (doctors, nurses, HSAs) to do what it takes to minimize the negative effect of the Covid-19 epidemic?

- ◯ Strongly distrust
- ◯ Somewhat distrust
- ◯ Neither trust nor distrust
- ◯ Somewhat trust
- ◯ Strongly trust

TR2: How factually truthful do you think your country's government has been about the Covid-19 epidemic?

- ◯ Very untruthful
- ◯ Somewhat untruthful
- ◯ Neither truthful nor untruthful
- ◯ Somewhat truthful
- ◯ Very truthful

(Read Responses)

Cv1 6a Survey Completion

Time

____

Respondent

[respname] (RespID: [respid])
([gender], about [age] years old; village: [villagename], region: [region], previous survey: [prior_surv_year], conducted in [prior_surv_language])

Survey Outcome Information

Time interview ended:

____
(Day Month Year Hours:Min, entered as 15-04-2018 17:45)

INTERVIEWER: Thank the respondent: Thank you for your participation in this study.

Information statement to respondents about COVID-19

INTERVIEWER: Read the following information about COVID-19 to the respondent after completion of the interview

During the interview, we talked about a new health issue that is occurring in Malawi, COVID-19. I would like to provide you with more information about this, so that you can use it to stay safe.

COVID-19 is a virus that can be transmitted between people, through droplets that people may cough or sneeze. It can also be transmitted from surfaces that people may have touched.

After infection, symptoms appear usually within 2 to 10 days. They may include fever, dry cough, and difficulty in breathing, among others. At the moment, there is no medicine or vaccine against COVID-19.

The large majority of people infected with COVID-19 will recover from the disease, but a small proportion will require hospitalization. In some instances, COVID-19 might also be life threatening.

There are precautions you can take to protect yourself and others against COVID-19. You can:
- Stop handshakes
- Maintain social distance with people;
- Stop touching eyes, nose and mouth;
- Practice cough hygiene by covering mouth and nose with tissue, sleeve or flexed elbow when coughing or sneezing;
- Seek medical care whenever feeling unwell;
- Wash your hands regularly with soap.

If you need additional information, you can either call the toll-free hotline established by the Ministry of Health. Simply dial 54747 on your phone. You can also visit the facebook page of the Ministry of Health if you have access to the internet.

Some of the symptoms that you reported for yourself or members of your household can occur if you are infected with the virus that causes covid19. Even though the symptoms of Covid19 are mild for most people, it can in some cases lead to severe complications. We therefore advise you to dial the free number 54747 to receive more information on how to seek care.

INTERVIEWER: Confirm that the above information about Covid-19 was read to the respondent	◯ Yes<br>◯ No

Information about airtime compensation

CI9: To appreciate your participation in this survey, you will be provided with airtime for 1,000 Malawian Kwachas. The airtime will be sent directly to your phone in the next days.

INTERVIEWER: Select the phone number to which the airtime is to be transfered:	◯ [cv1_phone1]<br>◯ [cv1_phone2]<br>◯ [cv1_phone3]<br>◯ [cv1_phone4]<br>◯ [cv1_phone5]<br>◯ [cv1_phone6]<br>◯ Other, specify
Other phone for airtime transfer	__________________________
Whose Phone is this:	◯ Respondent ([respname])<br>◯ Child<br>◯ Spouse<br>◯ Household member<br>◯ Relative outside of household<br>◯ Friend/neighbor<br>◯ Other
Name of owner of phone	__________________________

INTERVIEWER: Confirm that the below is the correct number to transfer the airtime

[cv1_phone1] (belongs to [cv1_phone1_name])	◯ Correct<br>◯ Incorrect --> update in CI9
[cv1_phone2] (belongs to [cv1_phone2_name])	◯ Correct<br>◯ Incorrect --> update in CI9
[cv1_phone3] (belongs to [cv1_phone3_name])	◯ Correct<br>◯ Incorrect --> update in CI9
[cv1_phone4] (belongs to [cv1_phone4_name])	◯ Correct<br>◯ Incorrect --> update in CI9
[cv1_phone5] (belongs to [cv1_phone5_name])	◯ Correct<br>◯ Incorrect --> update in CI9
[cv1_phone6] (belongs to [cv1_phone6_name])	◯ Correct<br>◯ Incorrect --> update in CI9
[cv1_ci9_other]	◯ Correct<br>◯ Incorrect --> update in CI9

INTERVIEWER: Select phone for airtime transfer before proceeding

Mental Health Resources for Respondents Expressing Self-Harming or Suicidal Thoughts

INTERIVEWER: Give respondent information about mental health resources (ONLY respondents residing in Mchinji, Rumphi or Balaka)

◯ Check

Complete Phone Call

INTERVIEWER: Ask if respondent has any questions. Thank again the respondent for participation. End the phone call.

____
(Day Month Year Hours:Min, entered as 15-04-2018 17:45)

INTERVIEWER: Confirm the phone number that was used for this phone call

[cv1_phone1] (belongs to [cv1_phone1_name])

◯ Yes
◯ No

[cv1_phone2] (belongs to [cv1_phone2_name])

◯ Yes
◯ No

[cv1_phone3] (belongs to [cv1_phone3_name])

◯ Yes
◯ No

[cv1_phone4] (belongs to [cv1_phone4_name])

◯ Yes
◯ No

[cv1_phone5] (belongs to [cv1_phone5_name])

◯ Yes
◯ No

[cv1_phone6] (belongs to [cv1_phone6_name])

◯ Yes
◯ No

Other phone:

____

Interviewer Questionnaire

Soon after the interview, please answer the following questions

I6: Degree of cooperation

◯ Bad
◯ Average
◯ Good
◯ Very good
(Note to Interviewer: indicate the degree of cooperation compared to other respondents, not overall degree of cooperation.)

I9: In general, how would you rate the respondent's ability to understand the survey questions?

◯ Excellent
◯ Very Good
◯ Good
◯ Fair
◯ Poor

I9a: Did the respondent skip or refuse to answer questions during the survey?

- ◯ Respondent answered all questions
- ◯ Respondent refused or skipped a few questions (fewer than 10)
- ◯ Respondent refused or skipped some questions (about 10-20 in total)
- ◯ Respondent refused or skipped many questions (more than 20 in total)
- ◯ Respondent stopped survey during interview and refused to continue

I11: During the interview, did you experience any technical difficulties. Check all that apply:

a: Could not hear respondent well all the time

- ◯ Yes
- ◯ No

b: Phone connection got disrupted / call got dropped

- ◯ Yes
- ◯ No

c: Interviewer phone problems (if yes, report details to IKI team)

- ◯ Yes
- ◯ No

I10: Do you have any other comment about the interview? Please describe any uncomfortable moments that might have occurred during this interview.

____

Self-Harming or Suicidal Thoughts

INTERIVEWER: Report to IKI team that respondent expressed self-harming or suicidal thoughts!

- ◯ Check

Questionnaire Quality Control

m11 Supervisor Check: Time

____

Supervisor Name

- ◯ List of Interviewers

Write supervisor name:

____

Supervisor date

____
(Day Month Year Hours:Min, entered as 15-04-2018 17:45)

Upenn team comments:

____

Supervisor: Final Survey Outcome

Survey outcome

- ◯ Success (Completed Survey)
- ◯ Problematic/incomplete survey
- ◯ Respondent not available (rescheduled)
- ◯ Refusal
- ◯ Reached informant, need to make more phone calls to reach respondent
- ◯ Reached informant, no more phone calls (case to be closed)
- ◯ Informant not available (rescheduled)
- ◯ Obtained alternative phone number
- ◯ No answer
- ◯ No connection / phone disconnected
- ◯ Reached wrong person / respondent not known at number
- ◯ Other (leave comments)

Is the case closed?

- ◯ No, we need make more calls to respondent/informant
- ◯ Yes: Case is closed -- no more calls/calls attempts

Date when case was closed:

____
(Day Month Year, entered as 15-04-2018)

Additional comments on survey outcome:

____
(Enter any other comments on survey outcome and/or possibility to complete an incomplete survey)

RedCap Dictionary Version

XXXXX
