# Supplementary material for: Is experience of the HIV/AIDS epidemic associated with responses to COVID-19? Evidence from the Rural Malawi
Source: PLoS One. 2023 Oct 25;18(10):e0292378. doi: 10.1371/journal.pone.0292378 (PMC10599567; doi:10.1371/journal.pone.0292378)
Supplement: Attachment — Submitted filename: PONE-D-23-07190.docx [file pone.0292378.s003.docx]

MANUSCRIPT NUMBER: PONE-D-23-07190

MANUSCRIPT TITLE: Is Experience of the HIV/AIDS Epidemic Associated with Responses to COVId-19? Evidence from Rural Malawi

Authors need to mention the ethical review of this study and if possibly attached the ethical letter acquired from the ethical board.

The study Lack figures to show relationships and correlations.

Structured questions regarding the interview and other form of data collection strategy must be submitted or attached.

Authors need to discuss about the Data analysis in the methodology. For example, the use of descriptive statistics, ANOVA etc and the software use.

In the theoretical framework, authors can also highlighted the differences between COVID-19 pandemic and HIV/AIDS epidemic in Malawi using statistical numbers like number of cases recorded, mortality, recovery etc. using secondary data is highly recommended.

Authors can list the contribution of the study at the end of the introductory section.

The abstract section can be modify by providing simple introduction to the disease before laying out the research gap/challenges.

This sentence in the abstract should be modify in this manner: Based on the outcome of the data analysis using …. Statistical approach, we found out that the experience of HIV…’

Number of COVID-19 cases should be updated as well as recovery and deaths.

The background of the study should be merged with the introduction part.
