# Supplementary material for: Is experience of the HIV/AIDS epidemic associated with responses to COVID-19? Evidence from the Rural Malawi
Source: PLoS One. 2023 Oct 25;18(10):e0292378. doi: 10.1371/journal.pone.0292378 (PMC10599567; doi:10.1371/journal.pone.0292378)
Supplement: Attachment — Submitted filename: Responses to Reviewers comments.docx [file pone.0292378.s005.docx]

We appreciate the final comments from the reviewer and have addressed in our updated version of the manuscript. Specifically, the reviewer noted that:

**“All comments have been addressed. The authors only need to do thorough editing of the manuscript for grammar and typo errors.”**

We have thoroughly reviewed the manuscript to correct grammatical errors and typos, so the final version is clear of any such issues.

We have also included our responses to earlier comments from both reviewers below, as a record of this previous effort.

**Prior responses to reviewers’ comments**

We appreciate the reviewers’ comments, as they have helped to strengthen this research. Below we list our responses to each comment.

**Reviewer #1**

**Abstract: The abstract would have been sufficient if presented in few sentences per section based on IMRAD postulations. It will be the ideal to used Introduction, Methodology, Results and Discussion techniques, rather than a mixed-up presentation as did by the authors.**

We appreciate the suggestion and have reformatted our abstract into the following sections: Introduction, Methods, Results, and Conclusions.

**Background/Introduction: Paragraph 1: The sentence topic sound like a mixed-up not tallying with the entire paragraph. The information presented in the paragraph lacks clarity, if compared with the sentence topic.**

We agree and have edited this paragraph to clarify our point. Essentially, we are saying that sub-Saharan Africa has a lower COVID-19 disease burden than other regions- this is now our topic sentence.

**Methodology: Malawi Longitudinal Study of Families and Health (MLSFH) should be explain using simple terms for ease of understandings and reflection for a repeat study using similar approach.**

We have edited the first paragraph of the Methods section to describe the MLSFH more clearly.

**Reviewer #2**

**Authors need to mention the ethical review of this study and if possibly attached the ethical letter acquired from the ethical board.**

This study was a secondary data analysis of de-identified data. As a result, ethical approval was not required for this study. However, the broader MLSFH study did receive ethical approval to collect the data used in this analysis and the authors signed a data use agreement prior to conducting this study. We have added a description of the MLSFH ethical approval to the manuscript in the “Data” section.

**The study lacks figures to show relationships and correlations.**

We appreciate this suggestion. However, given the amount of information shown in this manuscript, we feel that the most effective way to present our results is in a table format instead of using figures. But we are open to suggestions for figures to use if this reviewer has something in mind.

**Structured questions regarding the interview and other form of data collection strategy must be submitted or attached.**

We have added a link to the MLSFH survey instruments from 2006 and 2008 (available online at <https://web.sas.upenn.edu/malawiresearch/documentation/questionnaires/>). We have also added the MLSFH COVID-19 survey as appendix materials for this research. We note this in the “Measures” section of this manuscript. We also provide a reference for a manuscript that provides extensive detail about the MLSFH data collection strategy (Kohler et al. 2015).

**Authors need to discuss about the Data analysis in the methodology. For example, the use of descriptive statistics, ANOVA etc and the software use.**

We added a description of our data analysis to the “Analytic Methods” section. This describes our analytic approach and also includes a description of the software we used for analysis (Stata version 15).

**In the theoretical framework, authors can also highlighted the differences between COVID-19 pandemic and HIV/AIDS epidemic in Malawi using statistical numbers like number of cases recorded, mortality, recovery etc. using secondary data is highly recommended.**

We appreciate this suggestion and agree that it’s important to note the differences between COVID-19 and HIV/AIDS. We have added this to the paragraph to the theoretical framework section explaining why there may not be any connection between COVID-19 and HIV/AIDS, due to the differences in prevalence and mortality.

**Authors can list the contribution of the study at the end of the introductory section.**

We appreciate this suggestion and have added the author contributions to this manuscript. According to formatting guidelines for PLoS One, these appear just before the references section of the manuscript.

**The abstract section can be modify by providing simple introduction to the disease before laying out the research gap/challenges.**

We have added a sentence to the abstract to introduce COVID-19, and have also added more information about COVID-19 to the theoretical framework section, to distinguish between COVID-19 and HIV/AIDS.

**This sentence in the abstract should be modify in this manner: Based on the outcome of the data analysis using …. Statistical approach, we found out that the experience of HIV…’**

We appreciate this comment and have rephrased this sentence as “Based on the outcome of the data analysis using multivariable regression models, we found that the experience of HIV-related mortality…”

**Number of COVID-19 cases should be updated as well as recovery and deaths.**

We have updated the number of COVID-19 cases and deaths in Malawi according to 2023 data (through May).

**The background of the study should be merged with the introduction part.**

We have merged the background and introduction sections, with the research goals coming at the end of this section, as is the typical format.
